# RPL22L1 induction in colorectal cancer is associated with poor prognosis and 5-FU resistance

**DOI:** 10.1371/journal.pone.0222392

**Published:** 2019-10-03

**Authors:** Shuyun Rao, Suraj Peri, Jens Hoffmann, Kathy Q. Cai, Bryan Harris, Michele Rhodes, Denise C. Connolly, Joseph R. Testa, David L. Wiest

**Affiliations:** 1 Center for Translational Medicine, Department of Surgery, George Washington University, Washington, DC, United States of America; 2 Blood Cell Development and Function Program, Fox Chase Cancer Center, Philadelphia, PA, United States of America; 3 Experimental Pharmacology & Oncology Berlin-Buch GMBH, Berlin-Buch, Germany; 4 Cancer Biology Program, Fox Chase Cancer Center, Philadelphia, PA, United States of America; 5 Molecular Therapeutics Program, Fox Chase Cancer Center, Philadelphia, PA, United States of America; National Cancer Center, JAPAN

## Abstract

We have previously demonstrated that loss of the tumor suppressive activity of ribosomal protein (RP) RPL22 predisposes to development of leukemia in mouse models and aggressive disease in human patients; however, the role of RPL22 in solid tumors, specifically colorectal cancer (CRC), had not been explored. We report here that *RPL22* is either deleted or mutated in 36% of CRC and provide new insights into its mechanism of action. Indeed, *Rpl22* inactivation causes the induction of its highly homologous paralog, RPL22L1, which serves as a driver of cell proliferation and anchorage-independent growth in CRC cells. Moreover, RPL22L1 protein is highly expressed in patient CRC samples and correlates with poor survival. Interestingly, the association of high RPL22L1 expression with poor prognosis appears to be linked to resistance to 5-Fluorouracil, which is a core component of most CRC therapeutic regimens. Indeed, in an avatar trial, we found that human CRC samples that were unresponsive to 5-Fluorouracil in patient-derived xenografts exhibited elevated expression levels of RPL22L1. This link between RPL22L1 induction and 5-Fluorouracil resistance appears to be causal, because ectopic expression or knockdown of RPL22L1 in cell lines increases and decreases 5-Fluorouracil resistance, respectively, and this is associated with changes in expression of the DNA-repair genes, MGMT and MLH1. In summary, our data suggest that RPL22L1 might be a prognostic marker in CRC and predict 5-FU responsiveness.

## Introduction

Emerging evidence suggests that some ribosomal proteins (RP) play critical, but poorly understood roles in disease [1, 2], including bone marrow failure syndromes featuring an increased predisposition to cancer. RPL22 is an RNA-binding RP that is a component of the 60S ribosomal subunit, but its physiological role in normal development and its contribution to disease remains to be established. We have previously shown that RPL22 is dispensable for global, cap-dependent translation, but plays a critical role in regulating normal hematopoiesis and T cell development [2–4]. Recently we also discovered that RPL22 functions as a haploinsufficient tumor suppressor in T-cell acute lymphoma/leukemia (T-ALL)[2]. Loss of one copy of *Rpl22*, which does not affect normal T cell development, markedly accelerates the development of thymic lymphoma driven by a MyrAkt2 transgene [2, 5]. RPL22’s role as a tumor suppressor is further supported by our observation that monoallelic inactivation or deletion of *RPL22* was observed in human T-ALL and associated with reduced survival; *RPL22* inactivation has also been observed in a variety of solid tumors, particularly colorectal cancer (CRC) [6–10].

CRC is the third leading cause of cancer related deaths in the US and the third most commonly diagnosed cancer in the world [11]. Although surgery and chemotherapy have been shown to be effective in early stage CRC (stage I and II), treatment of advanced stage CRC (III and IV) is very challenging, particularly in cases with liver and lung metastasis. Currently, 5-Fluorouracil (5-FU), in combination with other agents such as oxaliplatin, has been shown to improve overall survival in CRC patients with advanced disease [12–14]. However, toxicity, drug resistance, and disease recurrence remain significant problems. In order to improve the efficacy of CRC treatment, it is critical to identify those patients likely to be resistant to the current standard of care, which requires the discovery of effective prognostic markers in CRC. Here, we report our finding that the loss of *Rpl22*, which serves as a tumor suppressor in some hematologic malignancies, results in the induction of its paralog RPL22L1. Thus, RPL22L1 induction may serve as a surrogate biomarker for *RPL22* loss. Moreover, RPL22L1 induction in CRC promotes proliferation and anchorage-independent growth. RPL22L1 overexpression is associated with poor survival in CRC patients, and our avatar trial suggests that this may be related to 5-FU resistance. Finally, bioinformatic and molecular analysis revealed that RPL22L1 may regulate 5-FU resistance through effects on the DNA repair proteins MGMT and MLH1.

## Materials and methods

### Ethics statement

This study was performed in strict accordance with the Guide for the Care and Use of Laboratory Animals of National Institutes of health and the guidelines established by the Institutional Animal Care and Use Committees for animal experiments. All animal protocols were approved by the Institute of Animal Care and Use Committee at Fox Chase Cancer Center (02–11). The *Rpl22*^*+/+*^, *Rpl22*^*+/-*^ *and Rpl22*^*-/-*^ littermate mice had been backcrossed to the C57BL/6 background for ten generations and were maintained in the Association for Assessment and Accreditation of Laboratory Animal Care-accredited Laboratory Animal Facility at Fox Chase Cancer Center. Mice were housed and monitored weekly in addition to daily base monitoring by Fox Chase Cancer Center animal facility staff. Mice were euthanized by CO2 asphyxiation as per IACUC guidelines prior to isolation of tissues. For isolation of mouse embryonic fibroblasts, pregnant female mice (E14.5) were euthanized by C02 asphyxiation[2].

### Plasmids, cell lines, and viral production

Mouse *Rpl22L1* and *Rpl22* were cloned into the pQCXIP vector using NotI and AgeI restriction sites. The GFP fusion of RPL22 was constructed by cloning into the pACGFP-N1 vector, followed by transfer using the same restriction sites to pQCXIP for expression studies. pLKO.1-puro lentiviral shRNA constructs targeting murine and human *Rpl22L1* and *Rpl22* were purchased from Sigma-Aldrich. Human colon cancer cell lines HCT116, SW480, mouse immortalized colon epithelial cell line ModeK and the human normal colon epithelial cell line CCD 841 CoN obtained from Fox Chase Cancer Center Cell Culture Facility were purchased from American Type Culture Collection and maintained in DMEM medium with standard supplements 10% FBS (Hyclone). All cell lines have been validated by Short Tandem Repeat (STR) analysis. To ectopically express Rpl22L1 or GFP-RPL22, cells were infected with retrovirus or lentiviral produced by transient transfection of phoenix-amphitropic packaging cells. The infected (GFP+) cells were then isolated by flow cytometry using a FACSAria II (BD Biosciences). For shRNA knockdown experiments, lentivirus (pLKO.1-sh*Rpl22* or sh*Rpl22l1*) was produced by transfection of HEK293T with both packaging (delta8.2 and VSV-G) and pLKO.1 shRNA vectors using FuGENE 6 (Roche). Virus infected cells were puromycin selected for at least 5 days before experiments were performed. Sequences for shRNA targeting *Rpl22* and *Rpl22l1* are as follows:

1. mouse *Rpl22* shRNA1 (1026): ACCTGTAGAAGATGGAATCATG;
2. mouse *Rpl22* shRNA2 (1028): CCAGGAG AGAATCAAGGTGAA;
3. control shRNA (1092): TGGTTTGCATATGCATGAAGA;
4. human *RPL22L1* shRNA1: CCGGTTCTACGGGAGAAGGTTAAAGCTCGAGCTTTAACCTTCTCC-CGTAGAATTTTTG;
5. human *RPL22L1* shRNA2: CCGGGGACCCTTTCTCCCGAA-TAAACTCGAGTTTATTCGGGAGAAAGGGTCCTTTTTG;
6. human *RPL22* shRNA1: CCGGCGAATTACGTTACTTCCAGATCTCGAGATCTGGAAGTAACGTAATTCGTTTTTG;
7. human *RPL22* shRNA2: CCGGGTTCTGAAGTTCACTCTTGATCTCGAGATCAAGAGTGAACTTCAGAACTTTTTG.

### Patient derived xenograft (PDX) models, treatment, and other mouse studies

All xenografts were established at Experimental Pharmacology and Oncology Berlin-Buch GmbH (EPO) from primary patient material after the informed consent of the patients [15]. Colon PDX models were established as previously described [15]. Briefly, tissues removed from CRC patients were dissected into small fragments (about 3–5 mm) and were surgically implanted into the flanks of anaesthetized nude mice (NMRI: nu/nu, Taconic, DK). Once established, tumors were grown to a maximum size of 1 cm^3^ when they were routinely passaged. The in vivo growth rate of the xenotransplanted tumors was determined between passages 3 and 5. In order to retain as many features of the original tumor as possible, xenotransplanted tumors were only grown through 10 passages in mice. For further analysis tissue microarrays were constructed from formalin-fixed paraffin-embedded tissue at the Provitro AG, Berlin, Germany. A total of 19 PDX samples were used that were derived from 19 colon tumor samples from stage IIA primary colon cancer to metastasic colon cancer (S1 and S2 Tables). Most of the samples were primary tumor samples isolated before treatment and followed up for disease progression between 1999 to 2004[15]. Each PDX sample had 6 biological replicates in the array. For chemosensitivity profiling, tumor fragments were transplanted to mice (groups of 6–8 NMRI nude mice each). Mice were maintained under sterile and controlled environmental conditions. Mice with tumors (about 5mm X 5mm, mean volume 0.1cm^3^) were randomized either to receive treatment with 30mg/kg 5-FU (Ribofluor, Ribosepharm, Germany) or saline injected intraperitoneally once a day for five days. All drug doses and treatment schedules were optimized in previous studies[15]. All drugs were freshly prepared, as prescribed for clinical use, and used at an injection volume of 0.2 ml/20 g body weight. For all chemotherapy studies, therapeutic effects were assessed by twice weekly caliper measurements of the tumor. Tumor volumes (TV) were determined by the formula (width^2^ X length) X 0.5 and related to the value of the first treatment day (RTV, relative tumor volume). Treated to control (T/C) values of the RTV as a percentage were used for an evaluation of therapeutic efficacy. The following scores were used: negative = T/C>50%; + = T/C 36–50%; ++ = T/C 21–35%; +++ = T/C 6–20%; ++++ = T/C < = 5%[15]. These animal experiments were performed according to the regulations of the German Animal Protection Law and with the permission of the local responsible authorities. Mice were euthanized by CO2 asphyxiation at the end of the study and all efforts were made to minimize the suffering of the mice.

### Tumor microarray and immunohistochemistry (IHC), and immunoblot analysis

Colon cancer tissue microarrays (TMAs) were constructed by the FCCC Biosample Repository Facility according to an institutional review board approved protocol or at EPO Berlin Buch. All TMA sections were then paraffin embedded, sectioned, and subjected to IHC staining using rabbit anti-RPL22L1 antibody raised against the C-terminal 12 amino acids of Rpl22L1 or with rabbit IgG control. The RPL22L1 staining was viewed and scored by an experienced pathologist according to the intensity of the brown staining. The intensity score ranges from 0 to 3: 0 indicates lack of brown staining; 1 is weak staining; 2 is moderate/intermediate staining and 3 is strong/intensive staining. The cases with intensity score 3 fall into high expression group. The cases with intensity score 1–2 fall into low expression group. Rpl22L1 staining was also objectively quantified using the Vectra automated quantitative image system and presented as H-scores defined as the product of the area stained multiplied by the staining intensity[16]. Cut points distinguishing RPL22L1 low and RPL22 high staining in TMA from PDX were established using the H-Score and were verified by blinded analysis by a pathologist [17, 18]. All TMA were then scanned and evaluated by pathologist using light microscopy. For immunoblotting, NP-40 detergent extracts were resolved by SDS-PAGE and immunoblotted with primary antibodies reactive with the following proteins: 1) RPL22 [4]; 2) RPL22L1 (polyclonal rabbit serum raised against the C-terminal 12aa of human RPL22L1, as described[4]; 3) GAPDH (Abcam); 4) Tubulin (Sigma), followed by IRDye anti-mouse and anti-rabbit secondary antibodies (LI-COR) or regular anti-mouse and anti-Rabbit secondary antibody (Cell Signaling).

### Soft agar colony formation assay and proliferation assay

10,000–25,000 Mode K, HCT116, SW480, SW620, or HT29 colon cancer cells were mixed with an equal volume of 0.7% agar (Difco, BD Biosciences), plated on the top of 0.5% agar layer in 6-well plate in triplicate, and cultured for 2–3 weeks before crystal violate staining. A colony is defined as an aggregate of >40 cells. Colonies were counted under the inverted microscope at 4X and 10X objectives. For the EC50 and proliferation assay, cells were seeded with equal density in 96 well plates and treated with either vehicle control or 5-FU at different doses on day 2. After 48–72 hours, cells were then analyzed using cell proliferation reagent WST-1 (Sigma) according to the manufacturer’s instructions.

### Biostatistics and bioinformatics

The clinical outcomes data for 636 CRC cases were obtained from cBioportal cancer genome data resources (http://www.cbioportal.org/study?id=coadread_tcga#clinical). Overall survival (OS) was estimated using Kaplan-Meier methods. OS was based on OS status and months to death from initial pathologic diagnosis. For all animal studies, Kaplan-Meier curves of percent survival were analyzed with the Mantel-Cox log-rank test. Intervals between diagnosis and follow up ranged from 1.2 to 12years. The relationship between RPL22L1 staining and OS was assessed using two-sided log-rank tests. RPL22L1high expressors were defined by values in the top quartile for RPL22L1 staining. All tests were two sided with 5% type I error. For cell proliferation assays and other cell culture analysis, data were analyzed using a two-tailed paired Student’s *t-test*. For the PDX CRC model quantification of the Rpl22L1 staining, unpaired two-tailed Student’s *t-test* was used.

## Results

### *RPL22* loss is frequently observed in CRC and is associated with Rpl22L1 induction in human colorectal cancer

**Fig 1:**
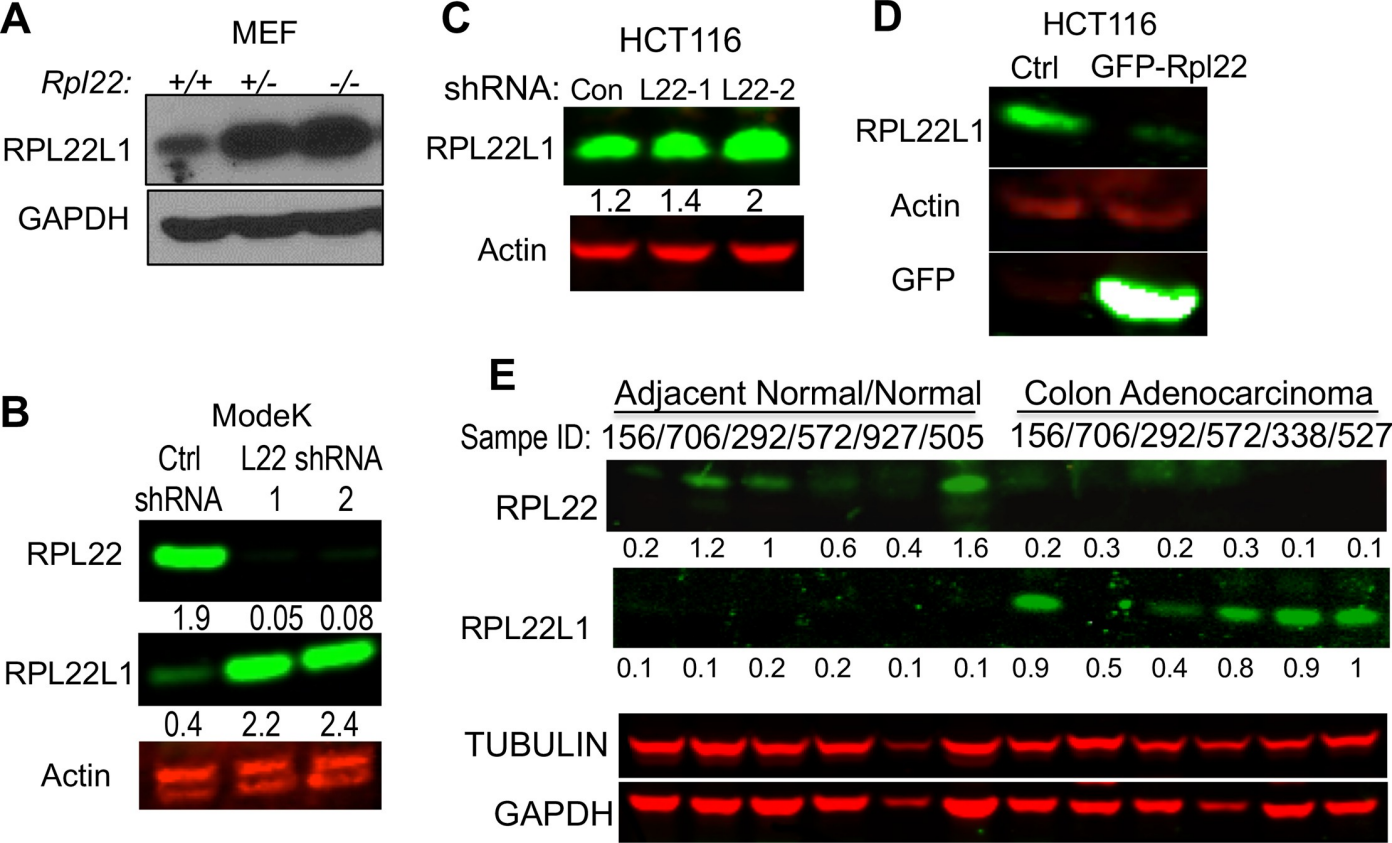
*RPL22* is frequently deleted in CRC and its loss results in induction of its highly homologous paralog, Rpl22L1. A. Immunoblotting of extracts of primary MEF from *Rpl22+/+*, *Rpl22+/-* and *Rpl22-/-* mice reveals that RPL22L1 expression is markedly induced by loss of RPL22. GAPDH served as loading control. B. Knockdown of *Rpl22* with two different shRNAs in the mouse colon epithelial line, ModeK, resulted in induction of RPL22L1 protein, as measured by immunoblotting. Actin served as loading control. C. Knockdown of RPL22 in the human colon cancer cell line HCT116, with two different shRNAs, results in induction of RPL22L1 protein, as measured by immunoblotting. Quantification of band intensity by ImageJ is listed on the bottom. Actin served as loading control. D. Overexpression of GFP-RPL22 inhibits RPL22L1 expression in HCT116 cells. Actin served as loading control. E. Immunoblotting of primary human colon adenocarcinoma samples reveals that RPL22L1 is more highly expressed in human colon adenocarcinoma samples compared with adjacent normal tissue. RPL22 expression is reduced in colon adenocarcinoma. Tubulin and GAPDH served as the loading controls for the RPL22 and RPL22L1 blots, respectively. RPL22 and RPL22L1 bands in B,C, and E were quantified by Image J program with background correction and normalized to gel loading control.

Previously we identified *RPL22* as tumor suppressor that was deleted or mutated in 10% of T-acute lymphoblastic leukemias. Interestingly, shallow deletion of *RPL22* (likely *Rpl22+/-*) was observed in 36% of CRC patients, which led us to explore its role in CRC (S1A Fig) (http://www.cbioportal.org/index.do?session_id=5a050a4c498e5df2e29800d3) [19]. RPL22 has a highly-homologous paralog, RPL22L1, that is 70% identical with RPL22, but whose function is unknown. To determine how *Rpl22* loss affected Rpl22L1 expression, we examined immortalized MEF cells with mono- (*Rpl22+/-*) or biallelic (*Rpl22-/-)* inactivation of *Rpl22* and found that *Rpl22* loss markedly increased the expression of RPL22L1 protein (Fig 1A) and RPL22L1 induction was not accompanied by a commensurate change in *Rpl22l1* mRNA, suggesting post-transcriptional regulation of expression (S1B Fig)[20]. The induction of RPL22L1 upon RPL22 loss appears to be a general phenomenon, as we found that Rpl22L1 expression was induced in most tissues from RPL22-null mice, specifically in colon (S1C Fig). The increase in RPL22L1 expression is causally linked to RPL22 loss, because knockdown of *Rpl22* in the mouse intestinal epithelial cell line ModeK (Fig 1B) and the CRC cell line HCT116 (Fig 1C), using two different hairpins, was sufficient to substantially increase RPL22L1 expression, suggesting that RPL22 negatively regulates Rpl22L1. Interestingly, ectopic expression of RPL22 in HCT116 human colon cancer cells, which express higher levels of RPL22L1 than normal colon epithelial cells (S1D Fig), repressed RPL22L1 expression (Fig 1D). Because *RPL22* was found to be deleted frequently in CRC patients, we asked whether RPL22L1 expression was also elevated in primary human colon adenocarcinoma patient samples. Indeed, RPL22L1 expression was frequently elevated in colon adenocarcinomas relative to adjacent normal and normal colon tissue, whereas RPL22 was preferentially expressed in normal colon samples (Fig 1E). Similar results were observed in colon cancer cell lines (S1E Fig). Thus, RPL22 negatively regulates the expression of RPL22L1, suggesting that RPL22L1 induction has the potential to serve as a surrogate marker for *RPL22* inactivation in human CRC.

### Rpl22L1 is a proto-oncogene that promotes cell proliferation and anchorage-independent growth

**Fig 2:**
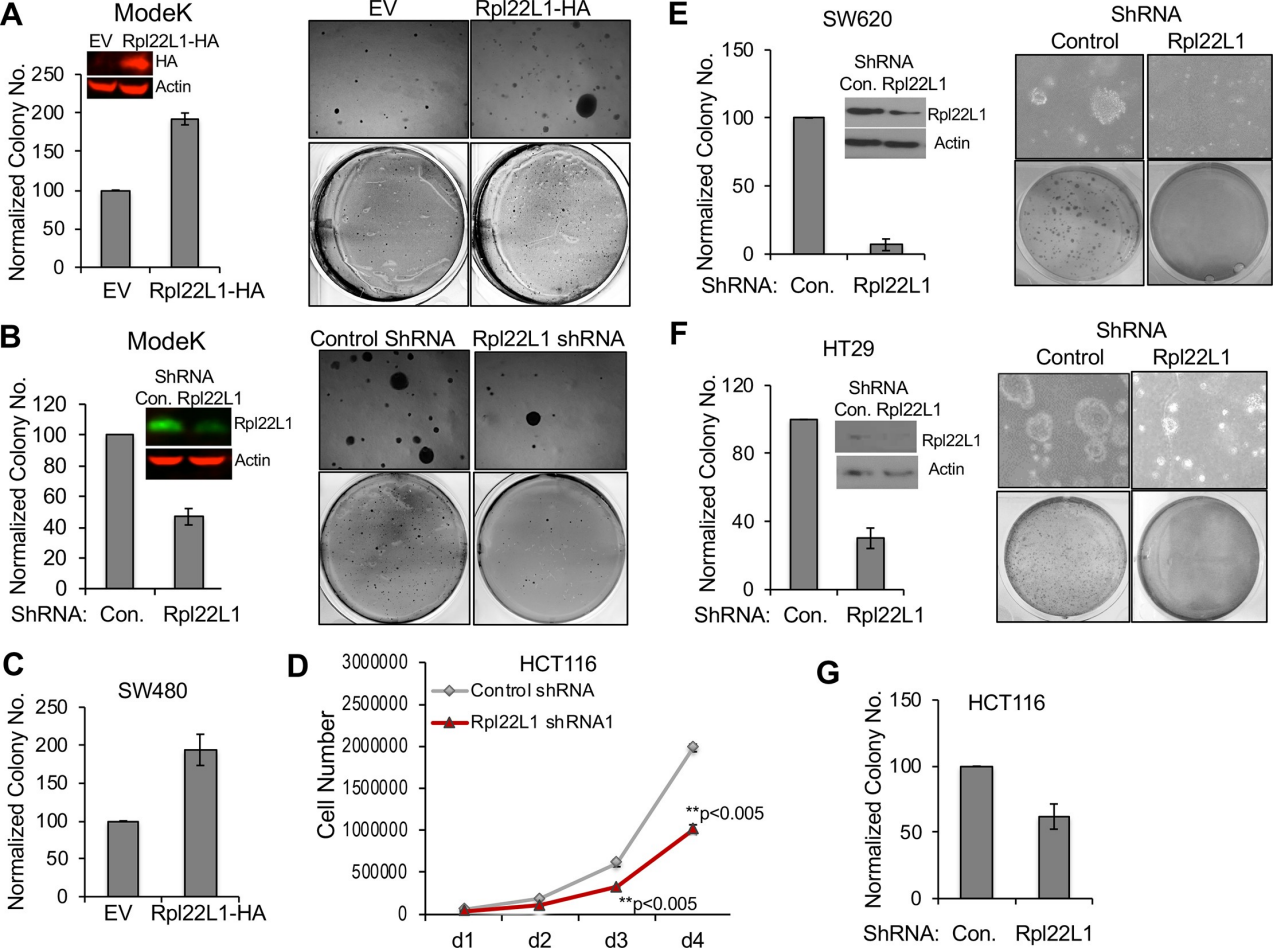
Rpl22L1 promotes growth and anchorage-independence. A. Overexpression of epitope-tagged RPL22L1 (RPL22L1-HA) in ModeK colon epithelial cells promotes anchorage-independent cell growth, as evidenced by colony formation. Average of colony numbers counted in each experiment were plotted as “% of control” shown on the left with representative images displayed on the right. B. Knockdown of *Rpl22l1* in ModeK cells inhibits anchorage-independent cell growth. Quantification of colony numbers is displayed graphically on the left and representative image on the right. C. Overexpression of RPL22L1 (RPL22L1-HA) in SW480 colon cancer cells promotes anchorage-independent cell growth as measured by colony formation. Data are displayed as in A. D. Knockdown of *Rpl22L1* significantly reduces HCT116 cell proliferation. HCT116 cells were infected with either control shRNA or shRNA targeting *Rpl22L1*. Cell numbers were counted each day for four days and triplicate measures were depicted graphically. Data are plotted as the average cell number ± SD from each day. Student’s t test was used for biostatistical analysis between groups, **, p<0.005. E-F. Colon cancer cells SW620 (E) and HT29 (F) were transduced with either control shRNA or *Rpl22L1* shRNA and plated in soft agar to assess the effect on colony formation after 2 weeks. Quantification of colony numbers is depicted graphically on the left and a representative picture is displayed on the right. G. Knockdown of *Rpl22L1* in HCT116 cells significantly decreased colon formation as evidenced following two weeks of growth in soft agar. Data in A, B, C, E, F, G depicted as “% control± SEM” as a summary from 3 independent experiments performed in triplicate. Student’s *t test* was used for biostatistical analysis between groups, *, p<0.05, **p<0.05.

We previously determined that RPL22 loss increased both cell proliferation and anchorage-independent growth [2]. Given that RPL22 loss leads to RPL22L1 overexpression, we wished to determine if RPL22 loss promoted these behaviors through RPL22L1 induction. Indeed, we observed that overexpression of epitope-tagged RPL22L1 in ModeK intestinal epithelial cells led to increased anchorage-independent growth in soft agar (Fig 2A), whereas knockdown of RPL22L1 in ModeK cells inhibited anchorage-independent cell growth (Fig 2B). To further determine if RPL22L1 induction was sufficient to promote these behaviors in colon cancer cell lines, we overexpressed RPL22L1 in colon cancer cell line SW480 and found that Rpl22L1 induction was sufficient to promote growth (S2A Fig) and anchorage independence (Fig 2C). Likewise, shRNA knockdown of RPL22L1 decreased proliferation of colon cancer lines HCT116 and HT29 (Fig 2D and S2B and S2C Fig), and the anchorage-independent cell growth of SW620, HT29 and HCT116 (Fig 2E–2G). Together, these data suggest that RPL22L1 induction is capable of promoting the increased growth and anchorage independence displayed by cells in which *Rpl22* has been lost or inactivated. The effect of Rpl22L1 on anchorage independent growth is not restricted to colon cancer cell lines. Overexpression of RPL22L1 in RPL22-expressing wild type, immortalized MEF increased their proliferation relative to control transfected cells (S2D Fig). Moreover, ectopic expression of RPL22L1 was also able to promote anchorage-independent growth of Ras-transformed MEF cells, conferring on them the same behavior exhibited by RPL22-deficient MEF (S2D Fig).

### Rpl22L1 is highly expressed in colon adenocarcinoma and correlates with poor prognosis

**Fig 3:**
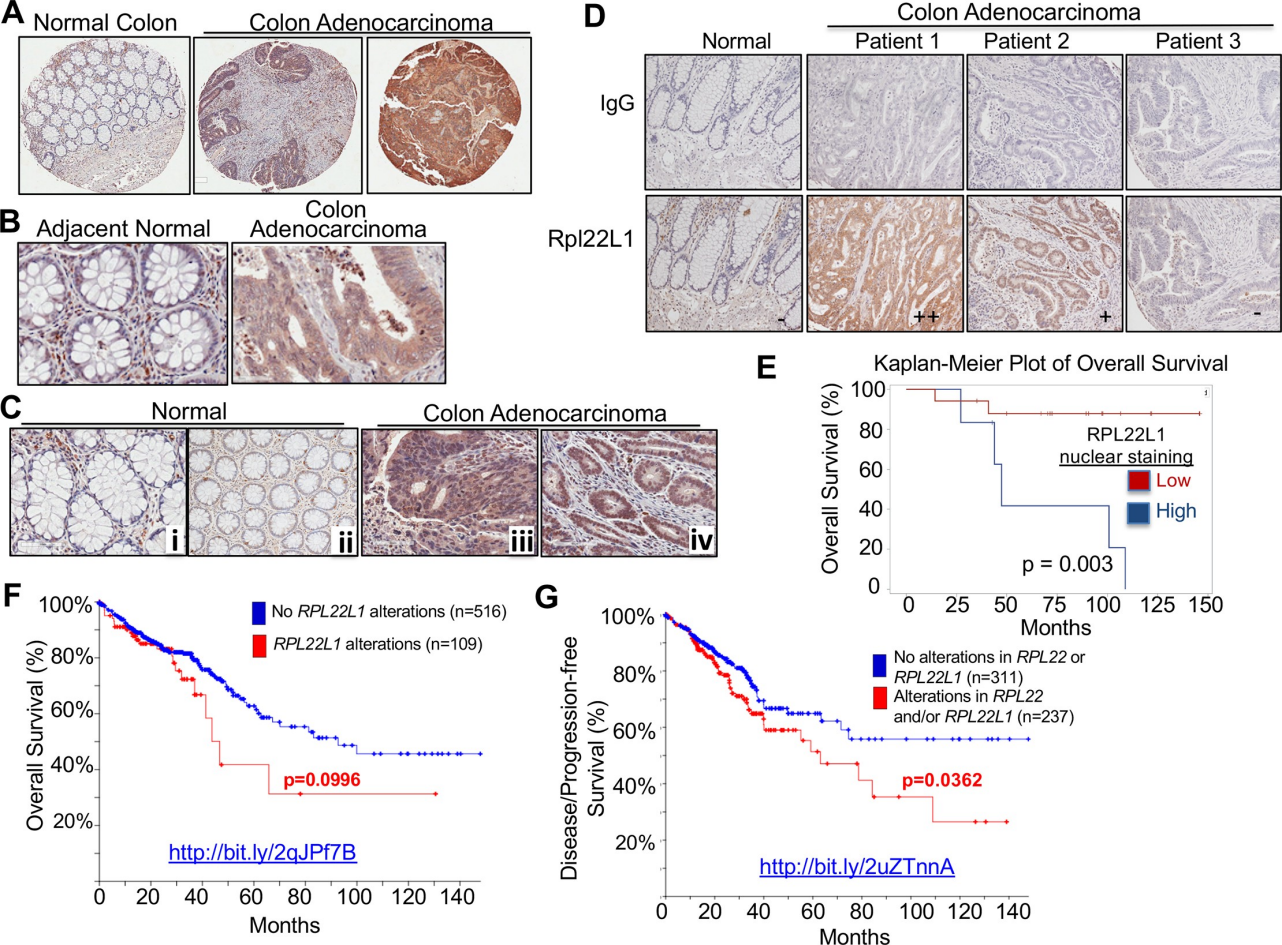
High expression of Rpl22L1 is correlated with poor survival in CRC. A-B. Representative pictures from a CRC tissue microarray (TMA) show that RPL22L1 is highly expressed in colon adenocarcinoma compared with normal colon at 4X magnification (A) and 20X magnification (B). C. RPL22L1 staining is increased in the cytoplasm (iii) and nucleus (iv) of colon adenocarcinoma compared with normal controls (i-ii), which mainly exhibit stromal staining. D. RPL22L1 expression is variable in different colon adenocarcinoma samples (“-“, negative; “+”, weak positive; and “++”, strong positive). IgG serves as antibody negative control. E-G. Kaplan-Meier analysis of Overall Survival in 23 colon cancer patients using Rpl22L1 IHC data from TMA analysis (OS: E, p = 0.003) or genomic alterations in 625 colon cancer patients from the TCGA provisional data set (OS; F, p = 0.0955). Kaplan-Meier analysis of Disease/Progression-free Survival (G, p = 0.0363) revealed that patients with *Rpl22L1* alterations (copy number gain and two-fold greater upregulation) and/or alterations in *RPL22* have reduced survival when compared with patients without those alterations. Web links to the TCGA data are embedded in the figure panels.

To further explore the extent of RPL22L1 induction in human colon cancer patients and assess its relationship to outcomes, we performed RPL22L1 immunohistochemistry (IHC) on a multi-tumor and colon cancer tissue microarrays (TMA). Because no commercial antibodies against RPL22 or RPL22L1 gave reliable IHC results in patient tumor samples, we developed an anti-human RPL22L1 polyclonal antibody and verified its specificity and its function in immunoblotting, immunofluorescent staining, and immunohistochemistry (S3 Fig). IHC analysis was performed on multi-tumor TMAs (S4 Fig), which revealed that RPL22L1 is highly expressed in a variety of solid tumors including renal cell carcinoma, breast cancer, lung cancer as well as colon sarcoma and colorectal carcinoma (Fig 3A–3C and S4 Fig). Anti-RPL22L1 was found to stain both the nucleus and cytoplasm (Fig 3C). The intensity of RPL22L1 staining was variable (Fig 3D) and elevated RPL22L1 staining was found to be significantly associated with colon adenocarcinoma (Table 1, n = 23). Importantly, the patients with the highest level of RPL22L1 staining in this small cohort, exhibited reduced survival. Log Rank analysis following VECTRA quantitation of RPL22L1 staining revealed that the association with elevated RPL22L1 staining and reduced survival was significant, particularly for nuclear RPL22L1 staining (p = 0.003; Fig 3E and S3 Table). Moreover, while elevated RPL22L1 expression was not associated with tumor size, it was associated with increased lymph node involvement (S5 Fig). We also found that 2 of the 4 deceased patients with high cytoplasmic RPL22L1 staining failed 5-FU-based chemotherapy, raising the possibility of an association of RPL22L1 induction with poor prognosis and chemoresistance. Consistent with findings in our cohort, further analysis of colon cancer patient data in TCGA (http://bit.ly/2qJPf7B) using CBioportal revealed a gain in *Rpl22L1* copy number in 18% of patient samples (n = 636, *Adenocarcinoma*,*TCGA*, *Provisional*; Fig 3F), which when combined with heterozygous loss/mutation of *RPL22* (http://bit.ly/2uZTnnA; Fig 3G), was significantly associated with reduced disease/progression-free survival (p = 0.0362)[19]. It is important to note that the induction of RPL22L1 protein that occurs upon Rpl22 loss is primarily post-transcriptional (Fig 1A and S1B Fig). Consequently, alterations in *RPL22L1* copy number or mRNA level are likely to have less prognostic power than analyzing RPL22L1 protein. Taken together, these data indicate that high RPL22L1 protein expression has the potential to serve as a marker of poor prognosis in CRC.

**Table 1:** IHC staining for RPL22L1 in human colon adenocarcinoma patients.

Rpl22L1 IHC	Negative	Positive	Total
Normal/Adjacent Normal	17	2	19
Colon Adenocarcinoma	8	15**	23
Total	25	17	42

** Fisher’s exact test P = 0.0004

### Rpl22L1 is highly expressed in 5-FU resistant PDX colon tumors

**Fig 4:**
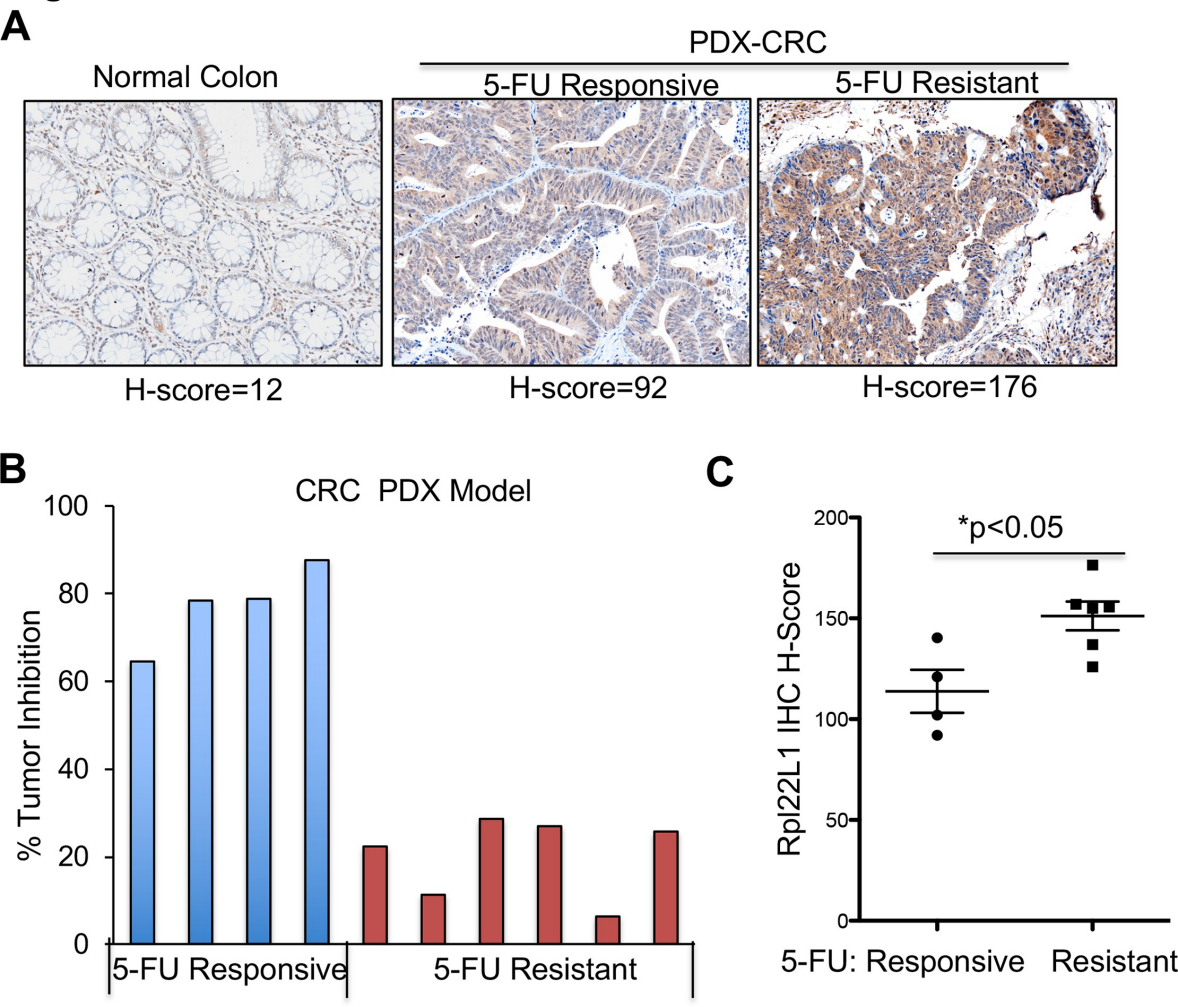
5-FU resistant CRC-PDX samples exhibit increased Rpl22L1 staining. A. Representative picture from a TMA constructed using CRC PDX samples reveals that RPL22L1 is highly expressed in CRC PDX samples compared with normal colon (20X magnification). Staining was quantified by VECTRA automated quantitative image system and presented as average of H-Score. B. Percentage of tumor inhibition after 5-FU treatment in CRC PDX. Tumor size reduction of more than 64% is defined as 5-FU responsive (n = 4) whereas tumor inhibition less than 30% is 5-FU resistant (n = 6). C. RPL22L1 is more highly expressed in 5-FU resistant CRC compared with 5-FU responsive CRC in B (Student’s t test, p<0.05).

Given the association of elevated RPL22L1 expression and poor outcomes, we wished to explore the mechanistic link. In doing so, we focused on chemoresistance, since 5-FU-based chemotherapy remains a mainstay in the treatment of CRC and 2 of the 4 deceased RPL22L1 high patients in our cohort failed 5-FU based therapy. To assess a potential role for RPL22L1 in resistance to 5-FU based therapy, we performed an avatar trial in mice using CRC patient-derived-xenograft (PDX) samples (S1 and S2 Tables). CRC tumor specimens from different patients were transplanted into nude mice, which were then treated with 5-FU or vehicle control and examined for tumor growth and RPL22L1 expression. Tumor samples were harvested from mice and used to construct TMAs, which were stained with anti-RPL22L1. Upon quantification of RPL22L1 staining and generation of H-scores (S3 and S6A Figs), we found that while RPL22L1 expression was variable in the transplanted CRC samples, it was nevertheless higher than in normal colon (Fig 4A). 5-FU responsiveness was quantified by determining the percentage decrease in tumor size following 5-FU treatment. The PDX samples were assembled into 5-FU sensitive (Tumor inhibition >64%) and 5-FU resistant (Tumor inhibition <30%) groups, which revealed that RPL22L1 expression was significantly higher in the 5-FU resistant group (p<0.05; Fig 4B and 4C; S1 and S2 Tables). Taken together, these data indicate that elevated RPL22L1 expression is associated with 5-FU resistance.

### Rpl22L1 overexpression confers 5-FU resistance

**Fig 5:**
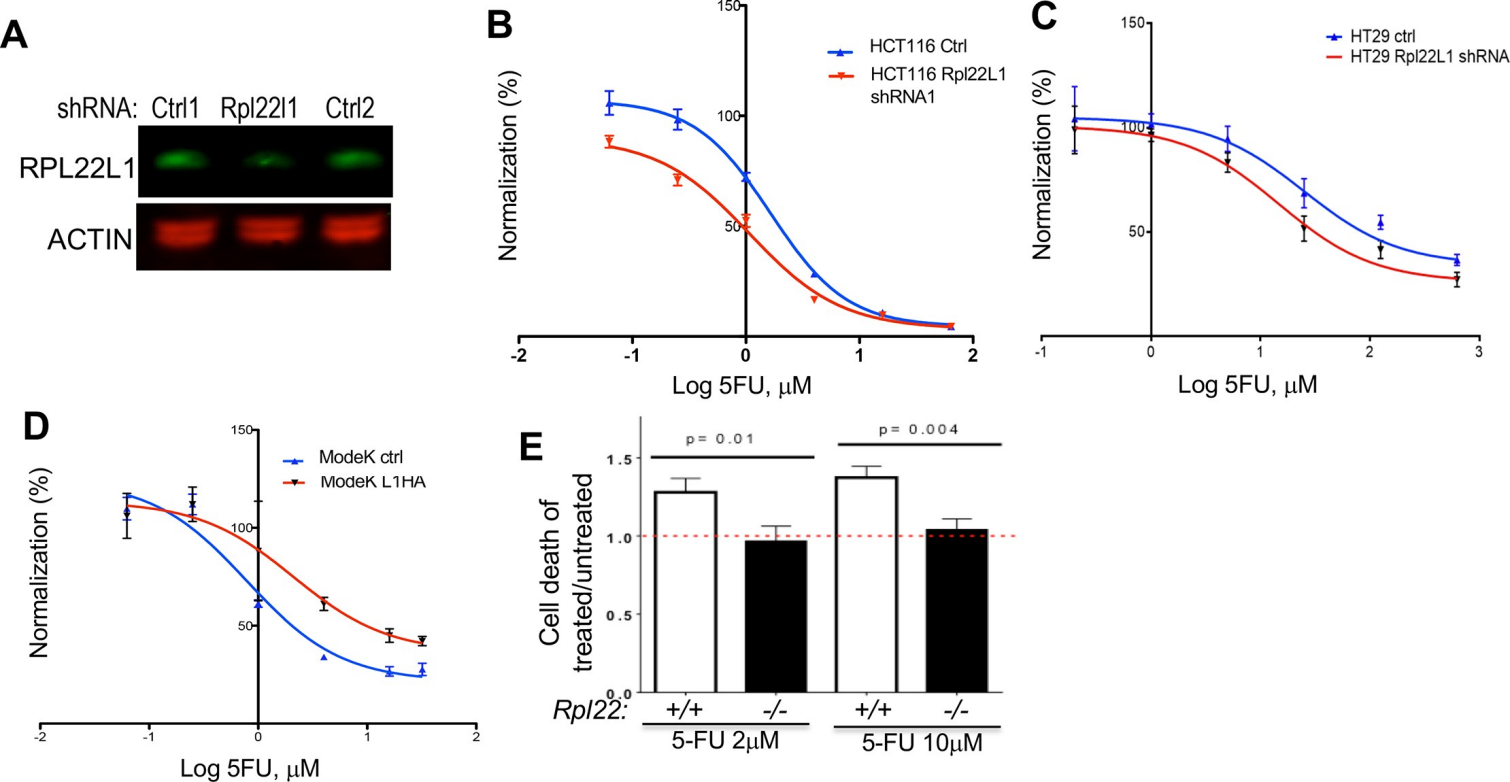
Rpl22L1 regulates 5-FU responsiveness in CRC. A. Immunoblot analysis reveals the efficacy of RPL22L1 knockdown in HCT116 cells. Actin served as loading control. B-D. Dose-response analysis reveals that knockdown of *Rpl22L1* in HCT116 cells (B) and HT29 cells (C) increases the sensitivity to 5-FU treatment compared with cells expressing a control shRNA. Viability was measured by WST assay. HCT116 and HT29 cells were transduced with control shRNAs or *Rpl22L1* shRNA and treated with indicated concentration of 5-FU for 48 hours. D. Dose-response analysis reveals that overexpression of RPL22L1 (L1HA) in ModeK cells increases resistance to 5-FU treatment compared with cells transduced with control vector. Effects on cell viability were assessed by WST assay on cells transduced with RPL22L1-HA (L1HA) or vector control and treated with indicated concentration of 5-FU for 48 hours. Results depicted in B-D are representative of three independent experiments and represent the percentage of response inhibition vs log range of 5-FU dose (uM). E. Hematopoietic progenitors from RPL22-deficient mice, which express high RPL22L1 levels, are more resistant to death induced by two different doses of 5-FU, than those from RPL22-sufficient mice.

The association between elevated RPL22L1 levels and 5-FU resistance could either result from RPL22L1 induction by 5-FU treatment or the capacity of RPL22L1 to influence 5-FU responsiveness. To distinguish these possibilities, we assessed the effect of prolonged 5-FU treatment (3 months) on RPL22L1 expression by ModeK and HCT116 cells. No increase in RPL22L1 expression was observed (S6B Fig), raising the possibility that the expression level of RPL22L1 was influencing 5-FU responsiveness. To determine whether RPL22L1 could regulate 5-FU responsiveness, we knocked RPL22L1 down in HCT116 cells and assessed the effect on 5-FU responsiveness. Indeed, RPL22L1 knockdown decreased the IC50 of 5-FU for HCT116 cells by 2.2-fold (2.84uM±0.99vs 1.28uM±0.33) (Fig 5A and 5B and Table 2), indicating an increased sensitivity to 5-FU treatment. Similar results were observed in HT29 colon cancer cell lines (Fig 5C). Likewise, overexpression of Rpl22L1 in ModeK cells (Fig 5D) led to an increase in 5-FU resistance as indicated by an increase in the IC50 for 5-FU (Table 2). We also tested the effect of manipulating Rpl22L1 expression on 5-FU responsiveness in SW620 cells and SW480 cells. We observed consistent 2-fold changes in IC50 across all cell lines tested (Table 2). The increase in 5-FU resistance upon induction of RPL22L1 is not restricted to normal or transformed colon cells, as hematopoietic progenitors from RPL22 null mice, which express high levels of RPL22L1, also display resistance to 5-FU induced cell death *in vivo* (Fig 5E).

**Table 2:** Effect of altering RP22L1 expression of 5-FU responsiveness.

Cell lines	Control shRNA	*RPL22L1* shRNA	Control vector	*RPL22L1*-HA
HCT116(n = 3)	2.84±0.99	1.28±0.33		
HT29(n = 3)	22.94±3.87	9.44±2.67		
SW620(n = 2)	20.45±5.45	12.9±2.9		
ModeK(n = 3)			1.03±0.24	3.06±0.92
SW480(n = 3)			1.34±0.36	2.89±0.36

IC50 values (μM) ± SEM of 5-FU calculated from results of 2–3 independent experiments in Fig 5B–5F.

### Rpl22L1 promotion of 5-FU resistance is associated with alterations in MGMT and MLH1 expression

**Fig 6:**
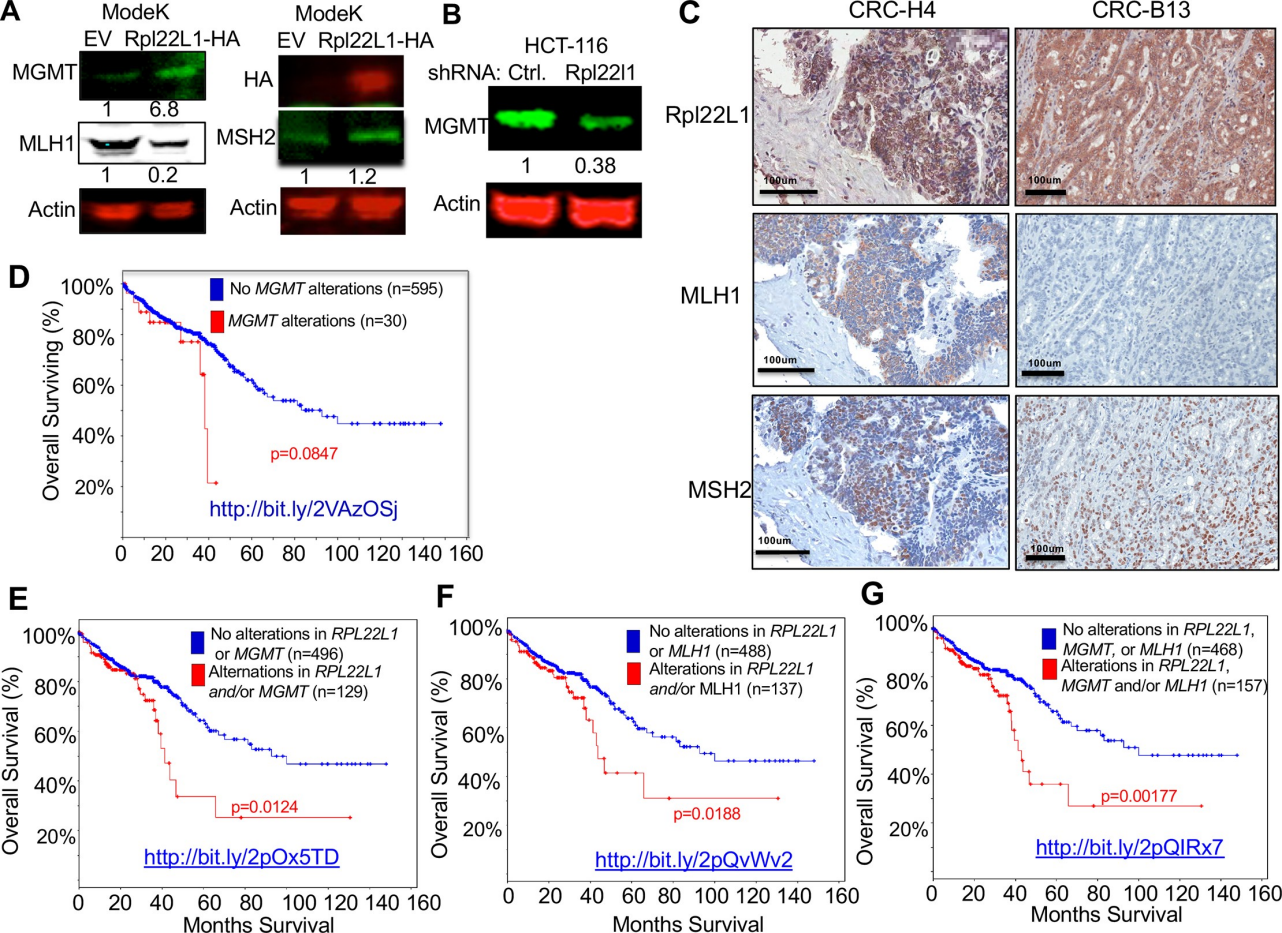
Rpl22L1 regulates the expression of DNA damage repair enzymes, MGMT and MLH1, which together with Rpl22L1 induction, contribute to poor prognosis. A. Immunoblotting performed on extracts from Mode K cells stably expressing control or RPL22L1-HA reveals increased expression of MGMT, decreased expression of MLH1, and no effect on MSH2. Actin served as loading control. B. Knockdown of RPL22L1 in the HCT116 cell line leads to decreased expression of MGMT. Actin served as loading control. C. Representative images of IHC staining for RPL22L1, MLH1, and MSH2 for two RPL22L1 high CRC samples from our TMA. CRC-B13 lacks MLH1 expression and so is considered MSI, while CRC-H4 expresses both MLH1 and MSH2 and is considered MSS. Image magnification is indicated in the scale bar. D-F. Kaplan-Meier overall survival curves for patients containing or lacking alterations in (D) *MGMT* alone (amplification, copy number gain and mRNA upregulation; (E) *Rpl22L1* or *MGMT* (amplification, copy number gain and upregulation; (F) *Rpl22L1* (amplification, copy number gain and mRNA upregulation) or *MLH1* (mutation and two-fold downregulation); (G) alterations in *Rpl22L1*, *MGMT*, or *MLH1*. These combinations of alterations are associated with poor overall survival in the colorectal adenocarcinoma TCGA provisional data relative to patients lacking them. Web links to the TCGA data are embedded in the figure panels.

We next wished to gain insight into the mechanism by which changes in RPL22L1 expression influence 5-FU sensitivity. 5-FU resistance in CRC has long been linked to inactivation of mismatch repair (MMR) genes, such as MLH1 [21–23]. Likewise, overexpression of the DNA repair enzyme O6-methyguanine-DNA-methyltransferase (MGMT) has also been associated with drug resistance [24, 25]. Consequently, we asked if RPL22L1 overexpression altered the expression of MLH1, MSH2, or MGMT. Indeed, RPL22L1 overexpression increased the expression of MGMT and decreased the expression of MLH1, but had no effect on MSH2 (Fig 6A). Conversely, RPL22L1 knockdown reduced MGMT expression (Fig 6B). These data suggest that RPL22L1 may be influencing 5-FU responsiveness by modulating the expression of genes involved in MMR or through modulation of MGMT, which is involved in repair of DNA-damage caused by chemotherapy agents[26, 27]. Importantly, elevated RPL22L1 is not simply a reflection of the microsatellite instability (MSI) status of the CRC tumors in our study, as 3 of the 5 RPL22L1 high CRC samples in our cohort were determined to be microsatellite stable (MSS) by IHC for MMR genes. Examples of MSS (CRC-H4) and MSI (CRC-B13) RPL22L1 high CRC samples are depicted in Fig 6C. Moreover, 6 of 7 of the RPL22L1 high 5-FU resistant CRC PDX samples in our avatar trial were also determined to be MSS based on expression of MMR genes (S7 Fig). The association between RPL22L1 induction and 5-FU resistance may be mediated through effects on MLH1 and MGMT expression.

Having found that RPL22L1 is able to regulate the expression of genes that contribute to 5-FU resistance (i.e., MLH1 and MGMT), we wished to determine if linking changes in those genes to *RPL22L1* amplification strengthened its prognostic value. Indeed, patients with either *RPL22L1* copy number gain or *MGMT* induction (upregulation & amplification) exhibited significantly reduced overall survival (OS) relative to patients with MGMT induction alone (Fig 6D, p = 0.0847) or those lacking alterations in *MGMT* or *RPL22L1* (Fig 6E, p = 0.0124). Likewise, patients with *RPL22L1* copy number gain or *MLH1* loss (mutation & downregulation) also exhibited reduced OS (Fig 6F, p = 0.0188). Finally, the association of *RPL22L1* amplification with reduced OS was even more significant when combined with increased *MGMT* or reduced *MLH1* (Fig 6G, p = 0.00177). Thus, our data suggest that RPL22L1 induction contributes to CRC pathogenesis and treatment resistance and this is associated with alterations in MGMT and MLH1 expression that may contribute to 5-FU resistance.

## Discussion

We have previously reported that RPL22 functions as a tumor suppressor in T-acute lymphoblastic leukemia [2, 5]. We now report that *RPL22* loss also occurs in solid cancers, including CRC, where the *RPL22* locus is deleted in ~36% or CRC patients. RPL22 loss results in the induction of its paralog, RPL22L1, because RPL22 normally functions to repress RPL22L1 expression [20]. The induction of RPL22L1 upon RPL22 loss has three critical implications. First, RPL22L1 appears to function as a critical molecular effector through which RPL22 loss increases cellular transformation potential, as evidenced by increased proliferation and anchorage independence. Specifically, RPL22L1 induction is sufficient for these behaviors, suggesting that RPL22L1 is functioning like a cellular proto-oncogene in CRC. Second, because RPL22 directly regulates RPL22L1 expression, at least in some circumstances, RPL22L1 induction may serve as a surrogate for RPL22 loss. Finally, RPL22L1 induction is associated with reduced survival in CRC, suggesting that it may have prognostic value. Our data also suggest that the association between RPL22L1 induction and poor prognosis may relate to alterations in sensitivity to 5-FU, because RPL22L1 overexpression in colon epithelial cells and in CRC cell lines increases 5-FU resistance. The increased 5-FU resistance exhibited upon RPL22L1 overexpression is associated with alterations in the expression of DNA repair proteins MGMT and MLH1, which have previously been implicated in 5-FU resistance[21, 22].

Our data reveal a link between poor colorectal cancer prognosis and RPL22L1 overexpression. This is of great importance because CRC incidence in increasing worldwide, and so prognostic biomarkers that predict outcomes and can be used to guide therapy are needed. This is particularly true in later stage CRC, where surgery is not applicable and chemotherapy is ineffective [12–14]. While RP are frequently mutated in human cancers, this is the first report of induction of an RP potentially serving as a prognostic marker. Indeed, our limited study of RPL22L1 protein levels and outcomes in CRC revealed that in our small cohort, patients with CRC expressing high levels of RPL22L1 exhibit a significant reduction in survival. The link between RPL22L1 induction and reduced survival is further supported by meta-analysis of the large CRC patient cohort in TCGA, which also revealed a link between *RPL22L1* induction and poor outcomes for CRC patients. Patients with *RPL22L1* copy number gain trended toward reduced survival; however, the reduction in survival was not statistically significant when considered in isolation. We think this is likely because RPL22L1 expression is primarily controlled post-transcriptionally and so the prognostic value of RPL22L1 itself is best assessed by measuring RPL22L1 protein levels or in combination with *RPL22* loss, which induces RPL22L1 protein expression. In agreement, patients with *RPL22L1* copy number gain combined with *RPL22* loss display significantly reduced disease-free survival. Moreover, when *RPL22L1* induction is considered together with alterations in DNA repair enzymes (*MGMT* and *MLH1*) modulated by RPL22L1, its prognostic value becomes even clearer, as CRC patients exhibiting these alterations exhibit significantly reduced OS. We think this is likely because the modulation of these proteins (MGMT and MLH1) by RPL22L1 alters the repair of DNA damage induced by 5-FU based chemotherapeutic treatments, rendering those patients’ CRC more resistant. Studies are ongoing to determine whether RPL22L1 can function as a driver in CRC beyond the effects we have observed in vitro. It is important to re-emphasize that RPL22L1 is primarily regulated post-transcriptionally and so the relationship between its induction and patient outcomes is best assessed by measuring protein levels. Consistent with this perspective, our study of RPL22L1 protein levels and outcomes revealed an association with poor outcomes in CRC. Nevertheless, these analyses are based on a relatively small sample size and so further validation on a large, independent CRC cohort will be required to fully assess the prognostic potential of RPL22L1 in CRC.

An important remaining question is whether, in addition to serving as a prognostic marker for aggressive CRC, RPL22L1 induction might also play a role in influencing CRC disease course. Our in vitro data suggests that RPL22L1 induction might influence disease course in two ways. First, RPL22L1 induction might promote the aggressiveness of CRC, since ectopic expression of RPL22L1 promotes both proliferation and anchorage independent growth of immortalized colon epithelial cells and CRC cell lines. Likewise, RPL22L1 induction could also affect response to treatment. Indeed, 5-FU based chemotherapy is a mainstay of CRC treatment [14, 28–30] and ectopic expression of RPL22L1 enhances 5-FU resistance, which is associated with both increased MGMT expression and decreased expression of the MMR gene, MLH1. Consequently, RPL22L1 induction may not only predict 5-FU resistance, but may also actively promote that resistance to 5-FU based therapy. It is important to note that there is a well-documented link between defects in MMR genes and CRC resistance to adjuvant 5-FU-based chemotherapy; however, the causal link of defective MMR with drug resistance or poor prognosis remains unclear [21–23, 31, 32]. Specifically, while some CRC patients with MMR-defects and considered to be MSI high do not seem to derive benefit from adjuvant 5-FU-based chemotherapy, they actually exhibit increased survival, rather than the decreased survival observed for RPL22L1 high patients [23, 32–34]. Here we showed that MSI status is unlikely to be the explanation for the 5-FU resistance of RPL22L1 high patients in our cohort or in our avatar trial. We have investigated the MSI status of the RPL22L1 high patients and PDX samples that we analyzed and found that the vast majority of our samples were MSS, as evidenced by normal expression levels of MMR proteins (S7 Fig). Nevertheless, in some of the RPL22L1 high CRC patients samples analyzed, there was subclonal loss of either MLH1 or MSH2; however, a recent study linking expression of MMR genes with MSI status reported that unless loss of the MMR protein occurred in more than 75% of the tumor cells, the tumors appear to be MSS [35]. Based on these findings, 3 out of 5 RPL22L1 high CRC patient samples and 6 of 7 RPL22L1 high CRC PDX were categorized as MSS. Thus, together these data suggest that RPL22L1 induction downregulates the expression of MLH1, but not sufficiently to produce an MSI phenotype. Moreover, combining RPL22L1 induction with defects in the DNA damage repair factors it regulates (MGMT and MLH1), further increases the significance of its capacity to prognosticate poor outcomes, including reduced OS. Epigenetic modification of MGMT expression is frequently observed in CRC [36, 37] [38], but neither MGMT promoter hypermethylation or loss of MGMT alone serves as prognostic marker of CRC [39]. Instead, its overexpression is linked with drug resistance. In glioblastoma multiforme patients, high MGMT expression is associated with temozolomide resistance, while tumor cells lacking MGMT activity are more sensitive to alkylating agents [40]. This raises the possibility that the 5-FU resistance of RPL22L1 high CRC might be abrogated by treatment with inhibitors of MGMT, such as benzylguanine [26]. It remains unclear how RPL22L1 regulates MGMT and MLH1. RPL22L1 is dispensable for both ribosome biogenesis and global protein synthesis [3], but is able to selectively bind mRNA species bearing its consensus binding motif and regulate their expression [3, 20, 41]. For example, during early embryogenesis, RPL22L1 regulates the pre-mRNA splicing of human MLH1, raising the possibility that this could be occurring in CRC [41].

Taken together, these results reveal that the RP, RPL22L1, is capable of promoting CRC growth and anchorage independence, as well as therapeutic resistance. Consequently, gaining insight into its mechanism of action and the cellular pathways it regulates may enable the identification of therapeutic vulnerabilities in CRC patients with RPL22 loss and/or RPL22L1 induction.

## Supporting information

S1 Fig *RPL22* is frequently deleted in CRC and its loss results in Rpl22L1 induction. A. *RPL22* shallow deletion occurs in up to 36% of CRC samples. Data were collected from TCGA colorectal adenocarcinoma studies n = 223. B. Representative qRT-PCR analysis of *Rpl22* and *Rpl22l1* mRNA performed in triplicate is plotted graphically as the mean ± SD. *Rpl22* and *Rpl22l1* mRNA expression was normalized to *Gapdh* mRNA levels in MEF cells isolated from *Rpl22+/+*, *Rpl22+/-* and *Rpl22-/-* mice. C. Immunoblotting of extracts of tissues collected from *Rpl22+/+* and *Rpl22-/-* mice reveals that RPL22L1 expression is markedly induced by loss of *Rpl22*. Elevated expression of RPL22L1 was observed in normal colon. D. Immunoblotting of the human CRC cell line, HCT116, reveals that is expresses higher levels of RPL22L1 than the normal colon epithelial cell line CCD841 (Control). E. Immunoblotting of normal colon epithelial cell lines and human CRC cell lines reveals that colon cancer cells express higher levels of RPL22L1 and lower levels of RPL22 relative to normal colon epithelial cell lines. GAPDH served as loading control. Quantification of band intensity by ImageJ is listed on the bottom.
(TIF)

S2 Fig RPL22L1 promotes cell proliferation and colony formation. A-B. Overexpression of RPL22L1 (RPL22L1-HA) in SW480 colon cancer cells promotes cell proliferation (A) and knockdown of Rpl22L1 in HT29 colon cancer cells inhibits cell proliferation (B). Data is depicted as average of cell number ± SD from triplicate measurements every two days. Student’s t-test was used for biostatistical analysis between groups (*, p<0.05). C. Knockdown of RPL22L1 with two different shRNA (left) inhibits cell proliferation (right) of HCT116 cells. D. Overexpression of Rpl22L1 in immortalized or Ras-transformed MEF cells promotes cell proliferation as determined by cell numbers (left) and colony formation (right). Student’s t-test was used for statistical analysis between groups. **, p<0.005.
(TIF)

S3 Fig Validation of the anti-RPL22L1 antibody in immunoblotting, immunofluorescence, and immunohistochemistry (IHC). A. Immunoblot analysis reveals that knockdown of RPL22L1 by two different shRNA hairpins in HEK293 cells eliminates immunoreactivity. GAPDH served as loading control. B. Immunofluorescent staining with anti-RPL22L1 followed by FITC anti-rabbit secondary antibody, reveals that RPL22L1 knockdown abrogates staining. C. Knockdown of RPL22L1 in HEK293 cells attenuates staining with anti-RPL22L1 in IHC. D. IHC staining with anti-RPL22L1 of spleens from WT, Rpl22l1-/-, and Rpl22-/- mice. Images at 20x magnification are shown, which validate the specificity of the antibody. E. IHC with anti-RPL22L1 in human normal colon or colon adenocarcinoma samples. IgG in normal colon tissue is used as the negative control. F. Representative image of strong (left) and weak (right) staining of human colon cancer by anti-RPL22L1 in IHC. The staining was quantified by VECTRA automated quantitative image system and presented with H-Score.
(TIF)

S4 Fig Map and staining of multi-tumor and colon cancer TMAs. (A, B) Multi-tumor TMA (A,B) and colon cancer TMA (C, D) with map information including sample ID were stained with IgG control or anti-RPL22L1. N and EN denote normal tissue samples, while T1 and ET1 denote tumor samples.
(TIF)

S5 Fig Correlation of Rpl22L1 expression with tumor size and lymph node involvement in 23 colon adenocarcinoma patient samples. Data are plotted as percentage of lymph node involvement (A) or tumor size (B) in RPL22L1 low and RPL22L1 high colon adenocarcinoma patient samples. There is a significant correlation between elevated RPL22L1 expression and increased lymph node involvement (A) but not with tumor size (B).
(TIF)

S6 Fig Representative images of Rpl22L1 quantification in TMA-PDX samples. A. Representative images illustrate increased RPL22L1 IHC staining of colon cancer PDX samples (bottom) and in normal colon (top). Staining intensity was quantified using a VECTRA automated quantitative image system (right): green indicates stroma (top right) and brown indicates tumor staining (bottom right). A map of the tumor samples and patient information are found in Table S1 and S2. B. Immunoblot analysis revealed that chronic 5-FU treatment (3mo) of HCT116 or ModeK cells to induce resistance did not result in RPL22L1 induction. The RPL22L1 band is quantified by Image J with background correction and normalized to loading control, Actin.
(TIF)

S7 Fig IHC analysis of DNA mismatch repair proteins, MSH6 and PMS2 to evaluate microsatellite status of PDX colorectal samples. We found 6 of 7 of the RPL22L1 high PDX samples exhibited expression of MSH6 and PMS2. Representative images illustrate RPL22L1 high and RPL22L1 low staining. The top two RPL22L1 high samples are clearly MSS because of the strong nuclear staining of MSH2 and PMS2, while the bottom RPL22L1 low sample appears to be microsatellite unstable (MSI), since it lacks PMS2 staining.
(TIF)

S1 Table Clinical data for patients whose tumor samples were used to generated PDX Samples. n.a.- not analyzed; met–metachonous metastasis; syn–synchronous metastasis.
(TIF)

S2 Table Map, characteristics, and RPL22L1 staining of TMA from colon PDX samples. n.a.–not analyzed; T/C = tumor volume (treated/control); Scores according to T/C value: 50–100% = -; 35–50% = +; 21–35% = ++; 6–20% = +++; 0–5% = ++++. RTV—Relative tumor volument.
(TIF)

S3 Table Correlation between VECTRA quantified RPL22L1 cytoplasmic and nuclear staining and overall survival. Cytoplasmic staining, p = 0.038; Nuclear staining- p = 0.003. RPL22L1 high staining is defined as the top quartile of H scores.
(TIF)

S1 Raw Images (PDF)
