# Supplementary figures and images for: RPL22L1 induction in colorectal cancer is associated with poor prognosis and 5-FU resistance
Source: PLoS One. 2019 Oct 3;14(10):e0222392. doi: 10.1371/journal.pone.0222392 (PMC6776433; doi:10.1371/journal.pone.0222392)

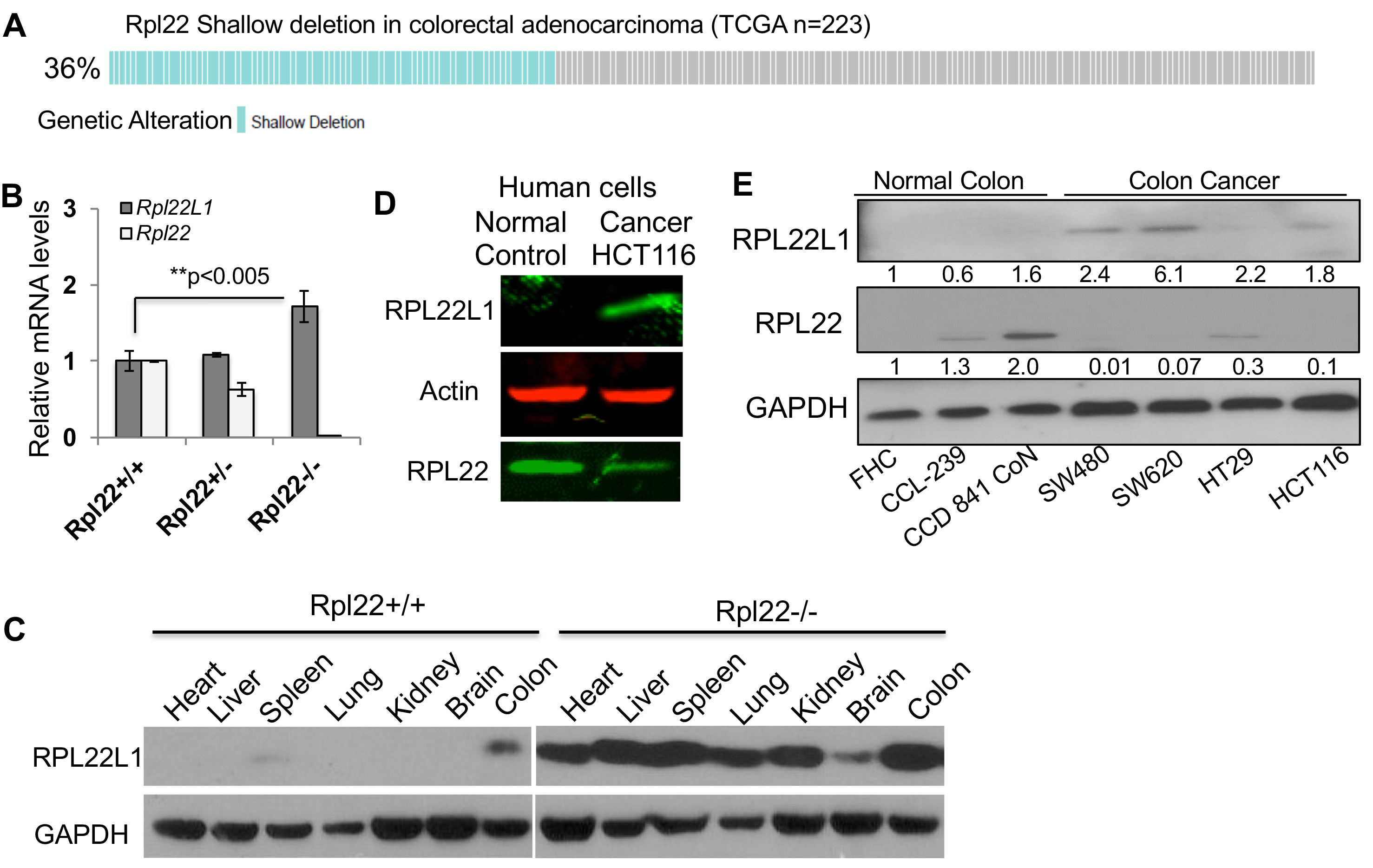

Supplement: S1 Fig — A. RPL22 shallow deletion occurs in up to 36% of CRC samples. Data were collected from TCGA colorectal adenocarcinoma studies n = 223. B. Representative qRT-PCR analysis of Rpl22 and Rpl22l1 mRNA performed in triplicate is plotted graphically as the mean ± SD. Rpl22 and Rpl22l1 mRNA expression was normalized to Gapdh mRNA levels in MEF cells isolated from Rpl22+/+, Rpl22+/- and Rpl22-/- mice. C. Immunoblotting of extracts of tissues collected from Rpl22+/+ and Rpl22-/- mice reveals that RPL22L1 expression is markedly induced by loss of Rpl22. Elevated expression of RPL22L1 was observed in normal colon. D. Immunoblotting of the human CRC cell line, HCT116, reveals that is expresses higher levels of RPL22L1 than the normal colon epithelial cell line CCD841 (Control). E. Immunoblotting of normal colon epithelial cell lines and human CRC cell lines reveals that colon cancer cells express higher levels of RPL22L1 and lower levels of RPL22 relative to normal colon epithelial cell lines. GAPDH served as loading control. Quantification of band intensity by ImageJ is listed on the bottom. (TIF) [file pone.0222392.s001.tif]

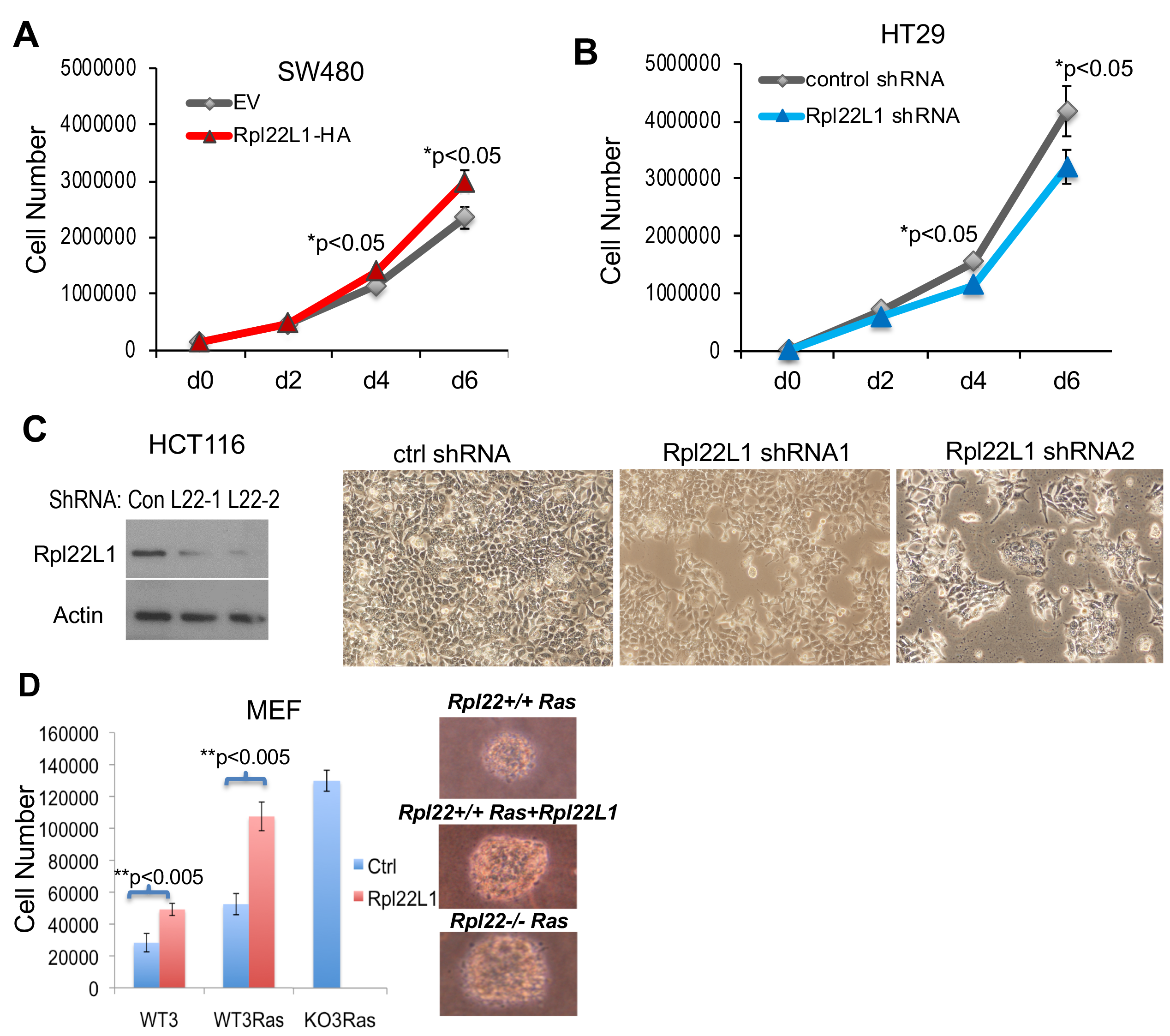

Supplement: S2 Fig — A-B. Overexpression of RPL22L1 (RPL22L1-HA) in SW480 colon cancer cells promotes cell proliferation (A) and knockdown of Rpl22L1 in HT29 colon cancer cells inhibits cell proliferation (B). Data is depicted as average of cell number ± SD from triplicate measurements every two days. Student’s t-test was used for biostatistical analysis between groups (*, p<0.05). C. Knockdown of RPL22L1 with two different shRNA (left) inhibits cell proliferation (right) of HCT116 cells. D. Overexpression of Rpl22L1 in immortalized or Ras-transformed MEF cells promotes cell proliferation as determined by cell numbers (left) and colony formation (right). Student’s t-test was used for statistical analysis between groups. **, p<0.005. (TIF) [file pone.0222392.s002.tif]

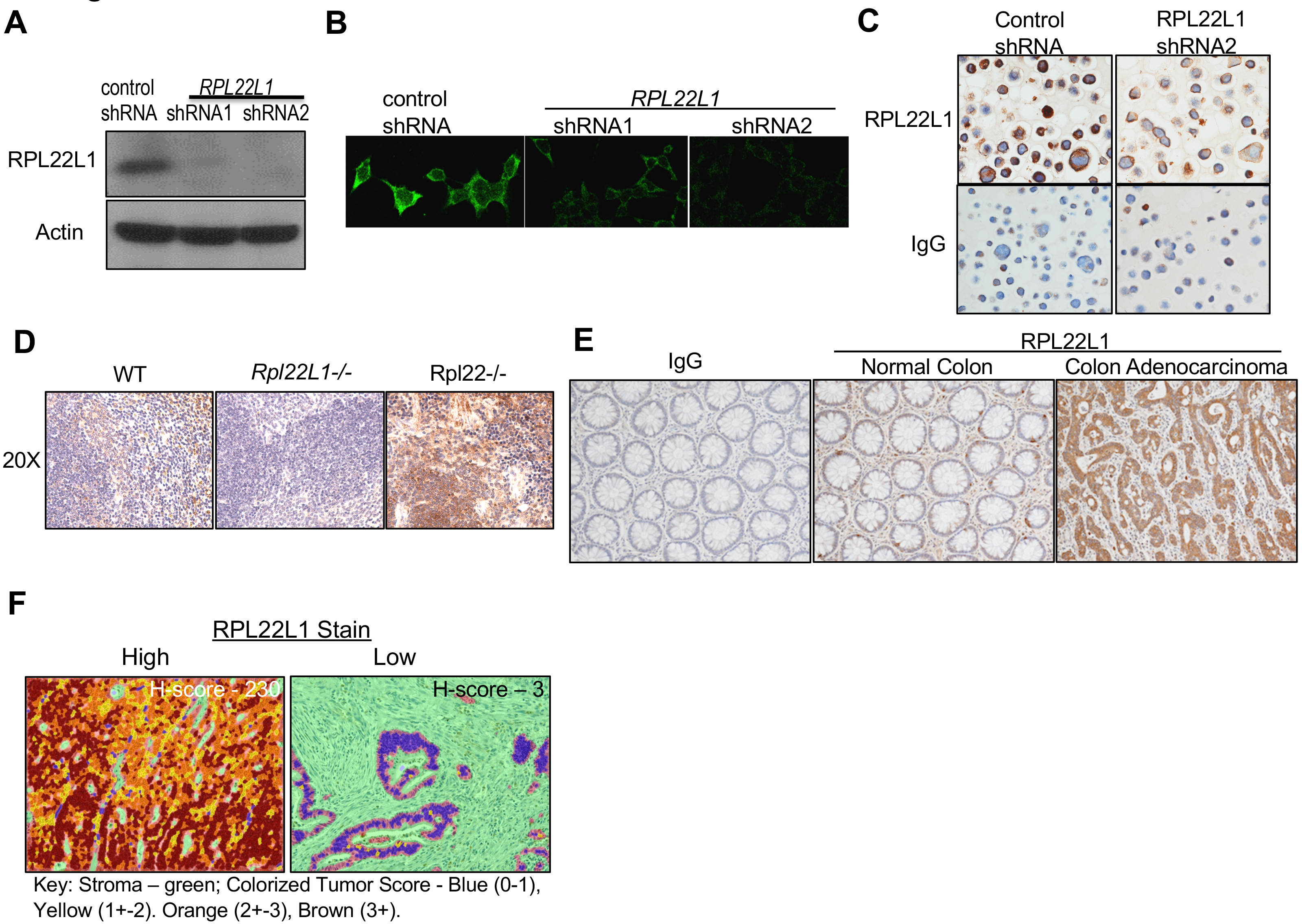

Supplement: S3 Fig — A. Immunoblot analysis reveals that knockdown of RPL22L1 by two different shRNA hairpins in HEK293 cells eliminates immunoreactivity. GAPDH served as loading control. B. Immunofluorescent staining with anti-RPL22L1 followed by FITC anti-rabbit secondary antibody, reveals that RPL22L1 knockdown abrogates staining. C. Knockdown of RPL22L1 in HEK293 cells attenuates staining with anti-RPL22L1 in IHC. D. IHC staining with anti-RPL22L1 of spleens from WT, Rpl22l1-/-, and Rpl22-/- mice. Images at 20x magnification are shown, which validate the specificity of the antibody. E. IHC with anti-RPL22L1 in human normal colon or colon adenocarcinoma samples. IgG in normal colon tissue is used as the negative control. F. Representative image of strong (left) and weak (right) staining of human colon cancer by anti-RPL22L1 in IHC. The staining was quantified by VECTRA automated quantitative image system and presented with H-Score. (TIF) [file pone.0222392.s003.tif]

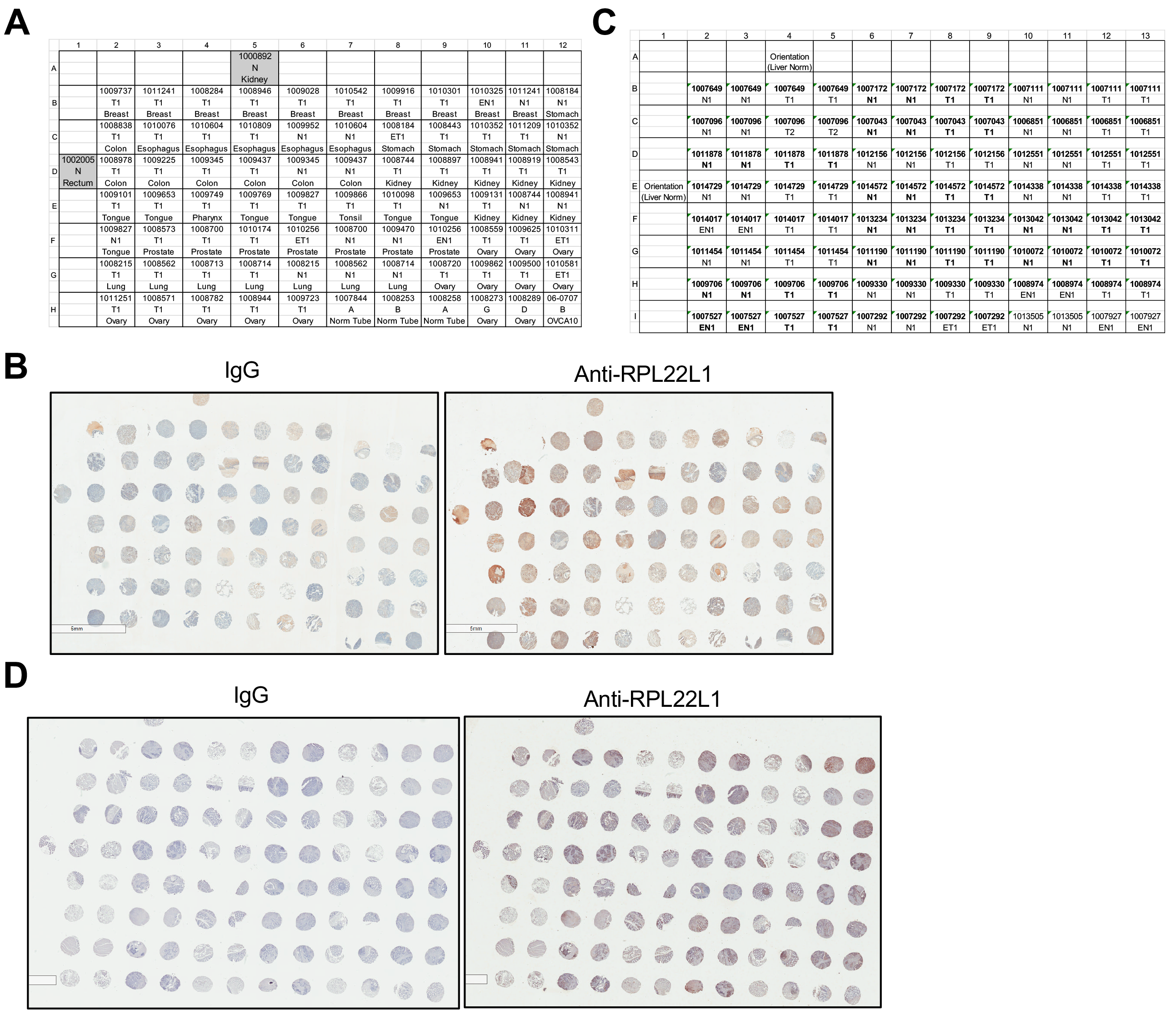

Supplement: S4 Fig — (A, B) Multi-tumor TMA (A,B) and colon cancer TMA (C, D) with map information including sample ID were stained with IgG control or anti-RPL22L1. N and EN denote normal tissue samples, while T1 and ET1 denote tumor samples. (TIF) [file pone.0222392.s004.tif]

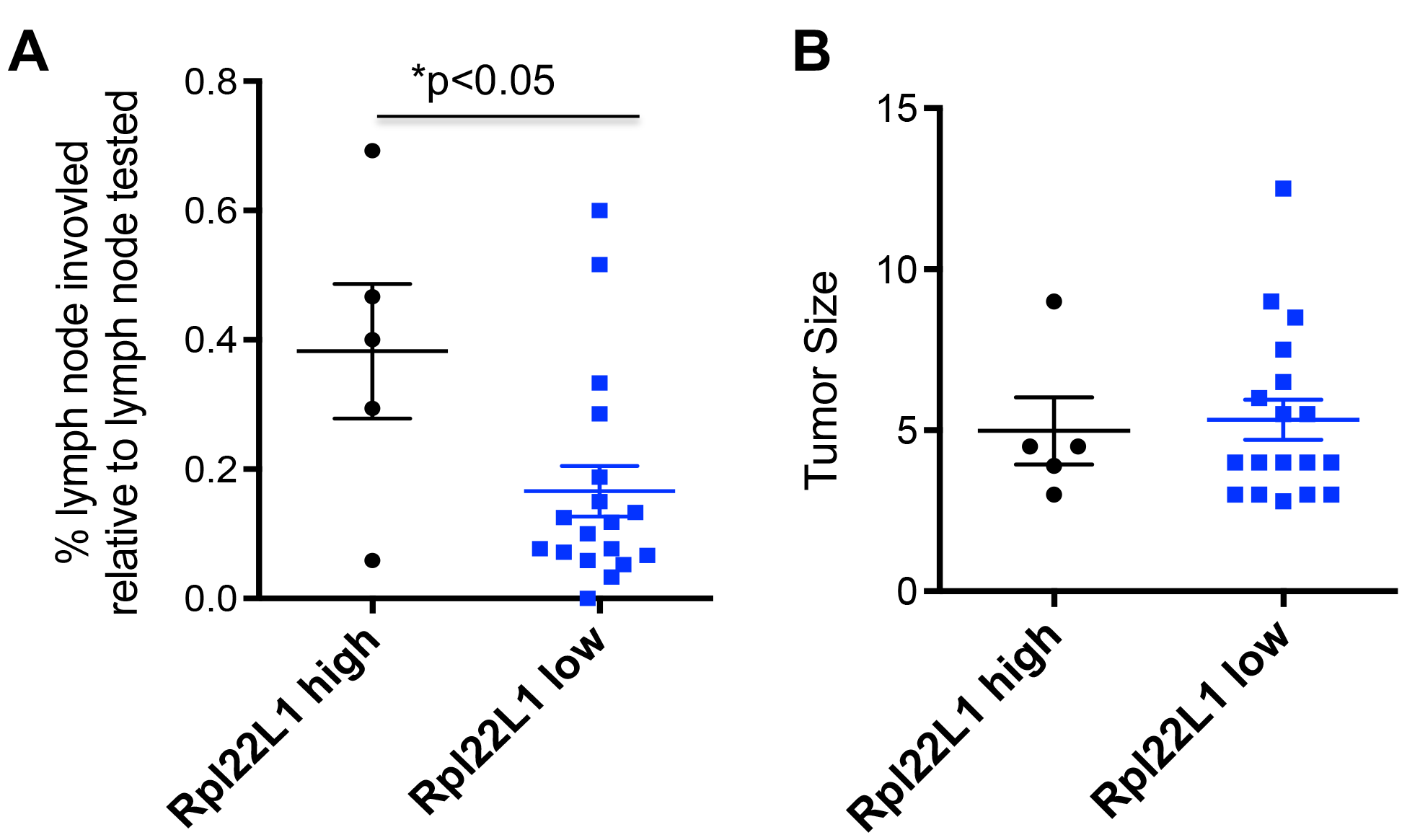

Supplement: S5 Fig — Data are plotted as percentage of lymph node involvement (A) or tumor size (B) in RPL22L1 low and RPL22L1 high colon adenocarcinoma patient samples. There is a significant correlation between elevated RPL22L1 expression and increased lymph node involvement (A) but not with tumor size (B). (TIF) [file pone.0222392.s005.tif]

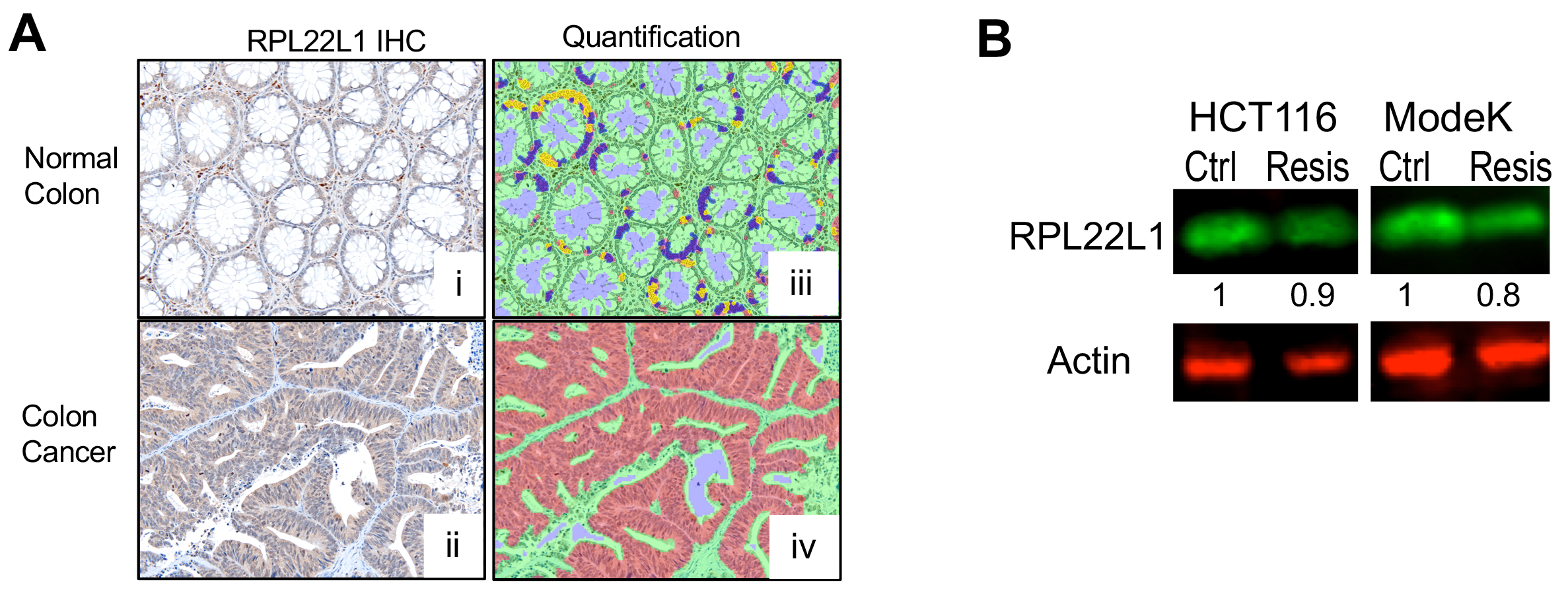

Supplement: S6 Fig — A. Representative images illustrate increased RPL22L1 IHC staining of colon cancer PDX samples (bottom) and in normal colon (top). Staining intensity was quantified using a VECTRA automated quantitative image system (right): green indicates stroma (top right) and brown indicates tumor staining (bottom right). A map of the tumor samples and patient information are found in Table S1 and S2. B. Immunoblot analysis revealed that chronic 5-FU treatment (3mo) of HCT116 or ModeK cells to induce resistance did not result in RPL22L1 induction. The RPL22L1 band is quantified by Image J with background correction and normalized to loading control, Actin. (TIF) [file pone.0222392.s006.tif]

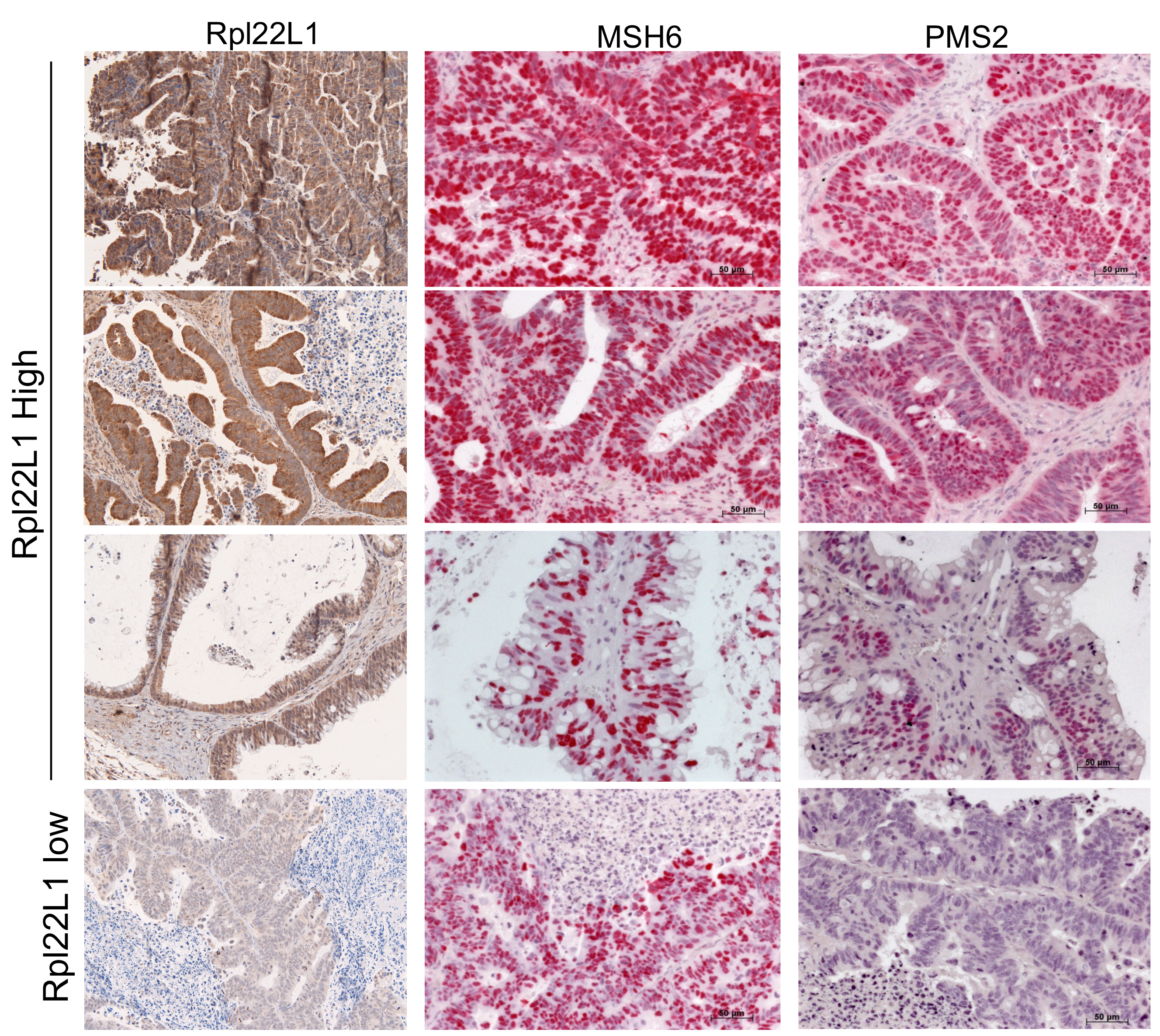

Supplement: S7 Fig — We found 6 of 7 of the RPL22L1 high PDX samples exhibited expression of MSH6 and PMS2. Representative images illustrate RPL22L1 high and RPL22L1 low staining. The top two RPL22L1 high samples are clearly MSS because of the strong nuclear staining of MSH2 and PMS2, while the bottom RPL22L1 low sample appears to be microsatellite unstable (MSI), since it lacks PMS2 staining. (TIF) [file pone.0222392.s007.tif]

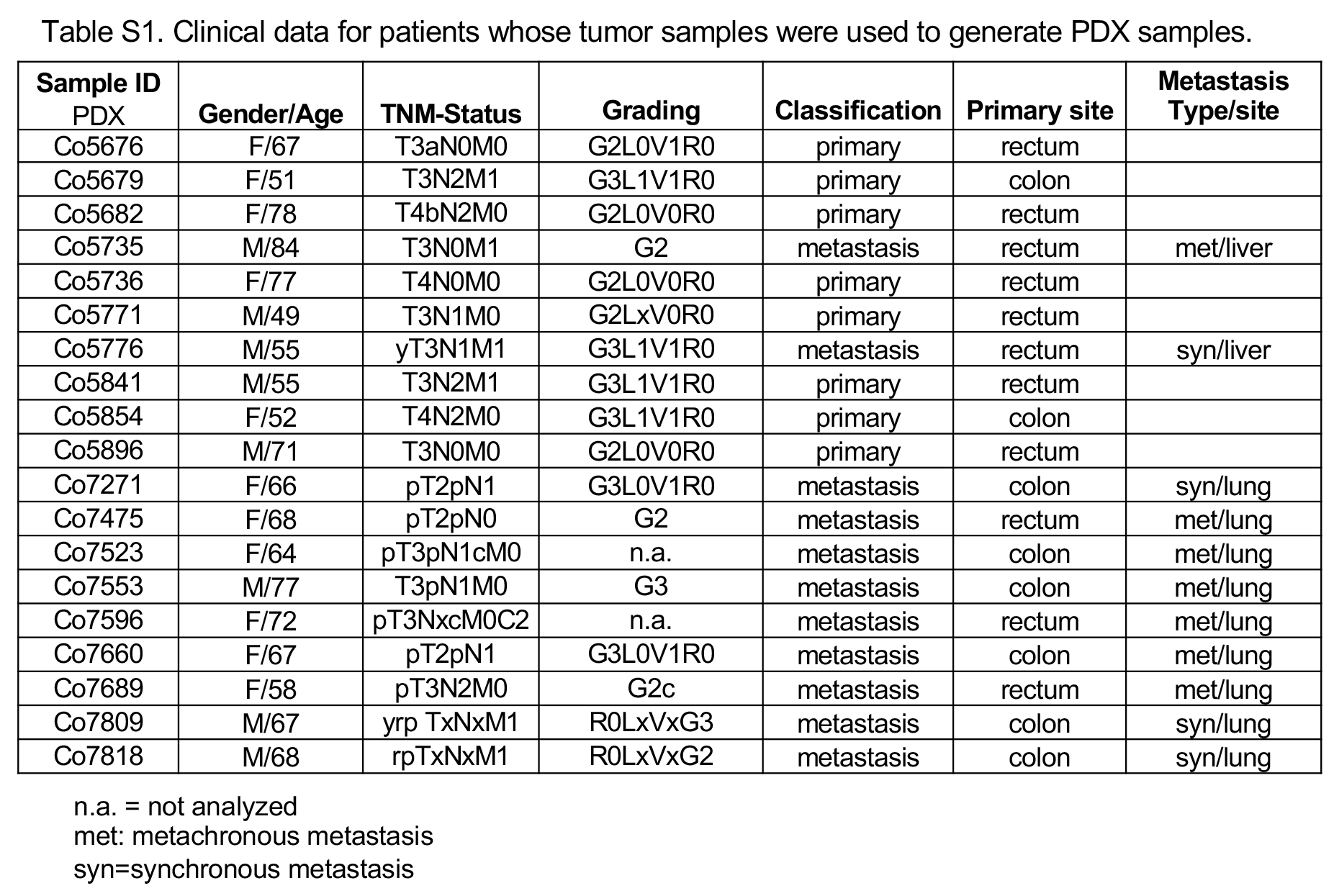

Supplement: S1 Table — n.a.- not analyzed; met–metachonous metastasis; syn–synchronous metastasis. (TIF) [file pone.0222392.s008.tif]

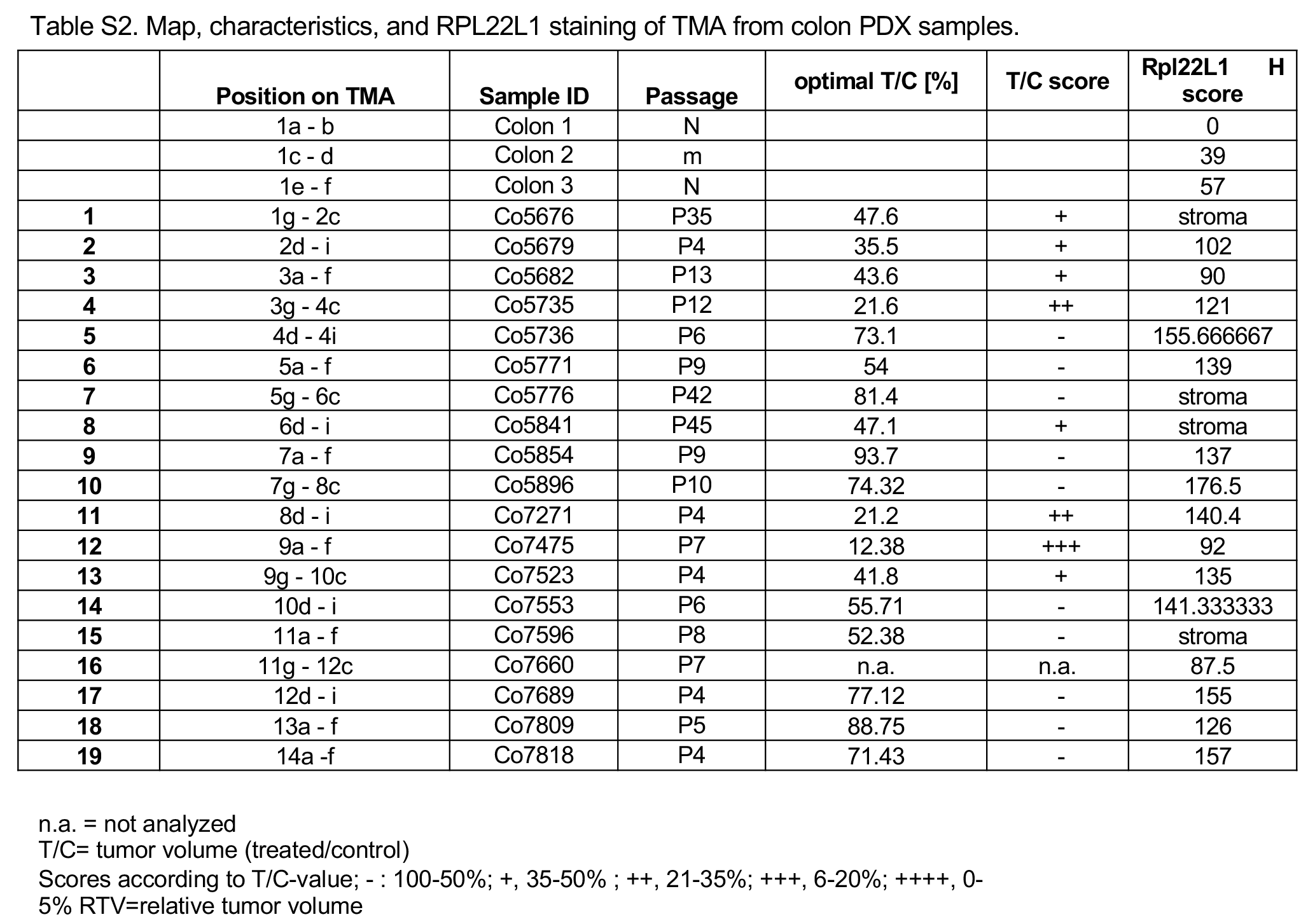

Supplement: S2 Table — n.a.–not analyzed; T/C = tumor volume (treated/control); Scores according to T/C value: 50–100% = -; 35–50% = +; 21–35% = ++; 6–20% = +++; 0–5% = ++++. RTV—Relative tumor volument. (TIF) [file pone.0222392.s009.tif]

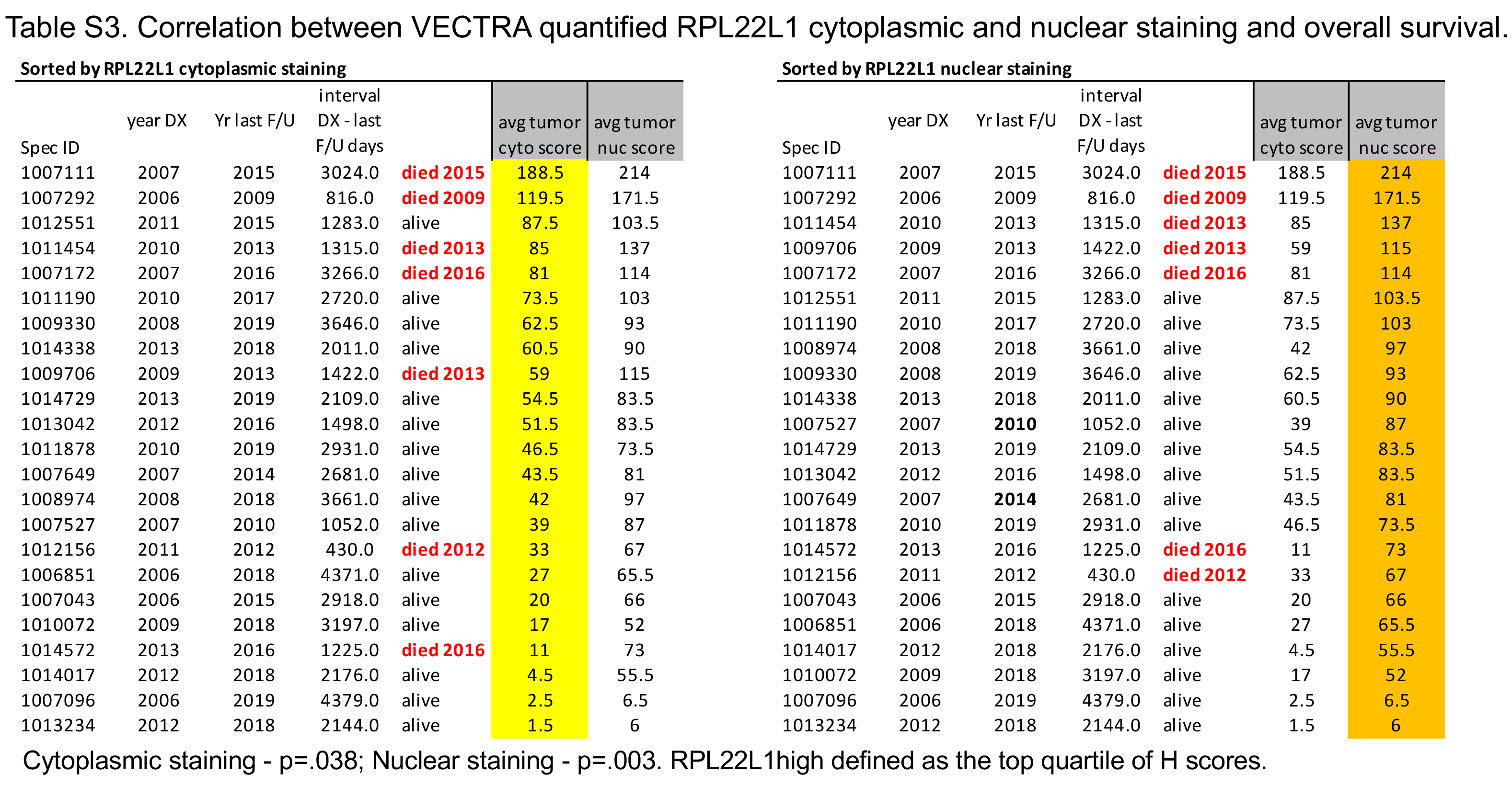

Supplement: S3 Table — Cytoplasmic staining, p = 0.038; Nuclear staining- p = 0.003. RPL22L1 high staining is defined as the top quartile of H scores. (TIF) [file pone.0222392.s010.tif]
