# Supplementary material for: RPL22L1 induction in colorectal cancer is associated with poor prognosis and 5-FU resistance
Source: PLoS One. 2019 Oct 3;14(10):e0222392. doi: 10.1371/journal.pone.0222392 (PMC6776433; doi:10.1371/journal.pone.0222392)

Figure 1A Rpl22L1: Raw western blot of **anti-Rpl22L1** (red arrow) in MEF cell lysates from Rpl22+/+, Rpl22+/- and Rpl22-/- mice for Fig 1A. Image was developed with luminol-based Enhanced Chemiluminescent (ECL) western blotting substrate. Film was cut before the imaging development together with other membranes (X) at the same time. Square indicates the band used in Fig1A.

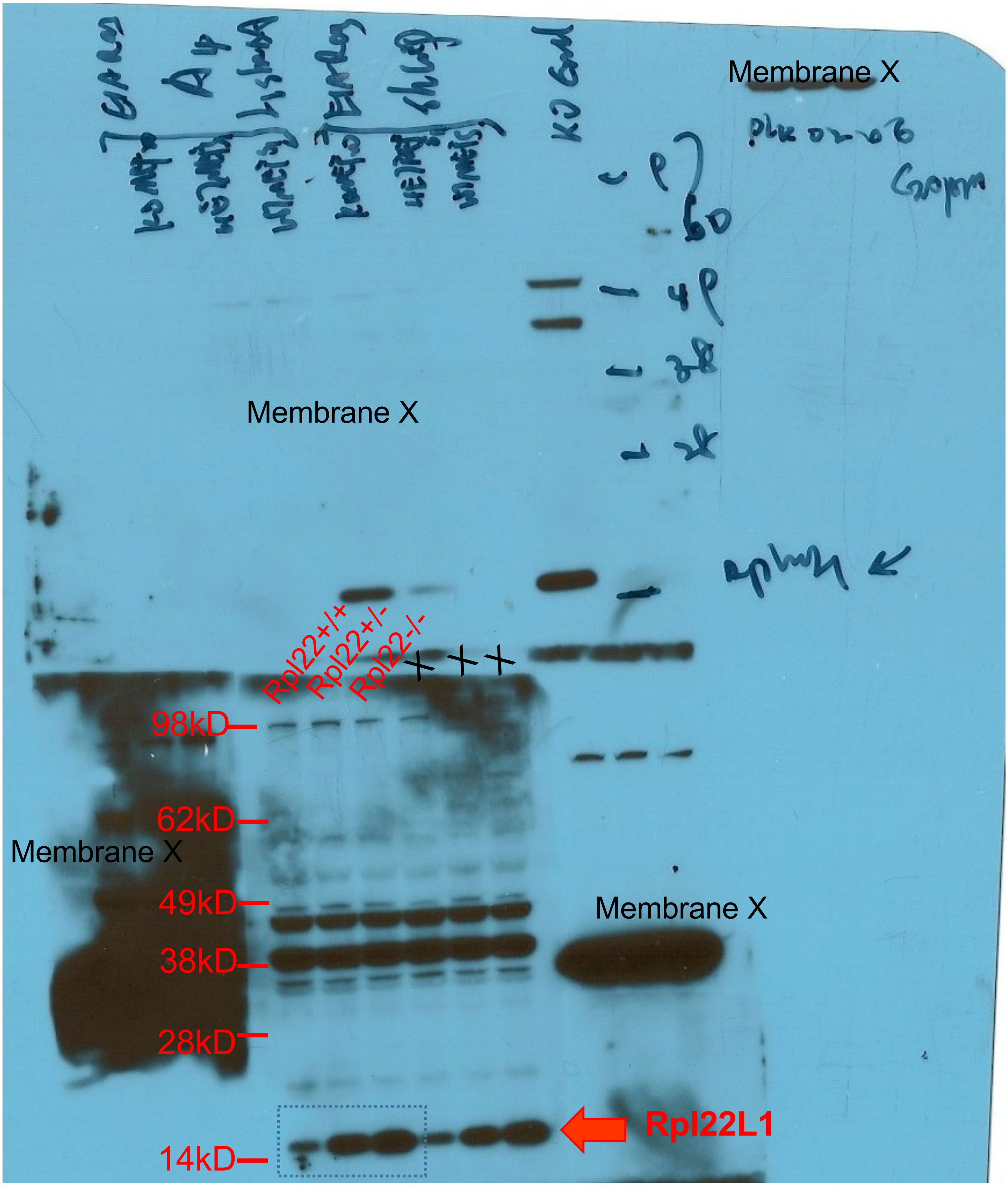

Figure 1A GAPDH: Raw western blot of **anti-GAPDH** (red arrow) in MEF cell lysates from Rpl22+/+, Rpl22+/- and Rpl22-/- mice for Fig 1A. Image was developed with luminol-based Enhanced Chemiluminescent (ECL) western blotting substrate. Film was cut before the imaging development on several different membranes probed with anti-GAPDH at the same time. Film was moved to get bands at different exposure time. Square indicates the band used in Fig1A.

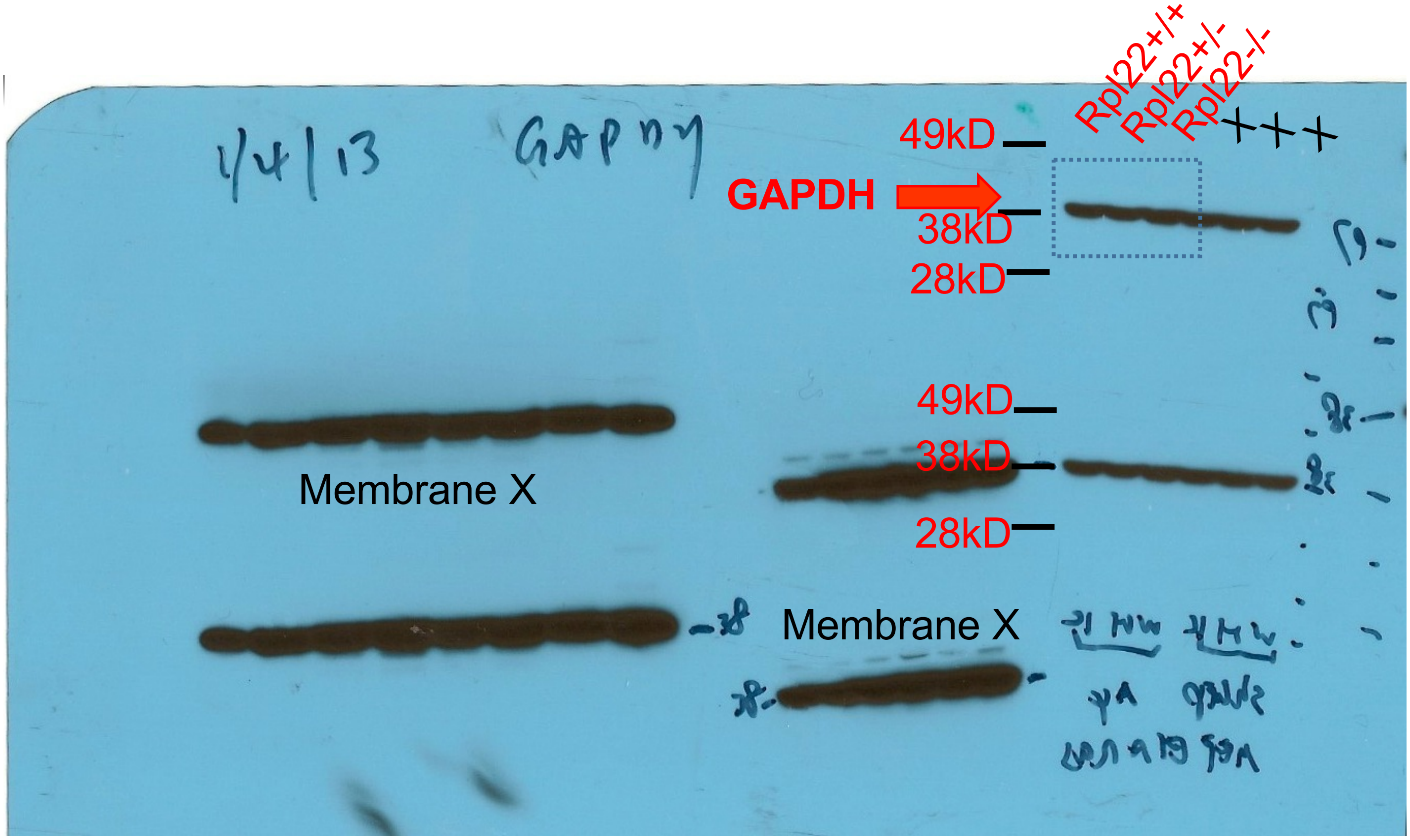

Figure 1B Rpl22, Rpl22L1 and Actin: Raw western blot of **anti-Rpl22** (green band with red arrow on left side), **anti-Rpl22L1** (green band with red arrow on the right side**)** and **anti-Actin** (red band with red arrow) in cell lysates from ModeK cells with control shRNA, Rpl22 shRNA1 and Rpl22 shRNA2 for Fig 1B. Image was developed with LI-COR Odyssey infrared (IR) fluorescence image system. Membranes were cut before antibodies application. The image was captured together with other membranes probed with anti-Actin, anti-Rpl22 or anti-Rpl22L1. Square indicates bands used in Fig1B.

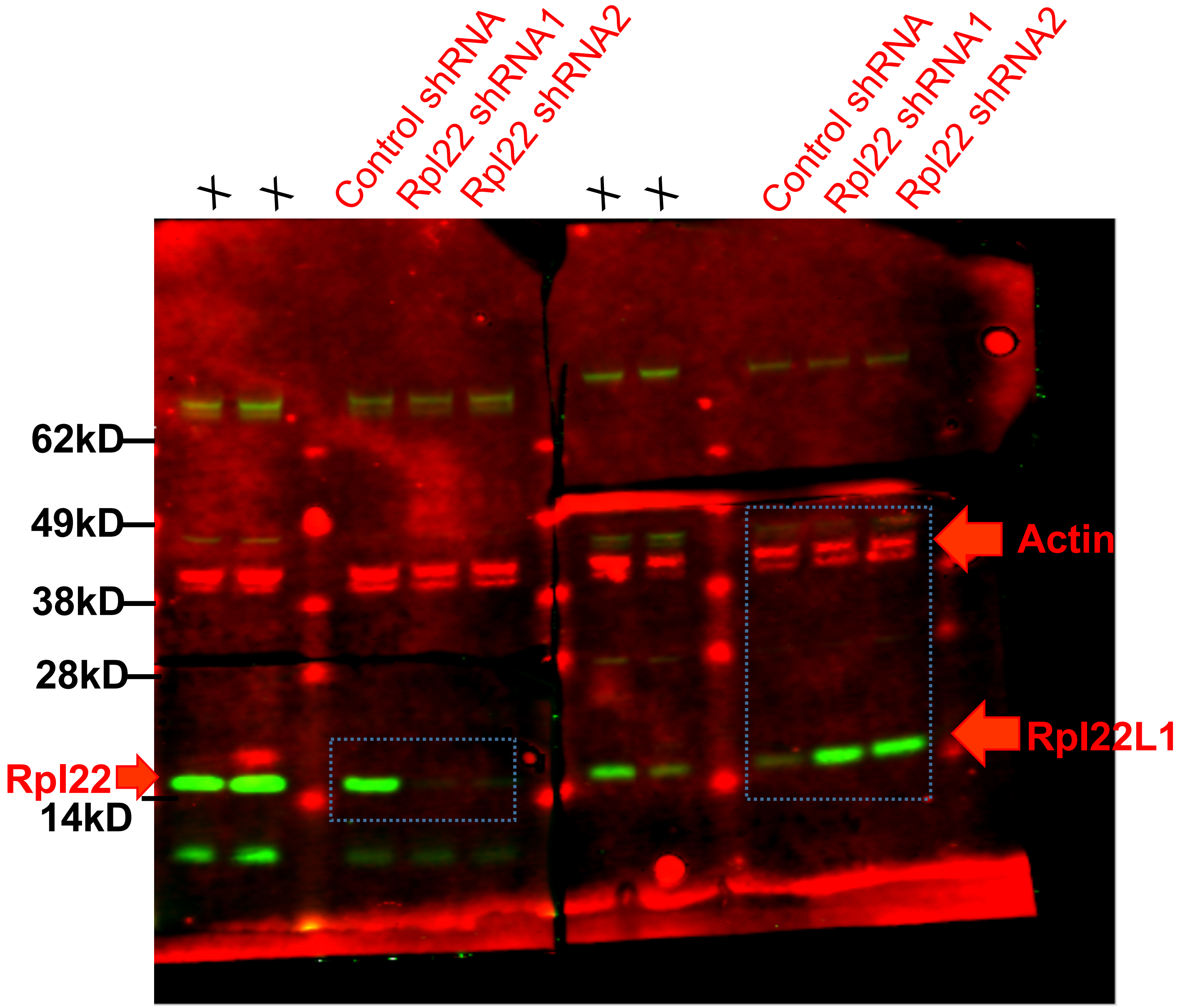

Figure 1C Rpl22L1 and Actin: Raw western blot of **anti-Rpl22L1** (green band with red arrow) in cell lysates from HCT116 cells with control shRNA, Rpl22 shRNA1 and Rpl22 shRNA2 for Fig 1C. Image was developed with LI-COR Odyssey infrared (IR) fluorescence image system. Membrane was cut before antibodies application. The image was captured together with other membranes probed with anti-Actin or anti-Rpl22L1 at the same time. Square indicates bands used in Fig 1C.

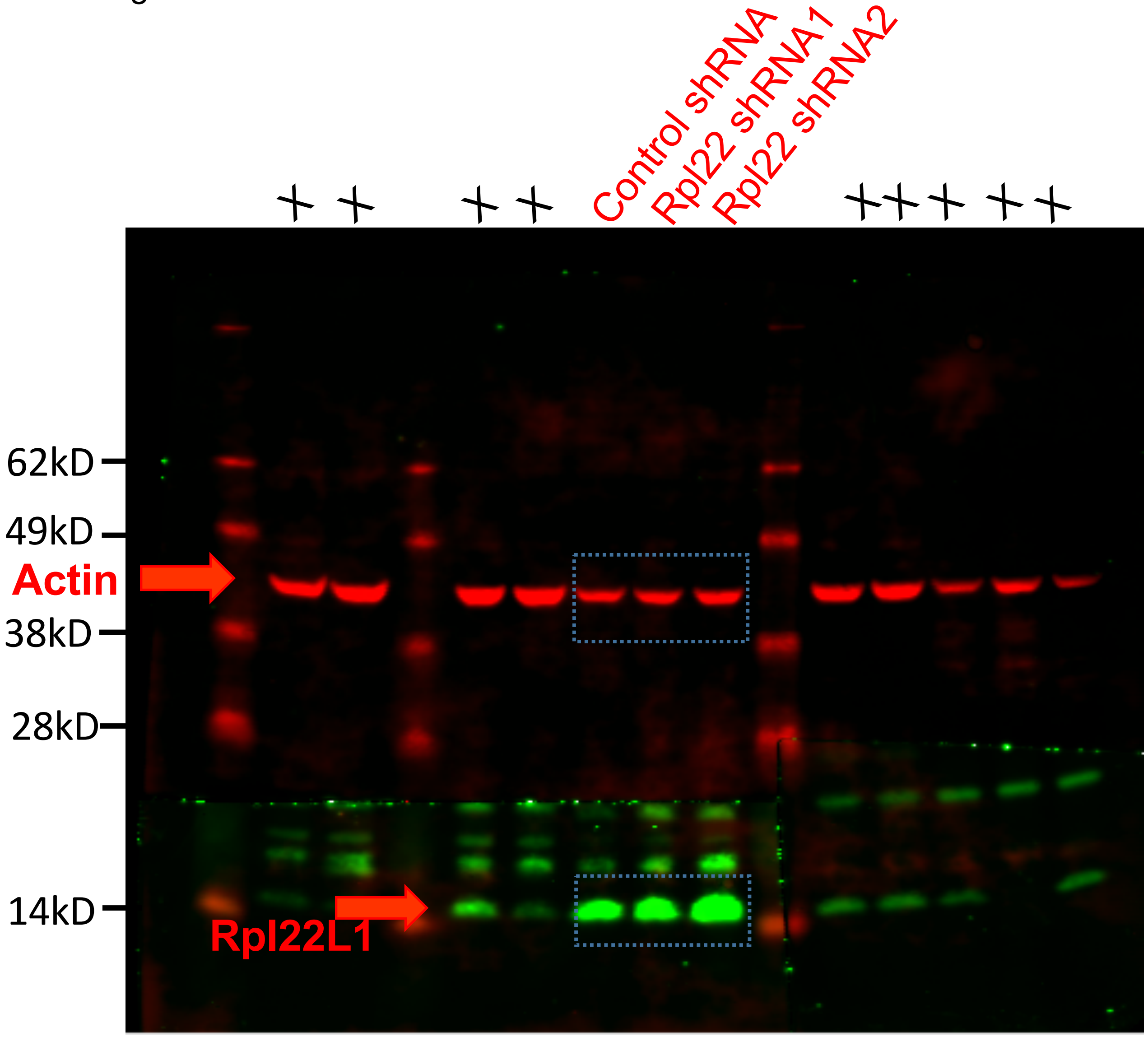

Figure 1D Rpl22L1 and GFP-Rpl22: Raw western blot of **anti-Rpl22L1** (green band with red arrow) and anti-GFP to detect GFP-Rpl22 (green band with red arrow) in cell lysates from HCT116 cells with control vector or GFP-Rpl22 for Fig 1D. Image was developed with LI-COR Odyssey infrared (IR) fluorescence image system. Membrane was cut before antibodies application. The image was captured together with other membrane probed with different antibodies. Square indicates bands used in Fig 1D.

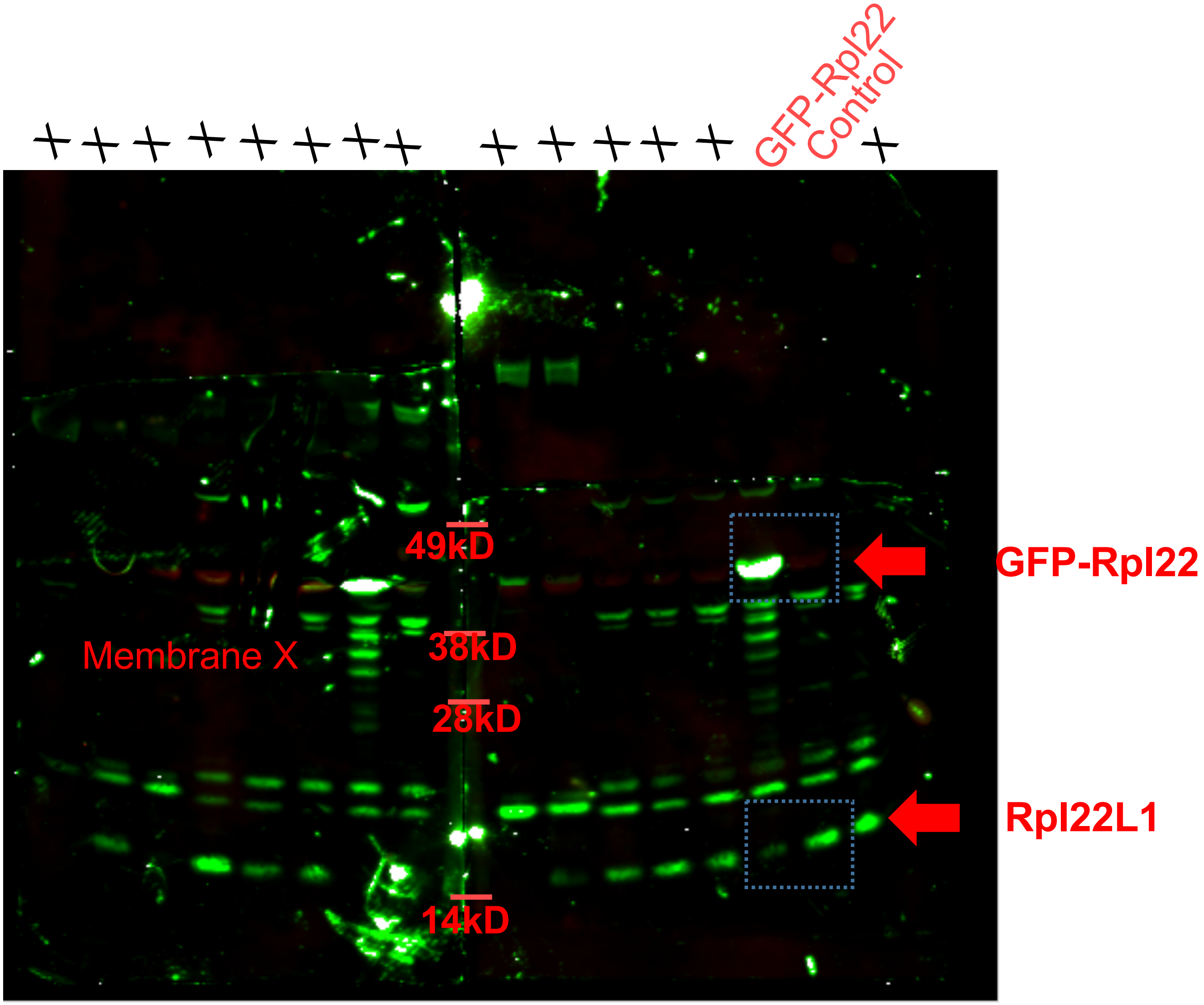

Figure 1D Actin: Raw western blot of **anti-Actin** (red band with red arrow) in cell lysates from HCT116 cells with control vector or GFP-Rpl22 for Fig 1D. Image was developed with LI-COR Odyssey infrared (IR) fluorescence image system. Membrane was cut before antibody application. Square indicates bands used in Fig 1D.

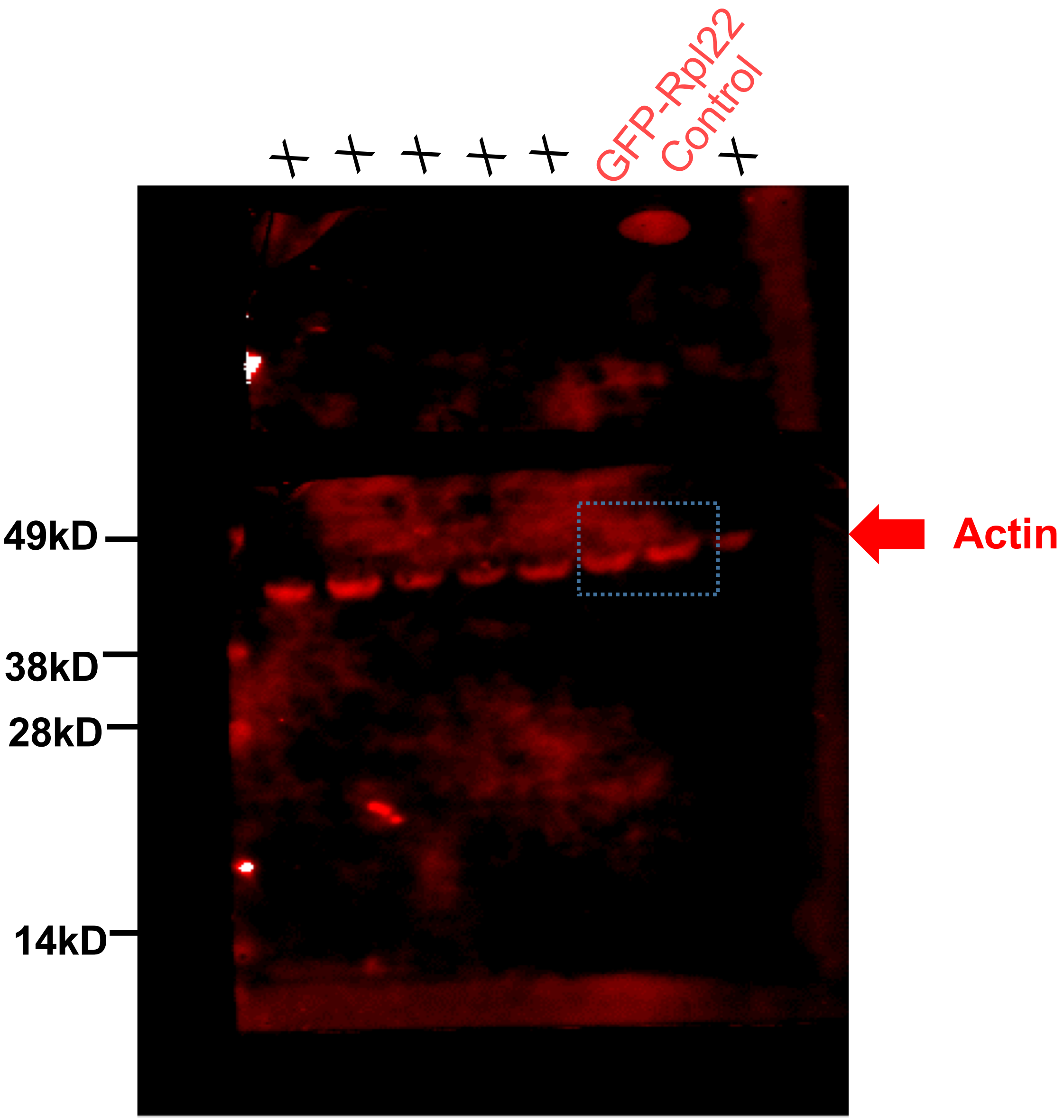

Figure 1E Rpl22L1,Tubulin and GAPDH: Raw western blot of **anti-Rpl22L1** (green band with red arrow in top image) and anti-Tubulin and anti-GAPDH (red band with red arrow in bottom image) in colon tumor tissue lysates from colon adenocarcinoma patients labeled with indicated sample ID number for Fig 1E. Image was developed with LI-COR Odyssey infrared (IR) fluorescence image system. Square indicates bands used in Fig 1E. * indicates non-specific band.

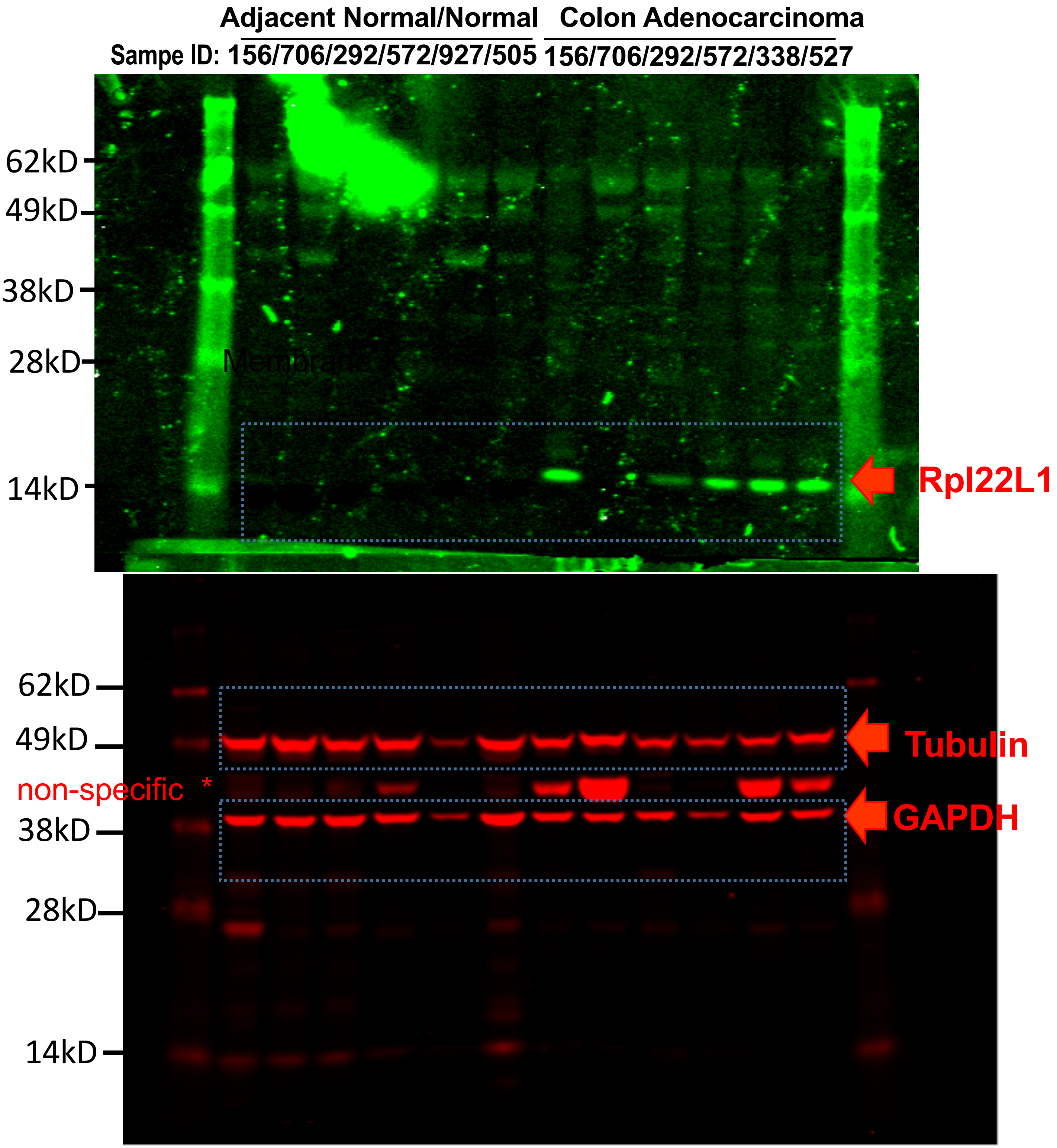

Figure 1E Rpl22: Raw western blot of **anti-Rpl22** (green band with red arrow) in colon tumor tissue lysates from colon adenocarcinoma patients labeled with indicated sample ID number for Fig 1E. Image was developed with LI-COR Odyssey infrared (IR) fluorescence image system. Square indicates band used in Fig 1E. * indicates non-specific band.

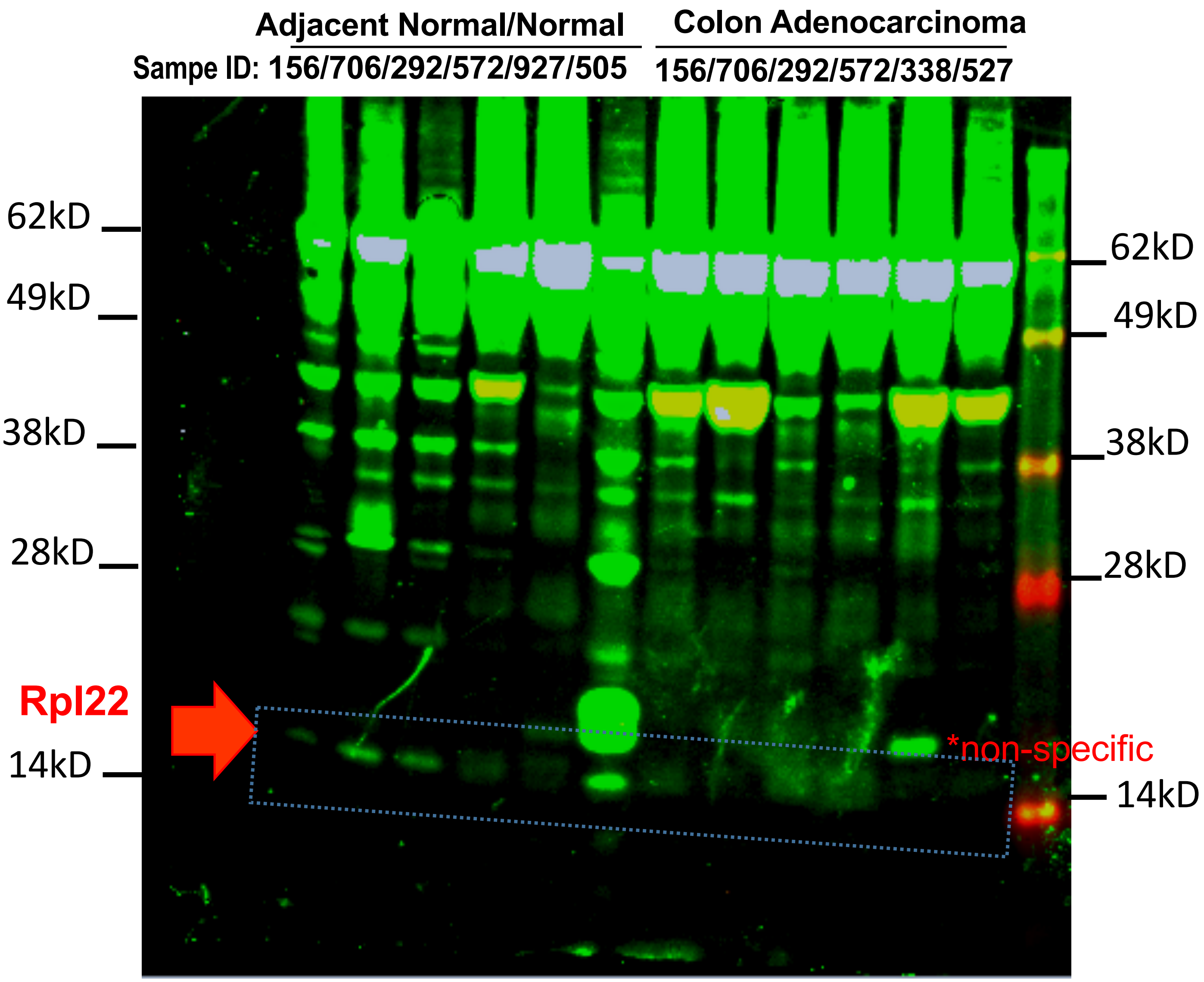

Figure 2A Rpl22L1-HA and Actin: Raw western blot of **anti-Actin** (red band with red arrow, top image) and **anti-HA** to detect Rpl22L1-HA (red band with red arrow, bottom image) in ModeK cells with control vector (EV) or Rpl22L1-HA for Fig 2A. Image was developed with LI-COR Odyssey infrared (IR) fluorescence image system. Membrane was cut before antibodies application. For the bottom image to detect Rpl22L1-HA, only this part of the membrane was scanned. Square indicates bands used in Fig 2A.

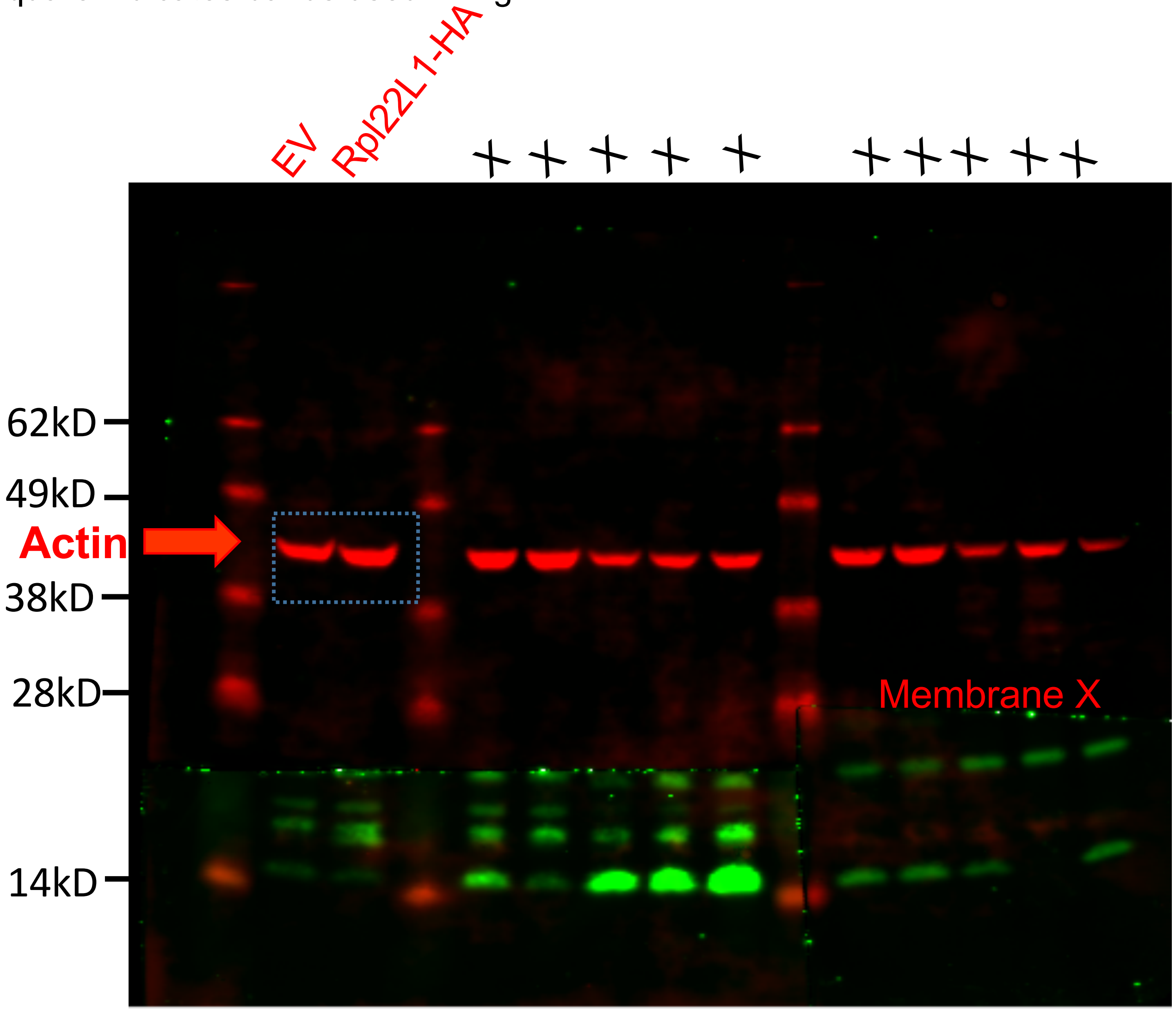

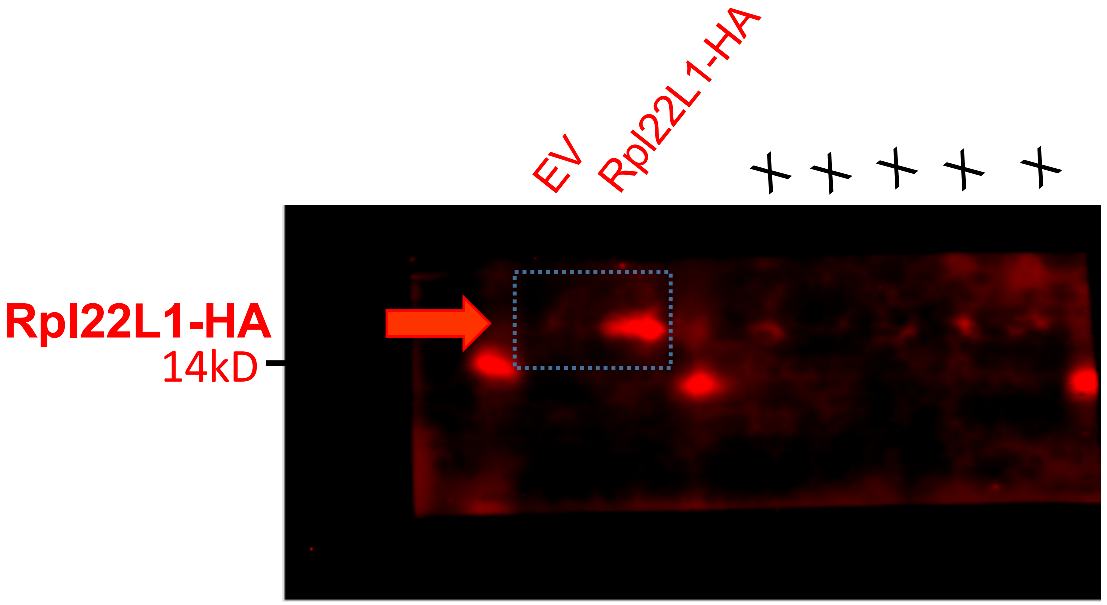

Figure 2B Rpl22L1 and Actin: Raw western blot of **anti-Rpl22L1** (green band with red arrow) and **anti-Actin** (red band with red arrow) in cell lysates from ModeK cells with control shRNA, or Rpl22L1 shRNA for Fig 2B. Image was developed with LI-COR Odyssey infrared (IR) fluorescence image system. Membrane was cut before antibodies application. The image was captured together with other membranes at the same time. Square indicates bands used in Fig 2B.

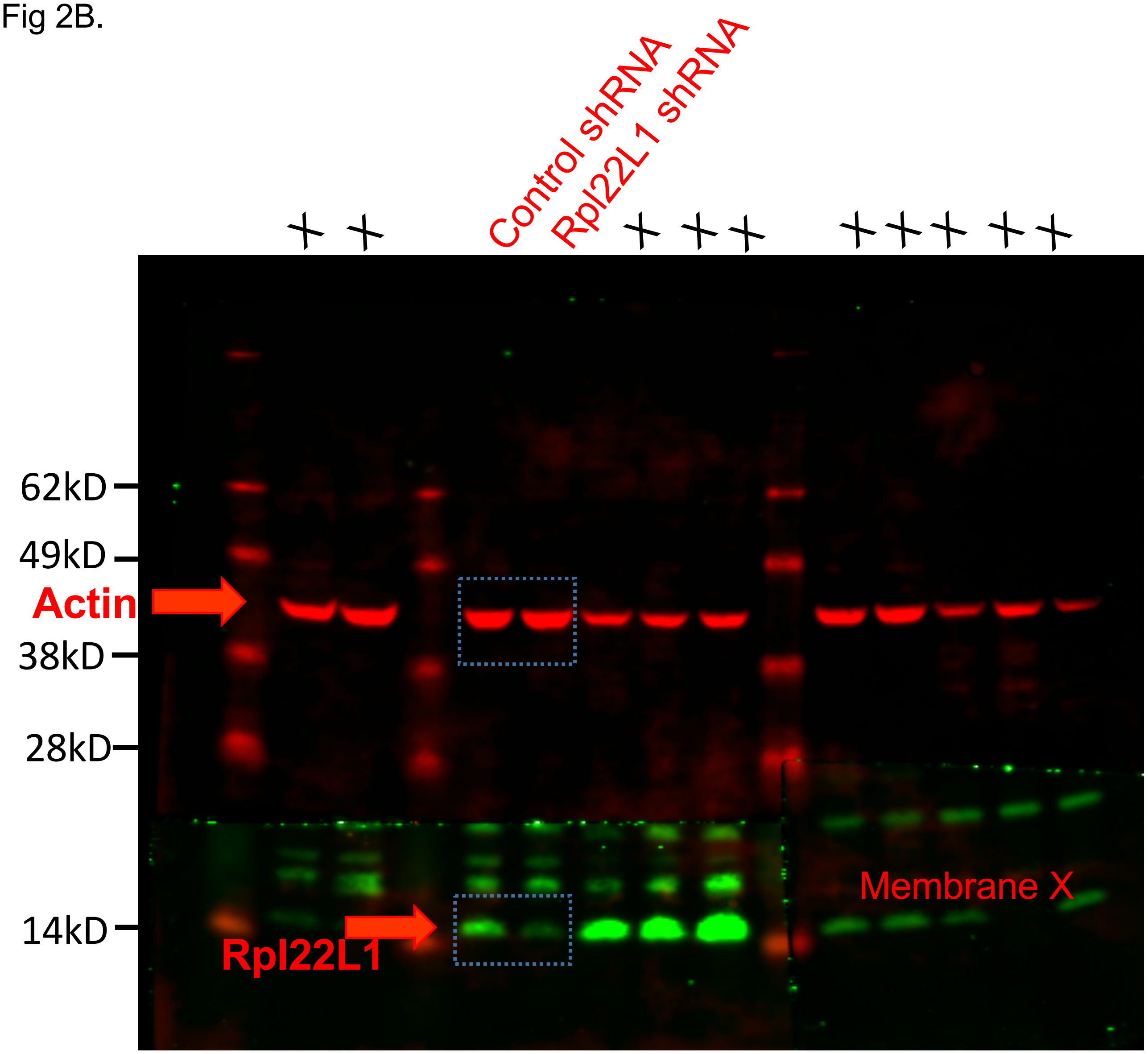

Figure 2E and 2F Actin: Raw western blot of **anti-Actin** (red arrow) in lysates from different colon cancer cell lines, SW620 and HT29 with control shRNA or Rpl22L1 shRNA for Fig 2E and 2F. Image was developed with luminol-based Enhanced Chemiluminescent (ECL) western blotting substrate. Film was cut before the imaging development together with other membranes (X) at the same time. Square indicates the band used in Fig 2E and Fig 2F.

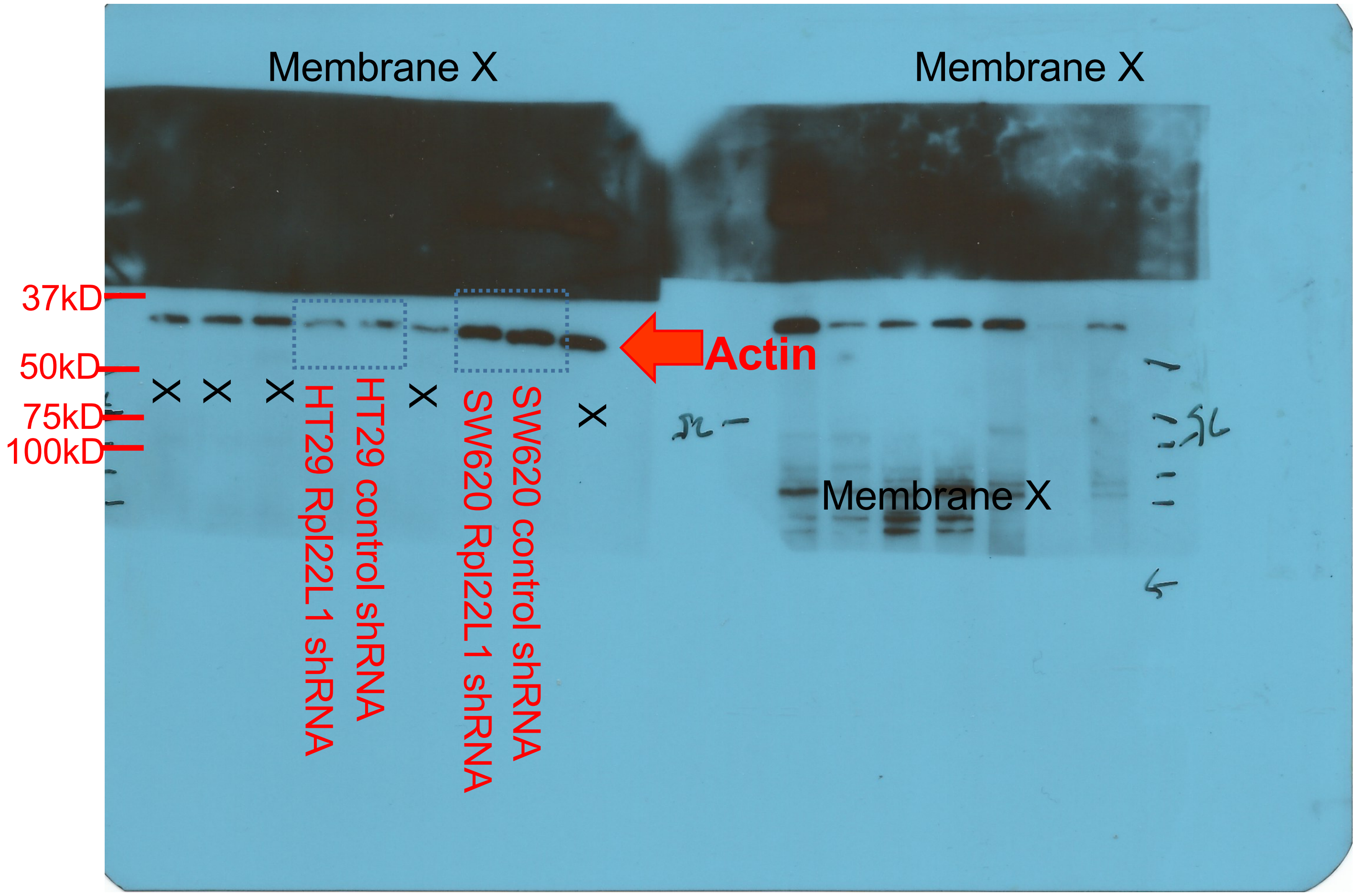

Figure 2E and 2F Rpl22L1: Raw western blot of **anti-Rpl22L1** (red arrow) in lysates from different colon cancer cell lines, SW620 and HT29 with control shRNA or Rpl22L1 shRNA for Fig 2E and 2F. Image was developed with luminol-based Enhanced Chemiluminescent (ECL) western blotting substrate. Film was cut before the imaging development together with other membranes (X) at the same time. Square indicates the band used in Fig 2E and Fig 2F.

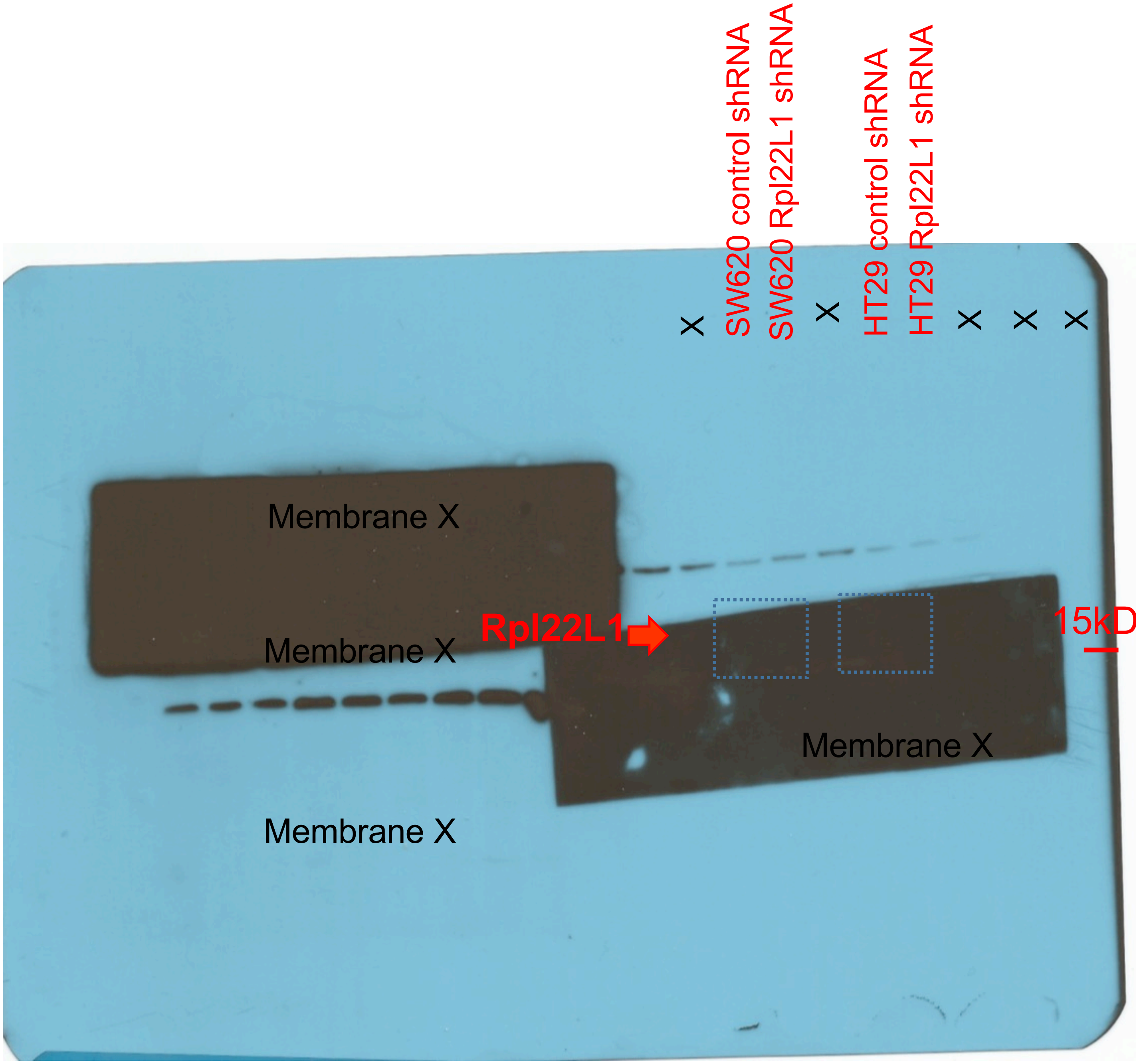

Figure 5A Rpl22L1 and Actin: Raw western blot of **anti-Rpl22L1** (green band with red arrow) and **anti-Actin** (red band with red arrow) in cell lysates from HCT116 cells with control shRNA1, Rpl22L1 shRNA and control shRNA2 for Fig 5A. Image was developed with LI-COR Odyssey infrared (IR) fluorescence image system. Membrane was cut before antibodies application. The image was captured together with other membranes at the same time. Square indicates bands used in Fig 5A.

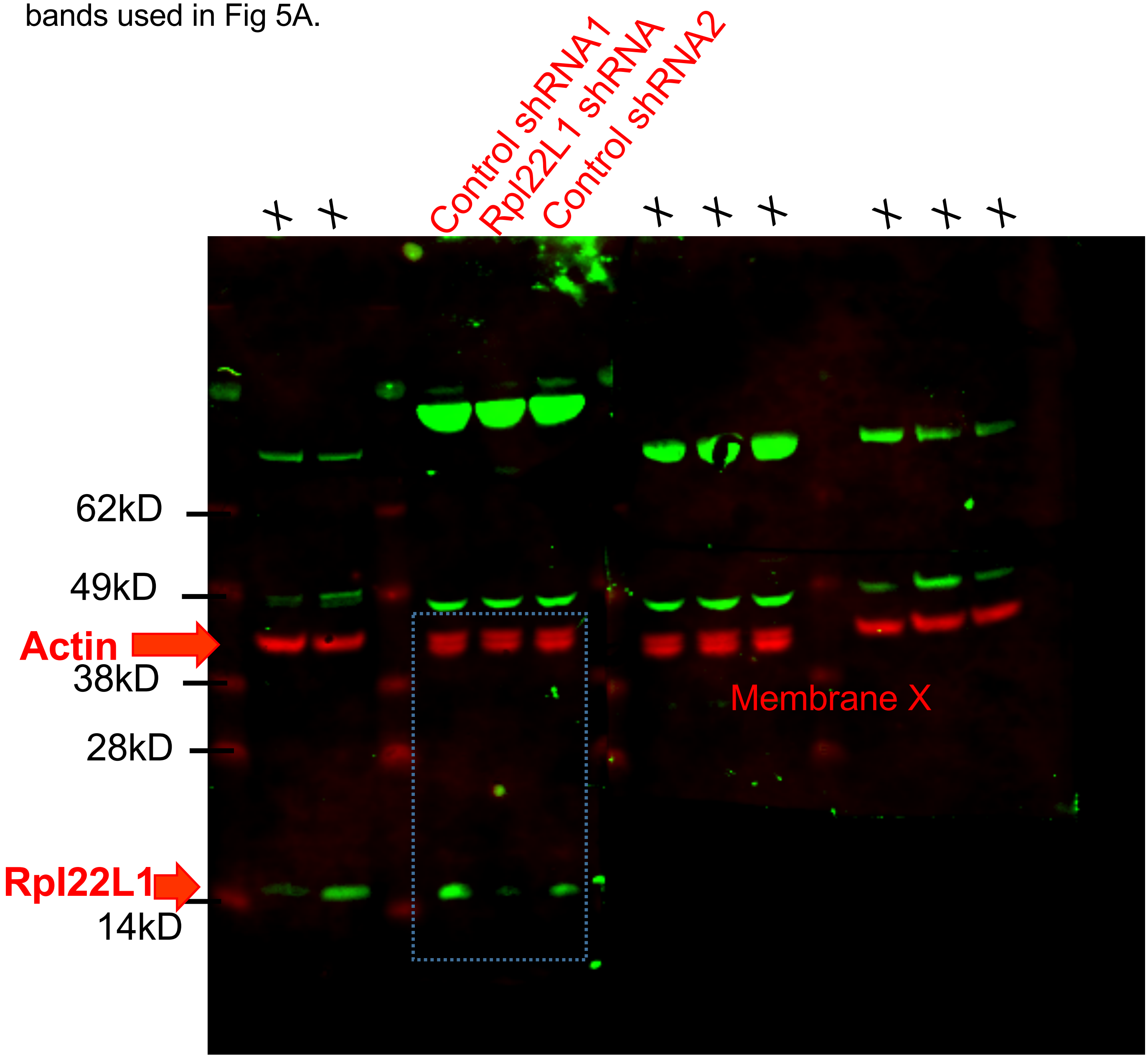

Figure 6A MGMT in ModeK cells: Raw western blot of **anti-MGMT** (green band with red arrow) in cell lysates from ModeK cells with control vector (EV) or Rpl22L1-HA for Fig 6A. Image was developed with LI-COR Odyssey infrared (IR) fluorescence image system. Square indicates the band used in Fig 6A.

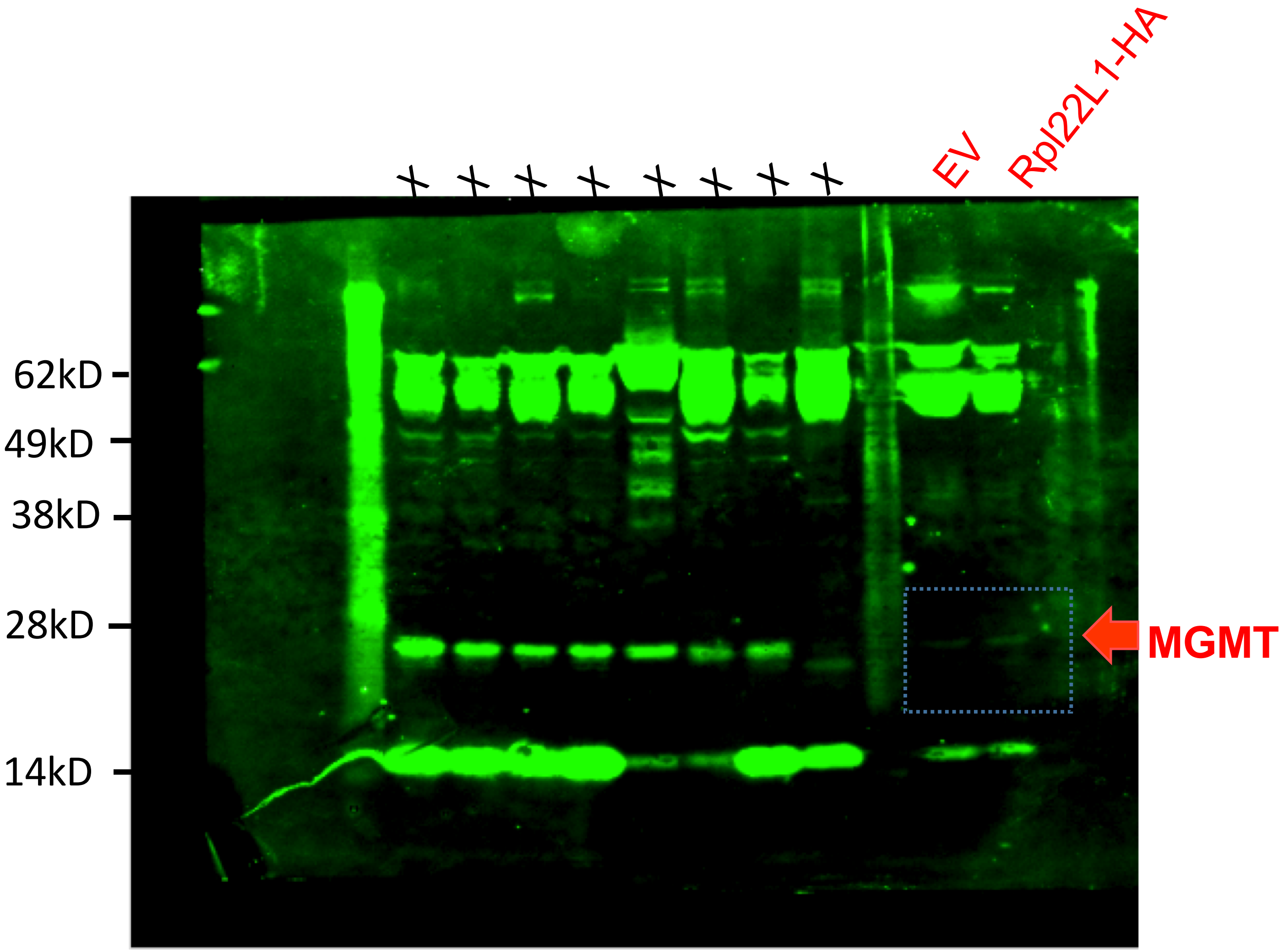

Figure 6A Actin in ModeK cells: Raw western blot of **anti-Actin** (red band with red arrow) in cell lysates from ModeK cells with control vector (EV) or Rpl22L1-HA for Fig 6A. Image was developed with LI-COR Odyssey infrared (IR) fluorescence image system. Square indicates the band used in Fig 6A.

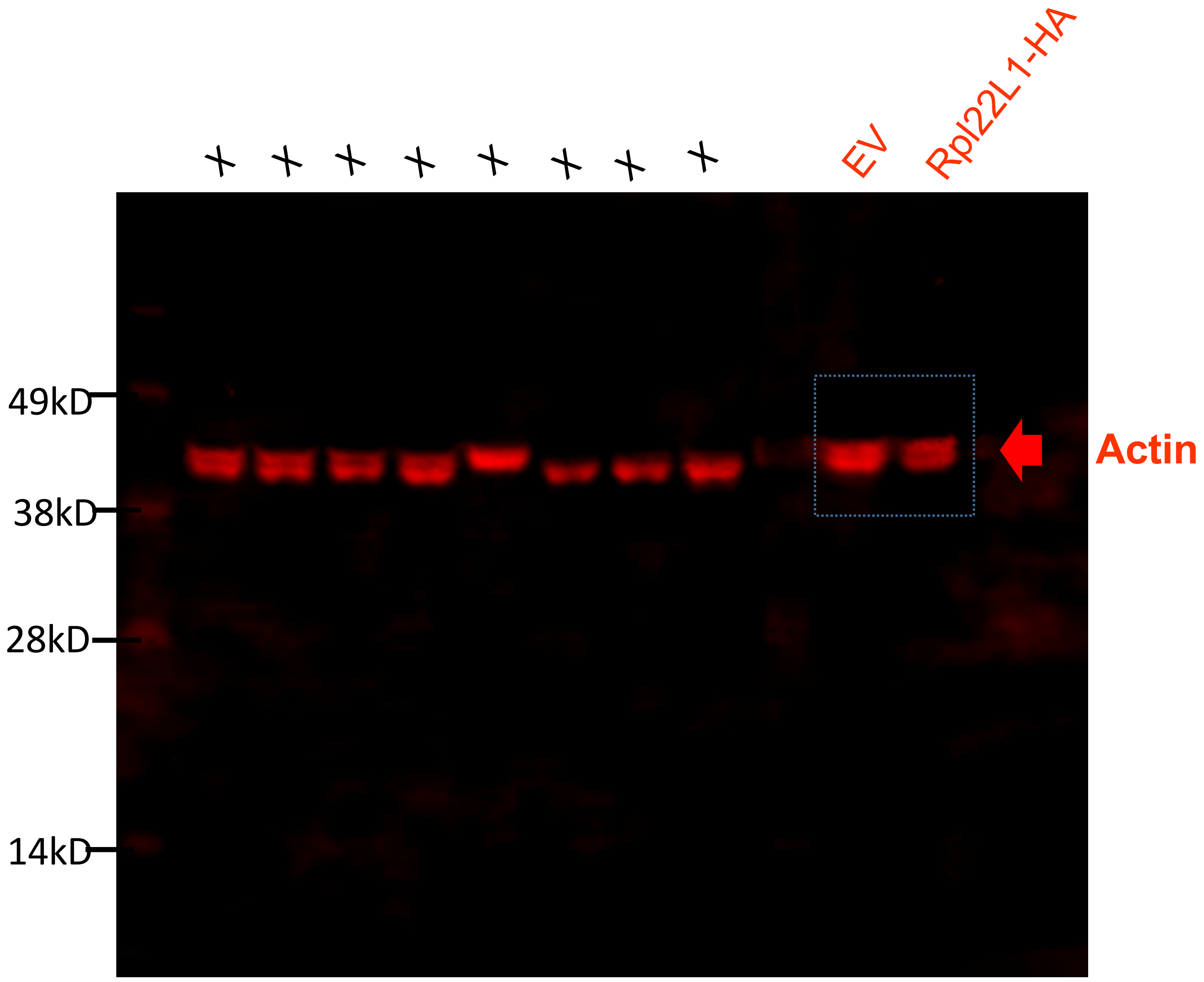

Figure 6A MLH1 in ModeK cells: Raw western blot of **anti-MLH1** (black band with red arrow) in cell lysates from ModeK cells with control vector (EV) or Rpl22L1-HA for Fig 6A. Image was developed with LI-COR Odyssey infrared (IR) fluorescence image system but converted to black-white color. Square indicates the image used in Fig 6A.

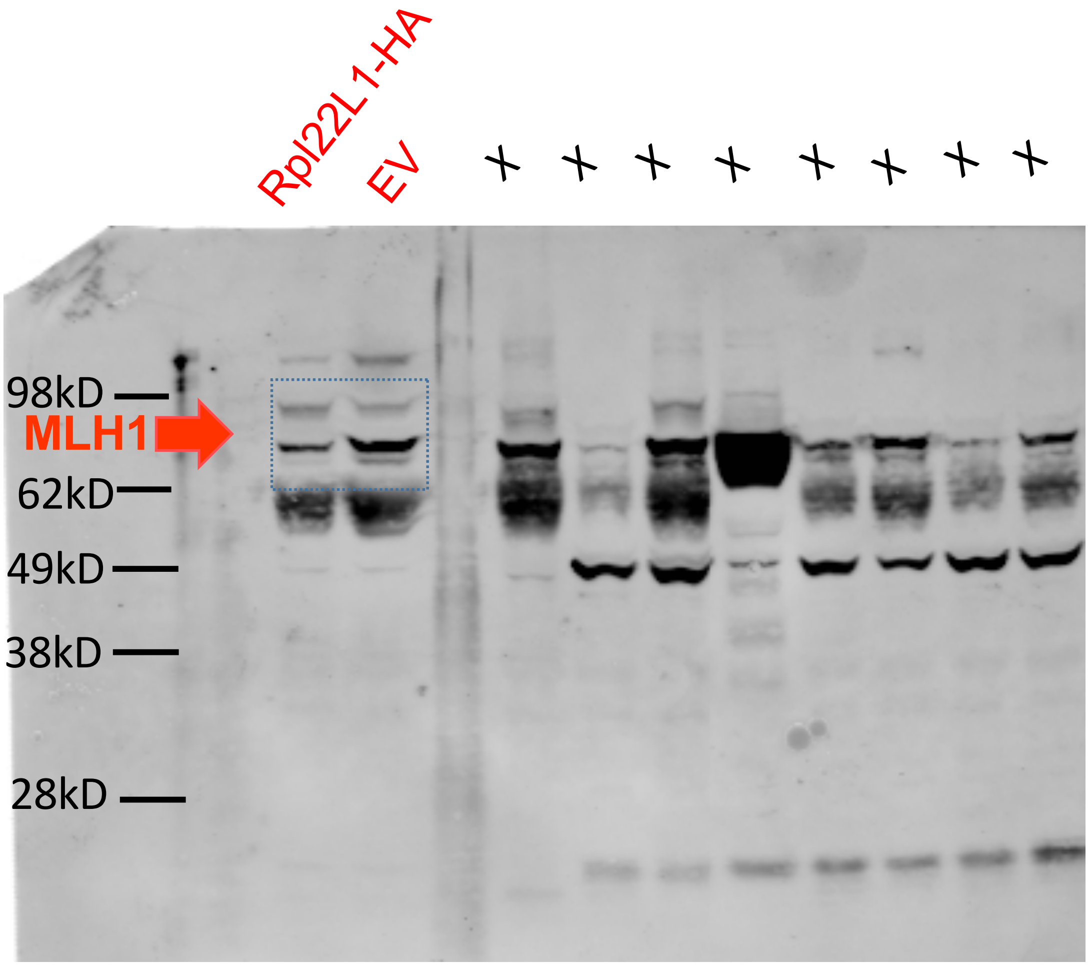

Figure 6A Rpl22L1-HA and Actin in ModeK cells: Raw western blot of **anti-HA** to detect Rpl22L1-HA (red band with red arrow) and **anti-Actin** (red band with red arrow) in cell lysates from ModeK cells with control vector (EV) or Rpl22L1-HA for Fig 6A. Image was developed with LI-COR Odyssey infrared (IR) fluorescence image system. Membrane was cut before antibodies application. The image was captured together with other membranes shown in Fig 1B with anti-Actin, anti-Rpl22 or anti-Rpl22L1. Square indicates the bands used in Fig 6A.

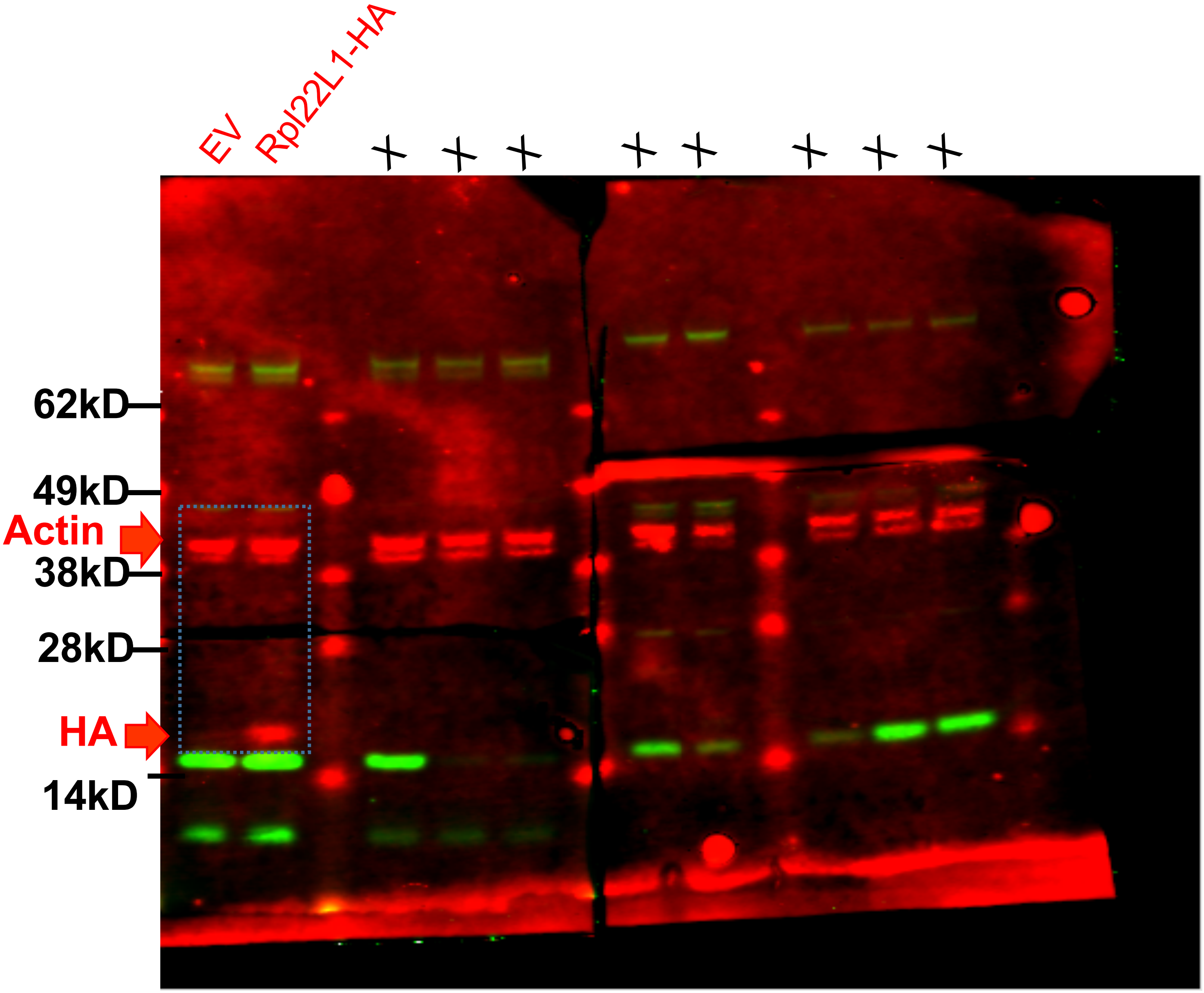

Figure 6A MSH2: Raw western blot of **anti-MSH2** from ModeK cells with control vector (EV) or Rpl22L1-HA for Fig 6A. Image was developed with LI-COR Odyssey infrared (IR) fluorescence image system. Membrane was cut before antibodies application and only this part of membrane was scanned. Square indicates the band used in Fig 6A.

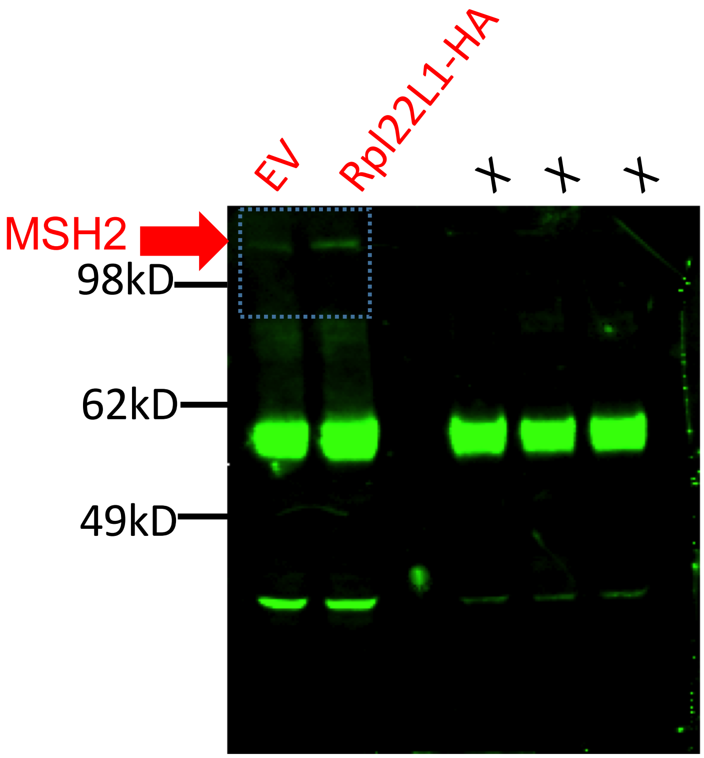

Figure 6B Actin: Raw western blot of **anti-Actin** (red band with red arrow) in cell lysates from HCT116 cells with control shRNA and Rpl22L1 shRNA for Fig 6B. Image was developed with LI-COR Odyssey infrared (IR) fluorescence image system. Square indicates band used in Fig 6B.

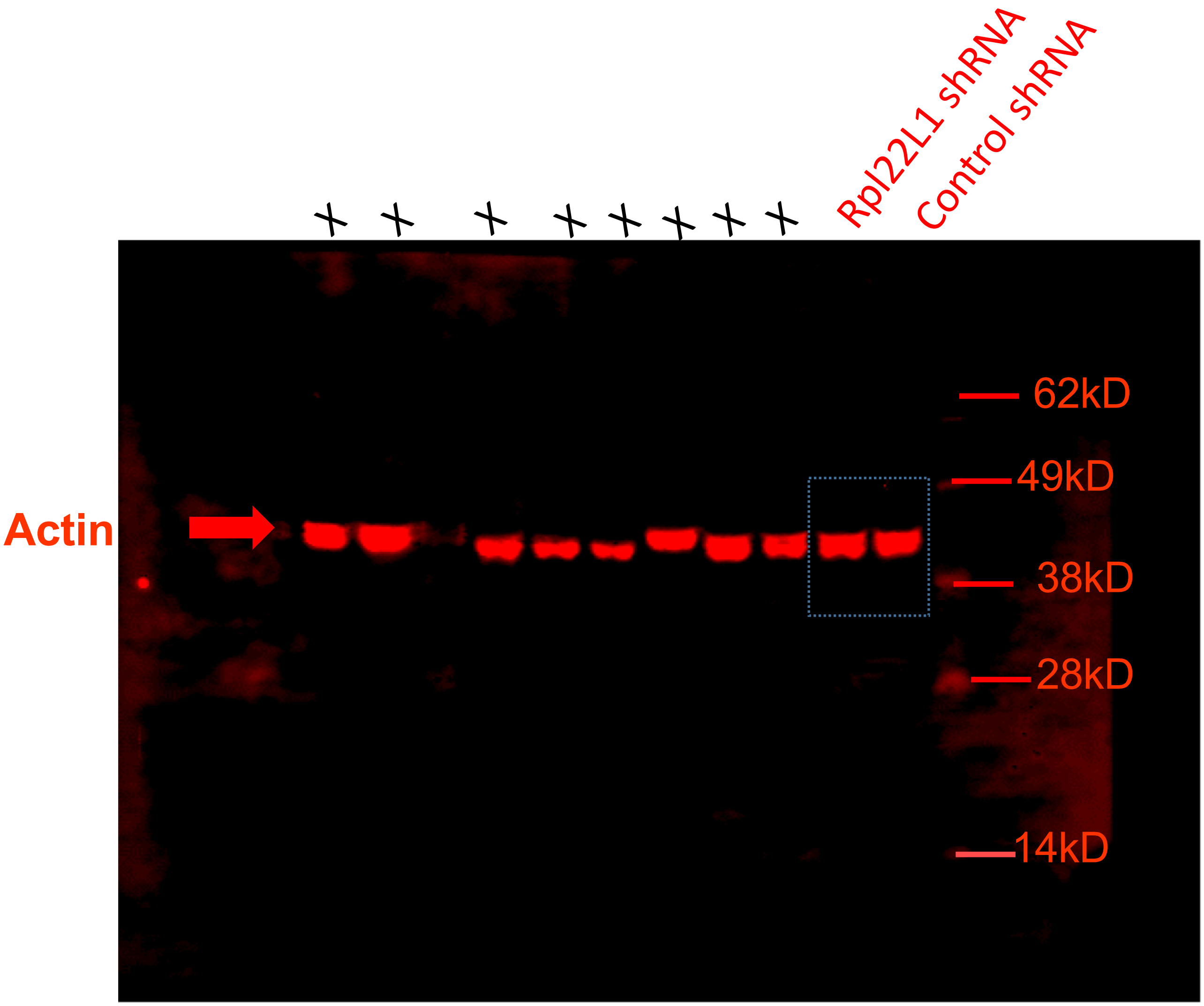

Figure 6B MGMT: Raw western blot of **anti-MGMT** (green band with red arrow) in cell lysates from HCT116 cells with control shRNA or Rpl22L1 shRNA for Fig 6B. Image was developed with LI-COR Odyssey infrared (IR) fluorescence image system. Square indicates band used in Fig 6B.

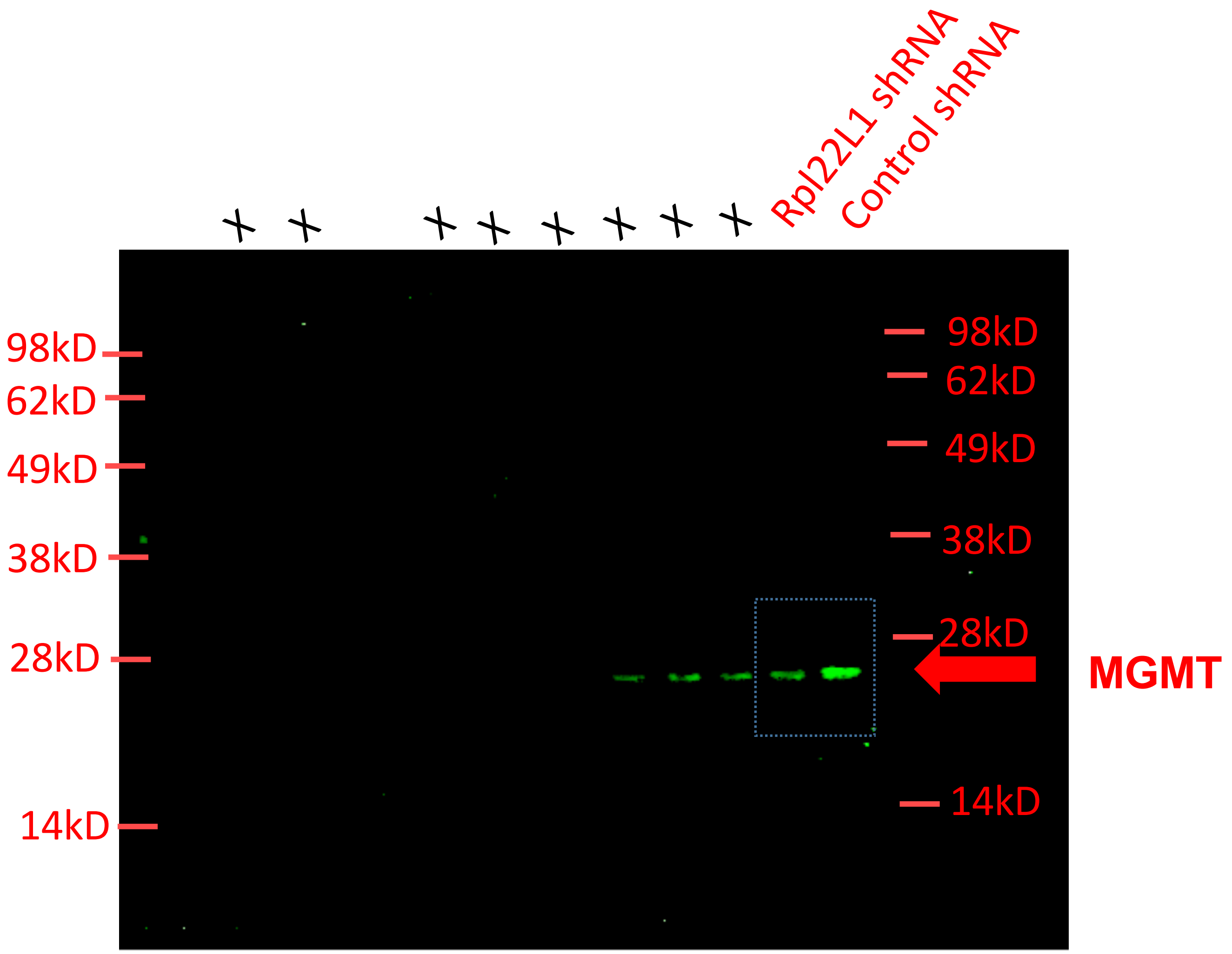

S1C Figure Rpl22L1: Raw western blot of **anti-Rpl22L1** in tissue lysates from indicated organs isolated from wild type (Rpl22+/+, top left image) or Rpl22 knockout mice (Rpl22-/-, bottom left image). Image was developed with luminol-based Enhanced Chemiluminescent (ECL) western blotting substrate. Film was developed together with other membranes (X) at the same time. Square indicates the band used in S1C Fig.

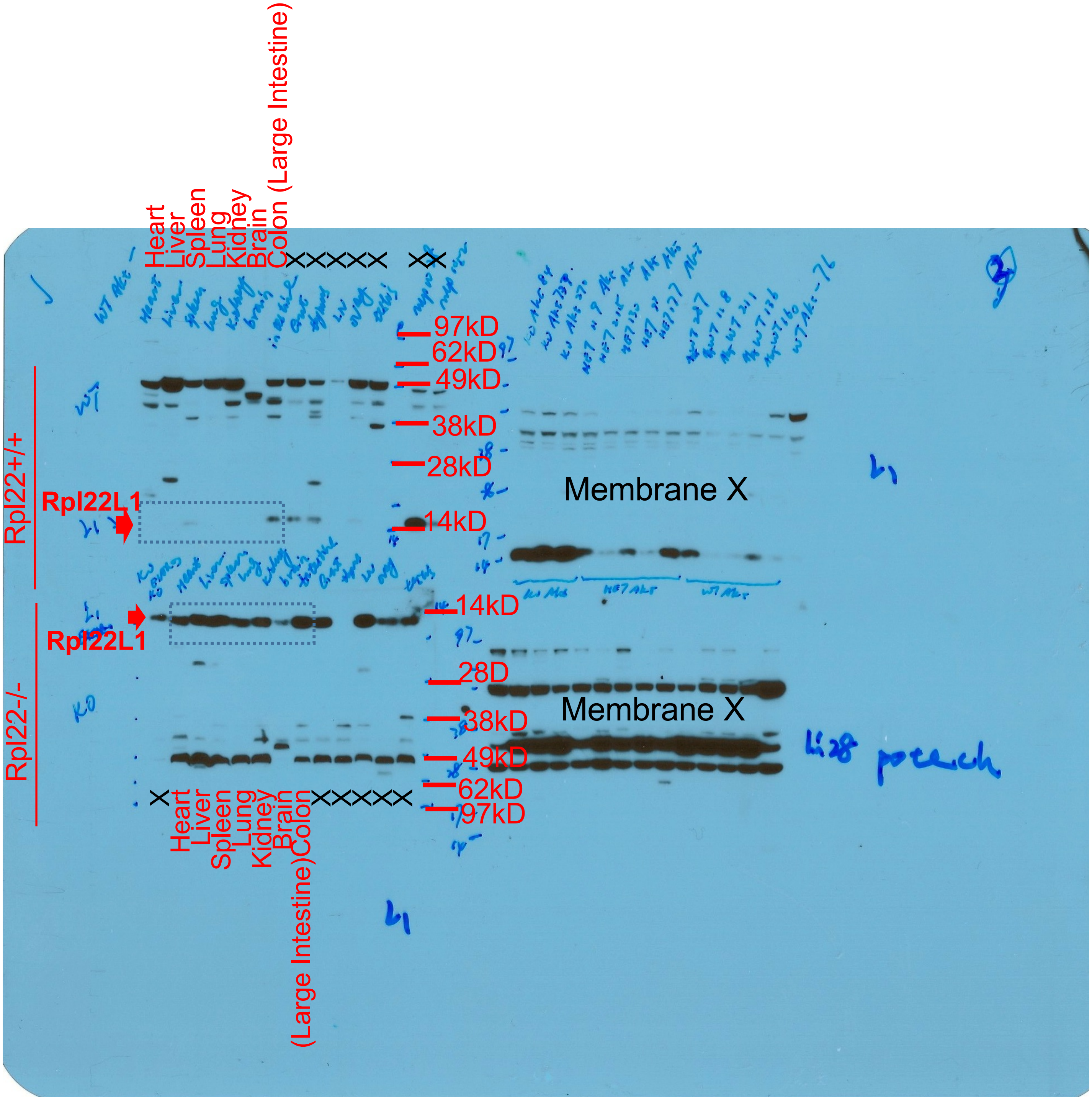

S1C Figure GAPDH: Raw western blot of **anti-GAPDH** in tissue lysates from indicated organs isolated from wild type (Rpl22+/+, top left image) or Rpl22 knockout mice (Rpl22-/-, bottom left image). Image was developed with luminol-based Enhanced Chemiluminescent (ECL) western blotting substrate. Film was developed together with other membranes (X) at the same time. Image was scanned in selected area. Square indicates the band used in S1C Fig.

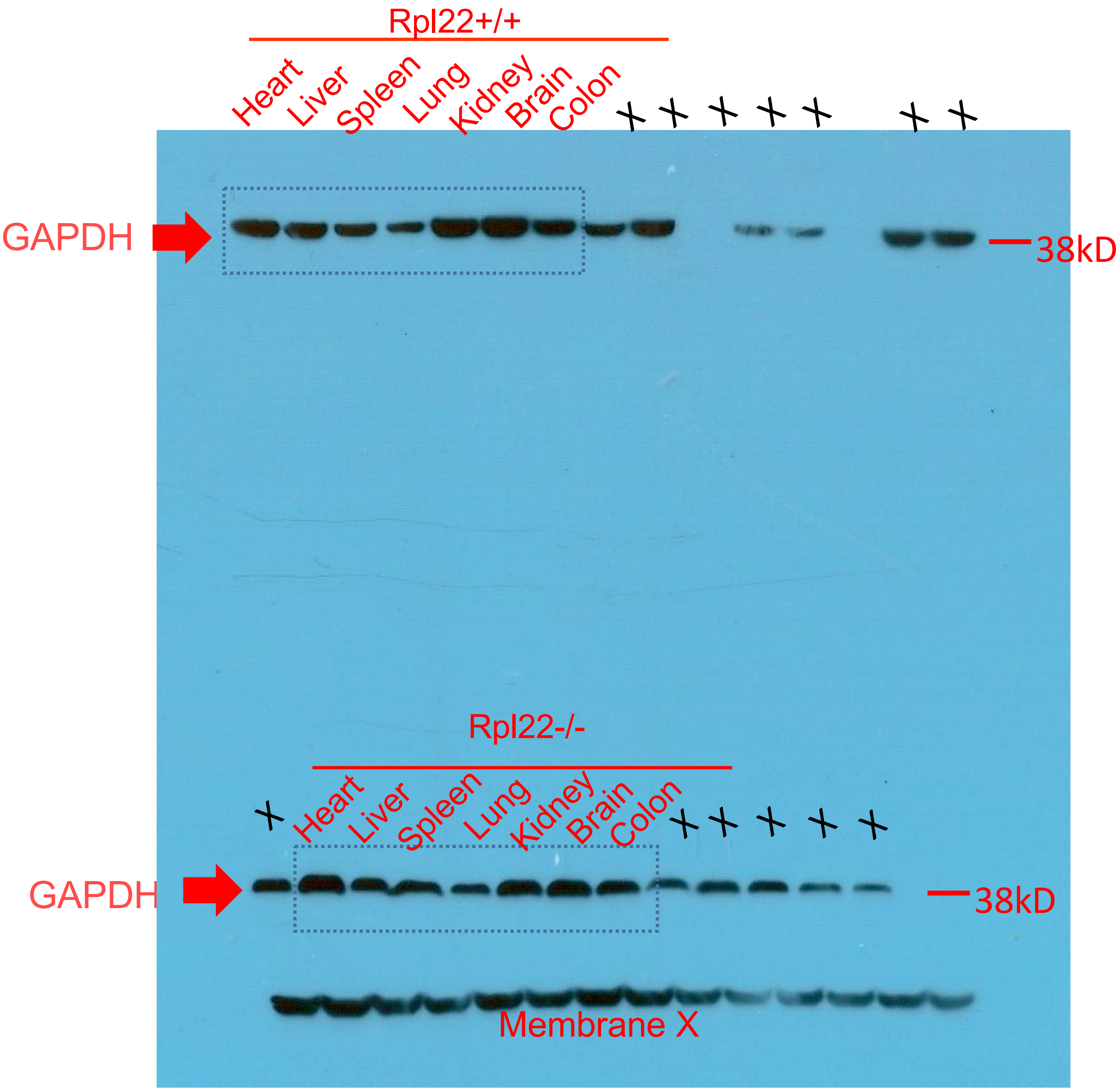

S1D Figure Actin, Rpl22L1, and Rpl22: Raw western blot of **anti-Actin** (red band with red arrow), **anti-Rpl22L1** (green band with red arrow) and anti-Rpl22 (green band with red arrow) in cell lysates from HCT116 cells and CCD841-CoN cells for S1D Fig. Image was developed with LI-COR Odyssey infrared (IR) fluorescence image system. Membrane was cut before antibodies application. For the bottom image to detect Rpl22 and Rpl22L1, only this part of the membrane was scanned. Square indicates bands used in S1D Fig.

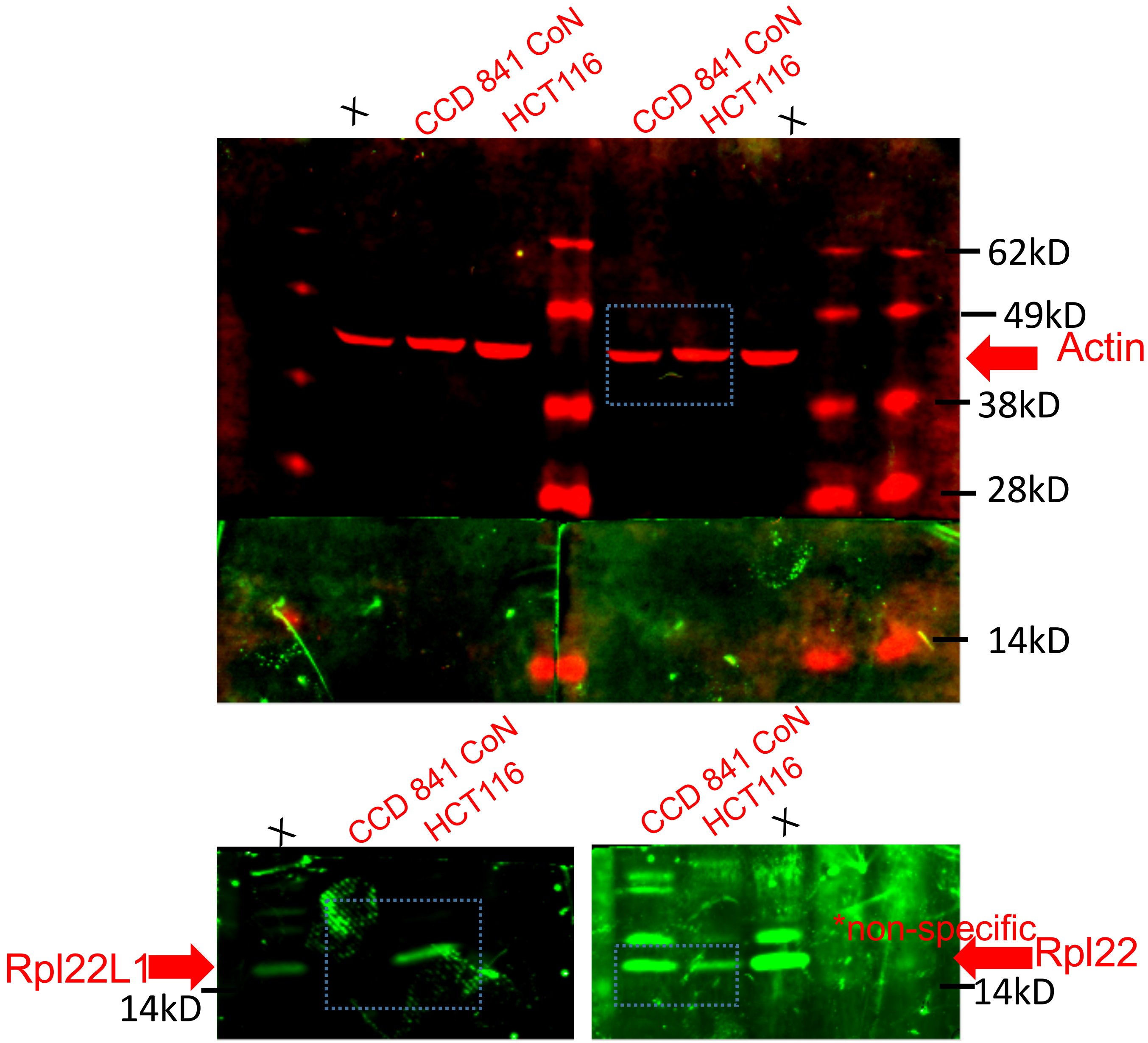

S1E Figure GAPDH: Raw western blot of **anti-GAPDH** in cell lysates from indicated cell lines derived from normal colon or colon cancer for S1E Fig. Image was developed with luminol-based Enhanced Chemiluminescent (ECL) western blotting substrate. Film was cut before the imaging development together with other membranes (X) at the same time. Square indicates the band used in S1E Fig.

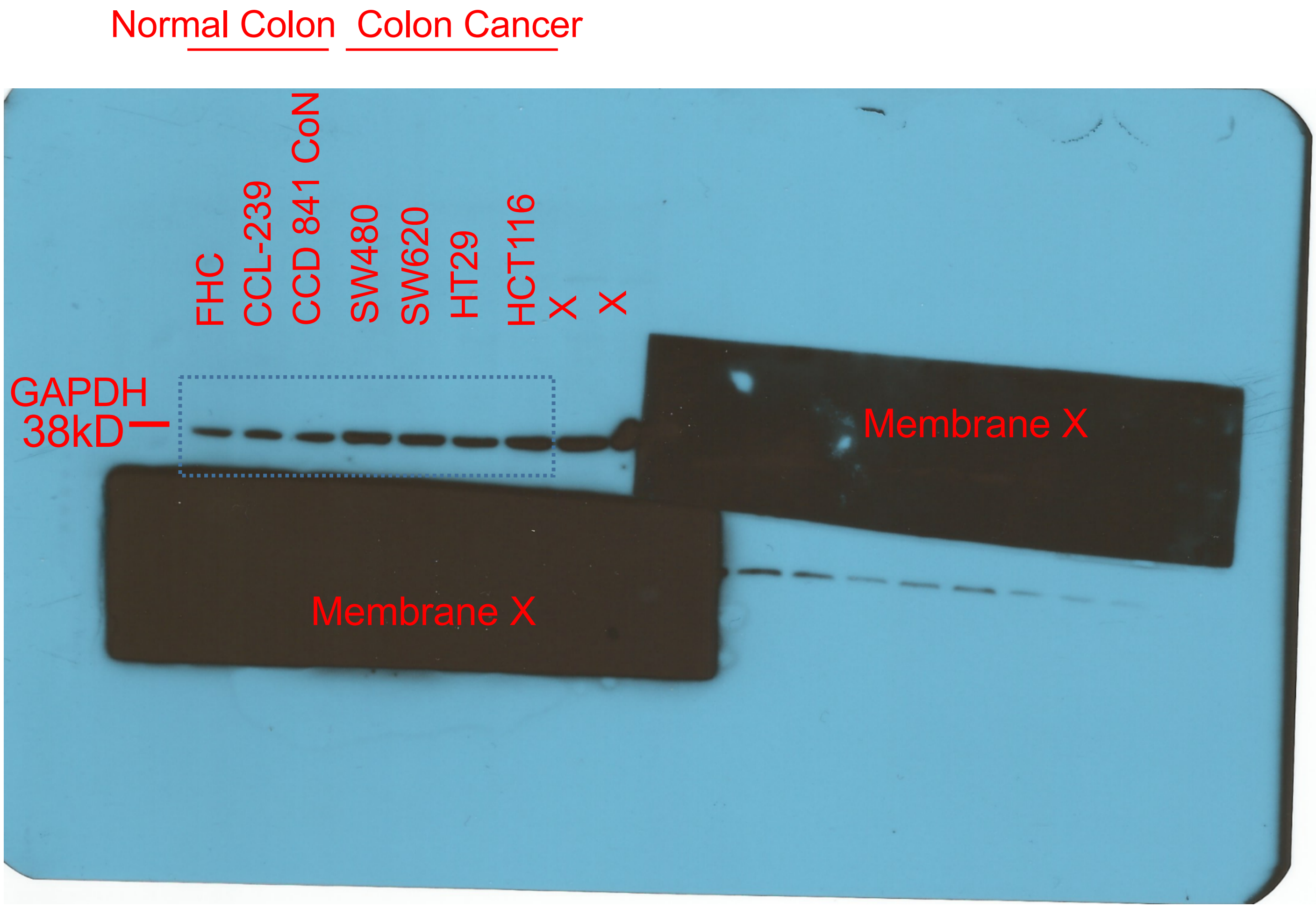

S21E Figure Rpl22L1: Raw western blot of **anti-Rpl22L1** (red arrow) in cell lysates from indicated cell lines derived from normal colon or colon cancer for S1E Fig. Image was developed with luminol-based Enhanced Chemiluminescent (ECL) western blotting substrate. Film was cut before the imaging development together with other membranes (X) at the same time. Square indicates the band used in S1E Fig.

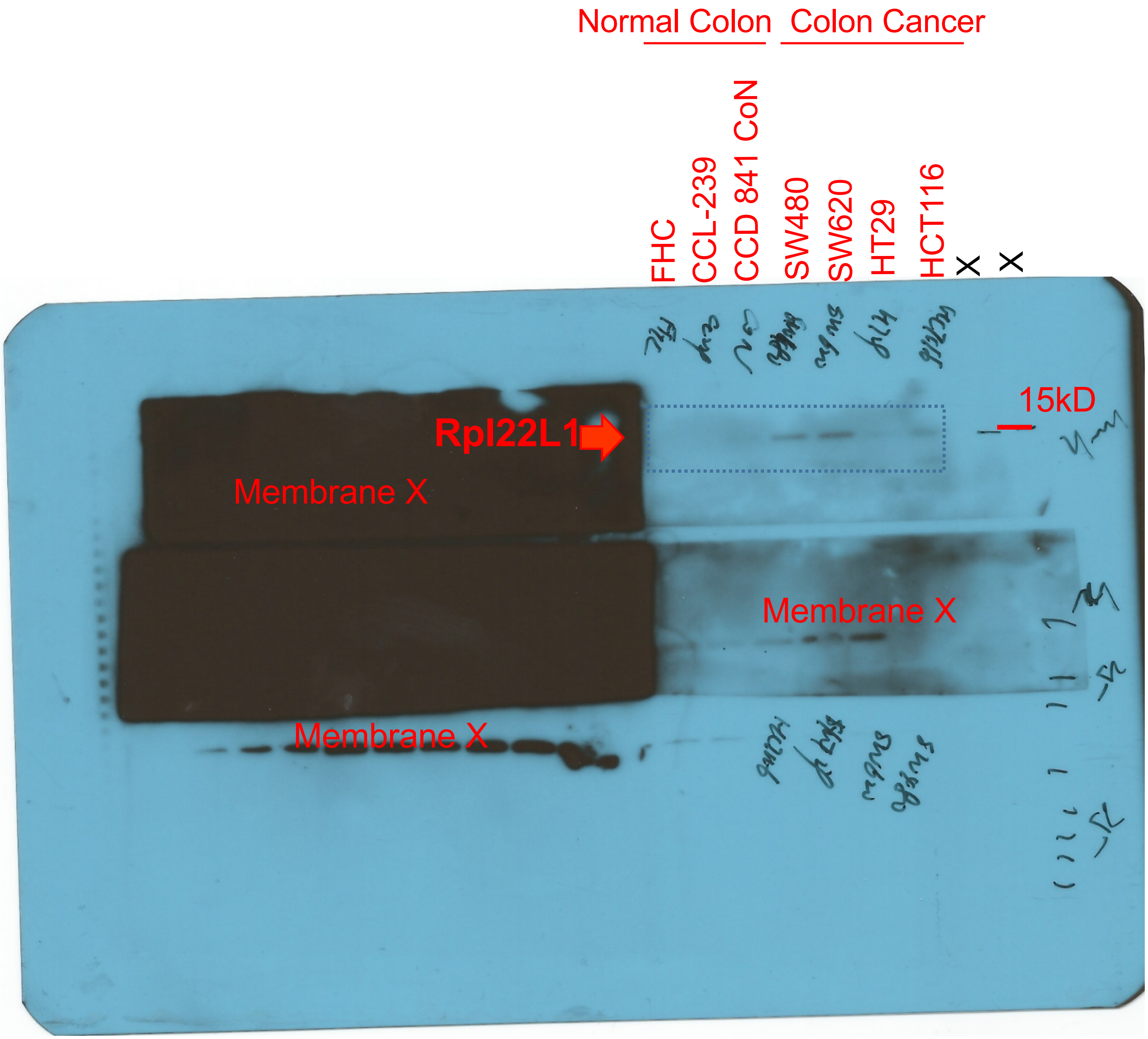

S21E Figure Rpl22: Raw western blot of **anti-Rpl22** (red arrow) in cell lysates from indicated cell lines derived from normal colon or colon cancer for S1E Fig. Image was developed with luminol-based Enhanced Chemiluminescent (ECL) western blotting substrate. Film was cut to incubate with different antibodies before the imaging development together with other membranes (X). Square indicates the band used in S1E Fig.

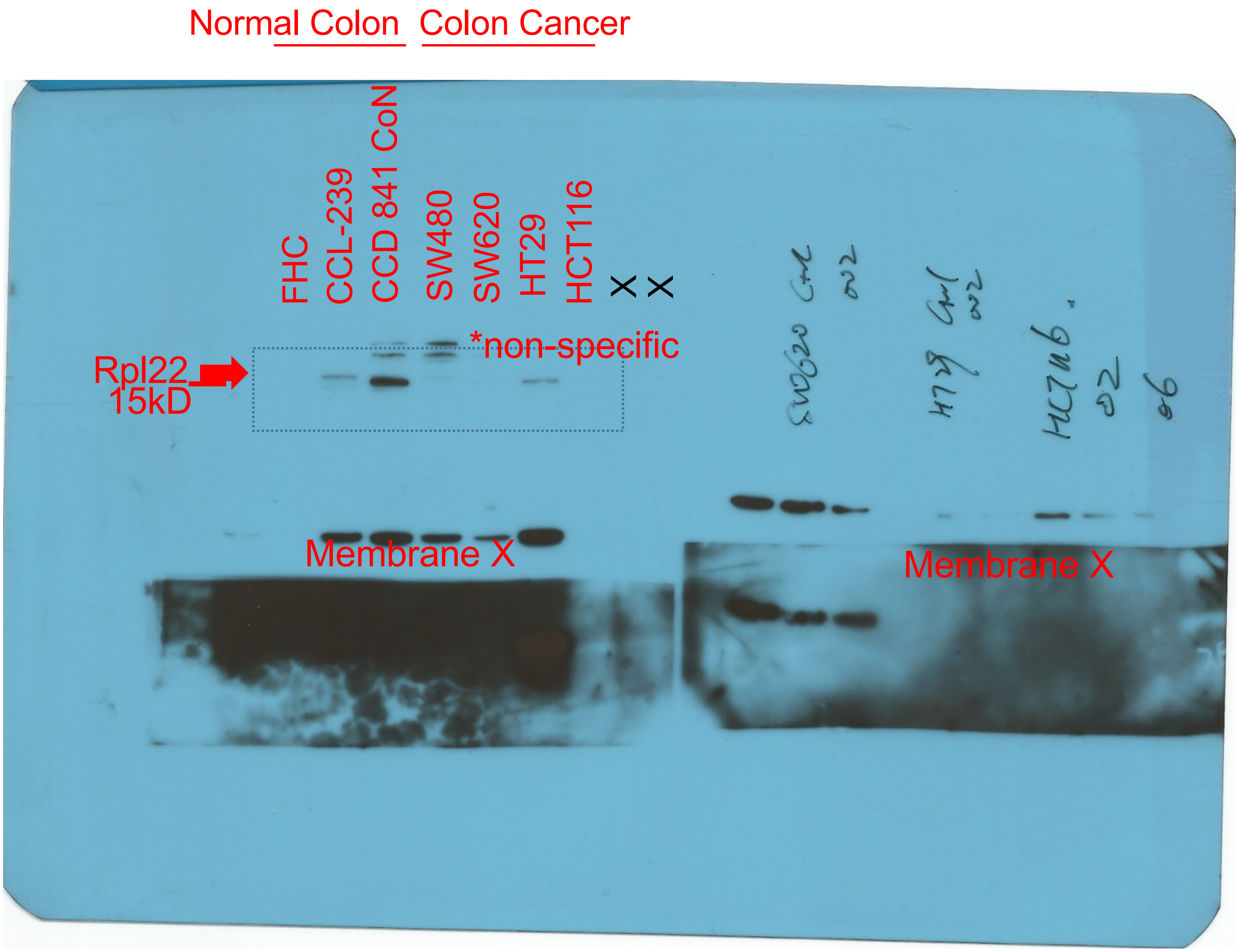

S2C Fig Actin: Raw western blot of **anti-Actin** (red arrow) in lysates from different colon cancer cell lines including HCT116 with control shRNA, Rpl22L1 shRNA1 or Rpl22L1 shRNA2 for S2C Fig. Image was developed with luminol-based Enhanced Chemiluminescent (ECL) western blotting substrate. Film was cut before the imaging development together with other membranes (X) at the same time. Square indicates the band used in S2C Fig.

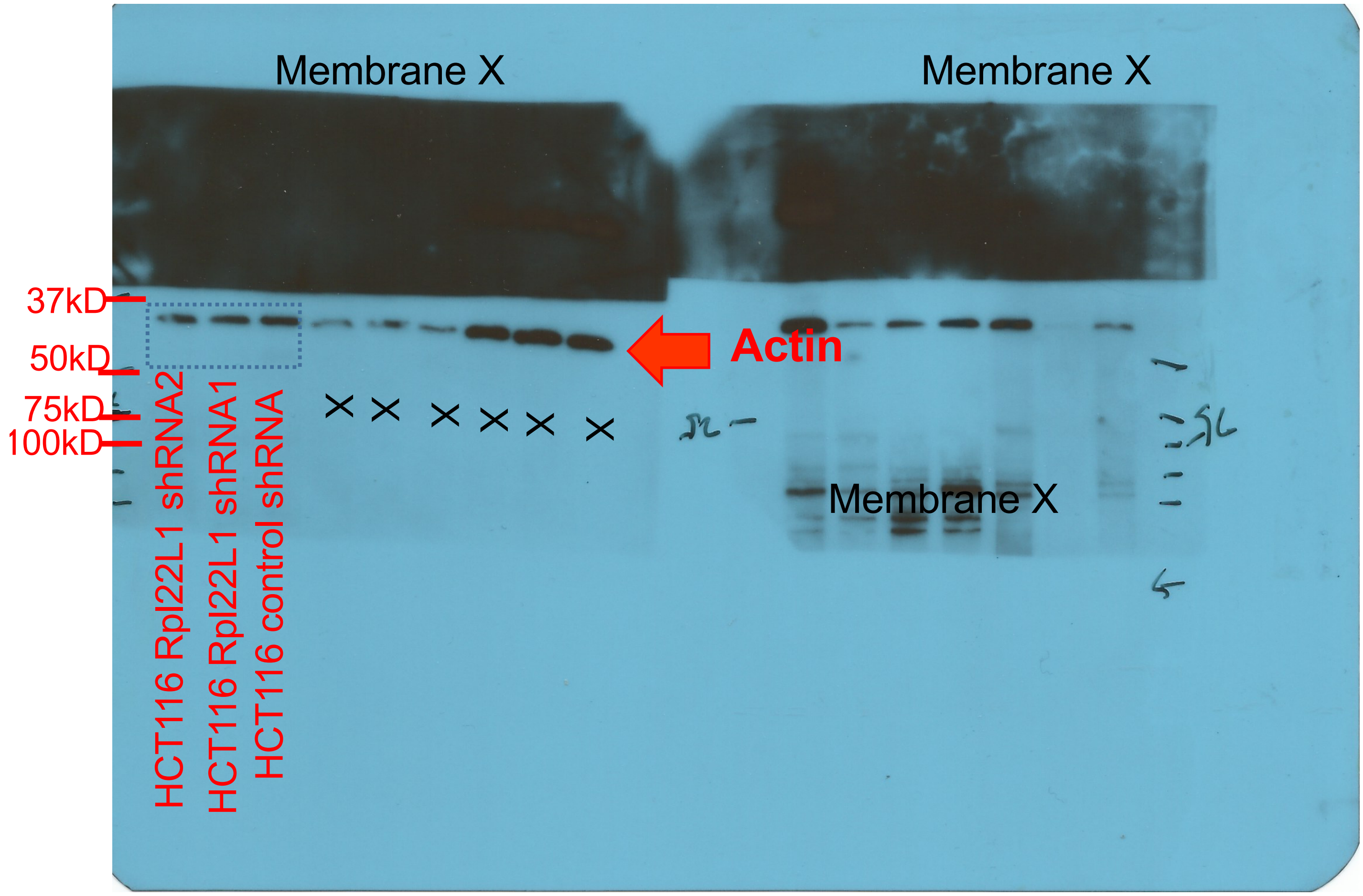

S2C Fig Rpl22L1: Raw western blot of **anti-Rpl22L1** (red arrow) in lysates from different colon cancer cell line including HCT116 with control shRNA, Rpl22L1 shRNA1 or Rpl22L1 shRNA2 for S2C Fig. Image was developed with luminol-based Enhanced Chemiluminescent (ECL) western blotting substrate. Film was cut before the imaging development together with other membranes (X) at the same time. Square indicates the band used in S2C Fig.

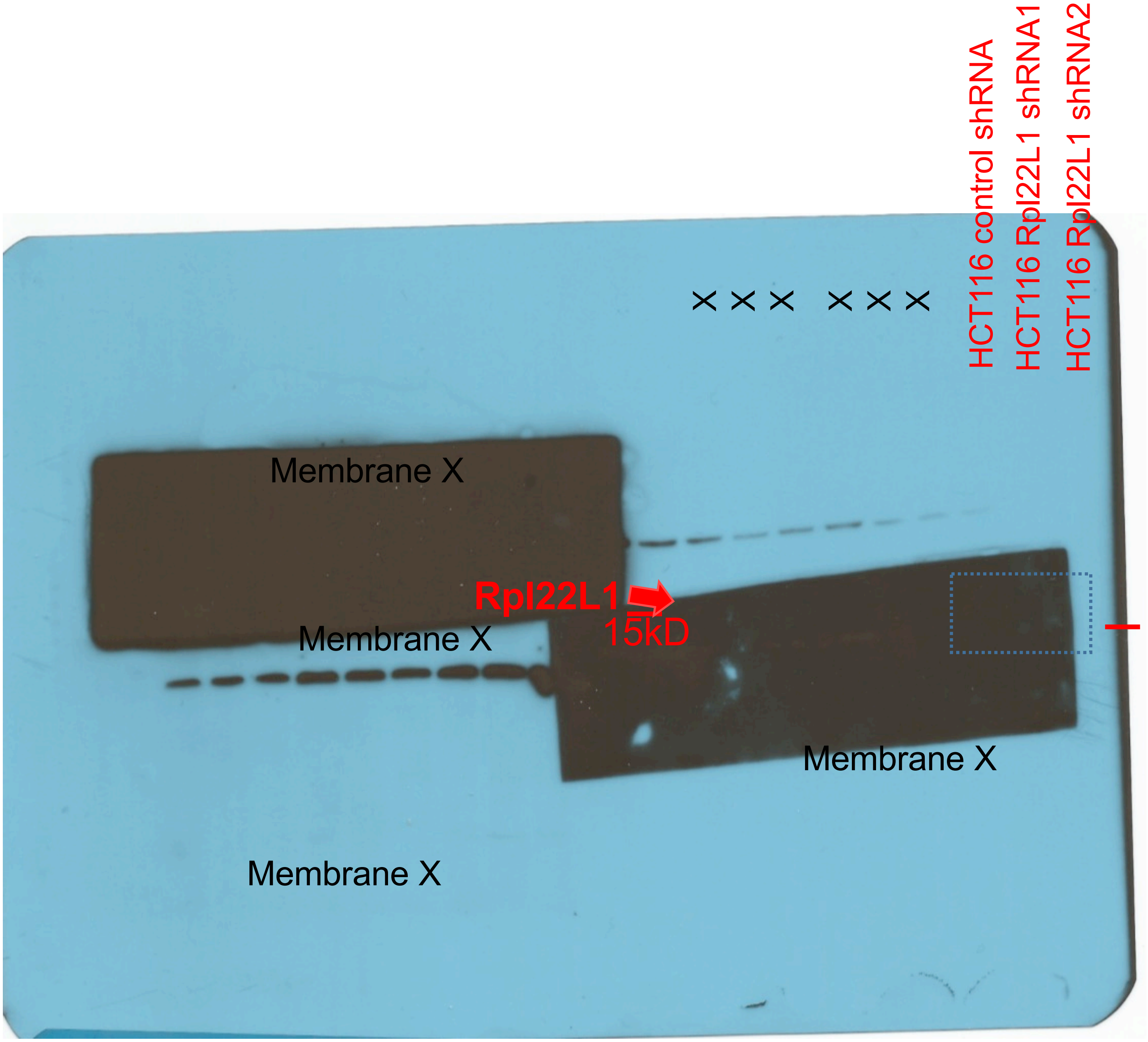

S3A Fig Rpl22L1: Raw western blot of **anti-Rpl22L1** (red arrow) in cell lysates from Hek293 cells with control shRNA, Rpl22L1 shRNA1 and Rpl22L1 shRNA2 for S3A Fig. Image was developed with luminol-based Enhanced Chemiluminescent (ECL) western blotting substrate. Film was cut before the imaging development together with other membranes (X) at the same time. Square indicates the band used in S3A Fig.

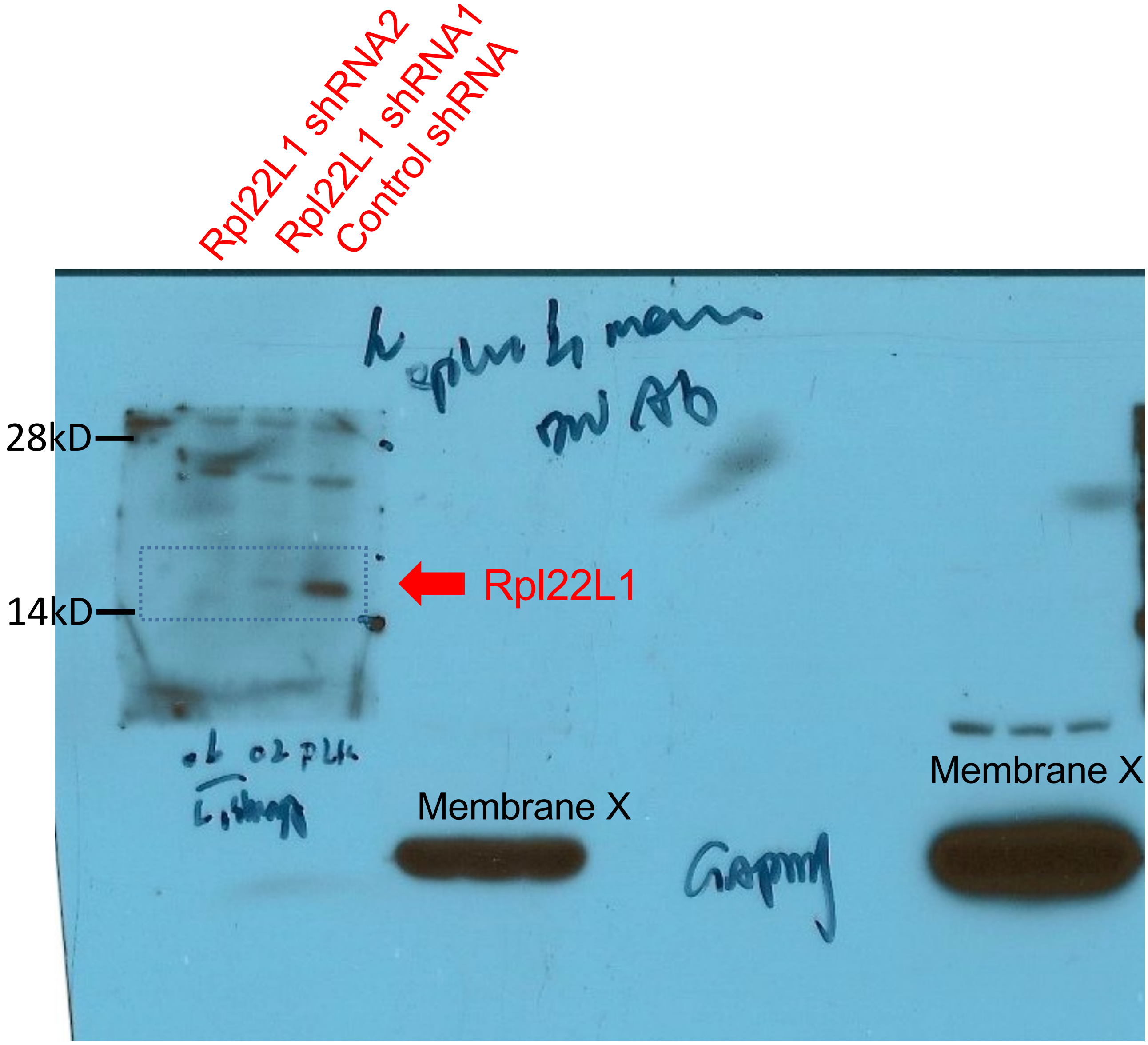

S3A Fig Actin: Raw western blot of **anti-Actin** (read arrow) in cell lysates from Hek293 cells with control shRNA, Rpl22L1 shRNA1 and Rpl22L1 shRNA2 for S3A Fig. Image was developed with luminol-based Enhanced Chemiluminescent (ECL) western blotting substrate. Film was cut before the imaging development. Only this part of membrane was scanned. Square indicates the band used in S3A Fig.

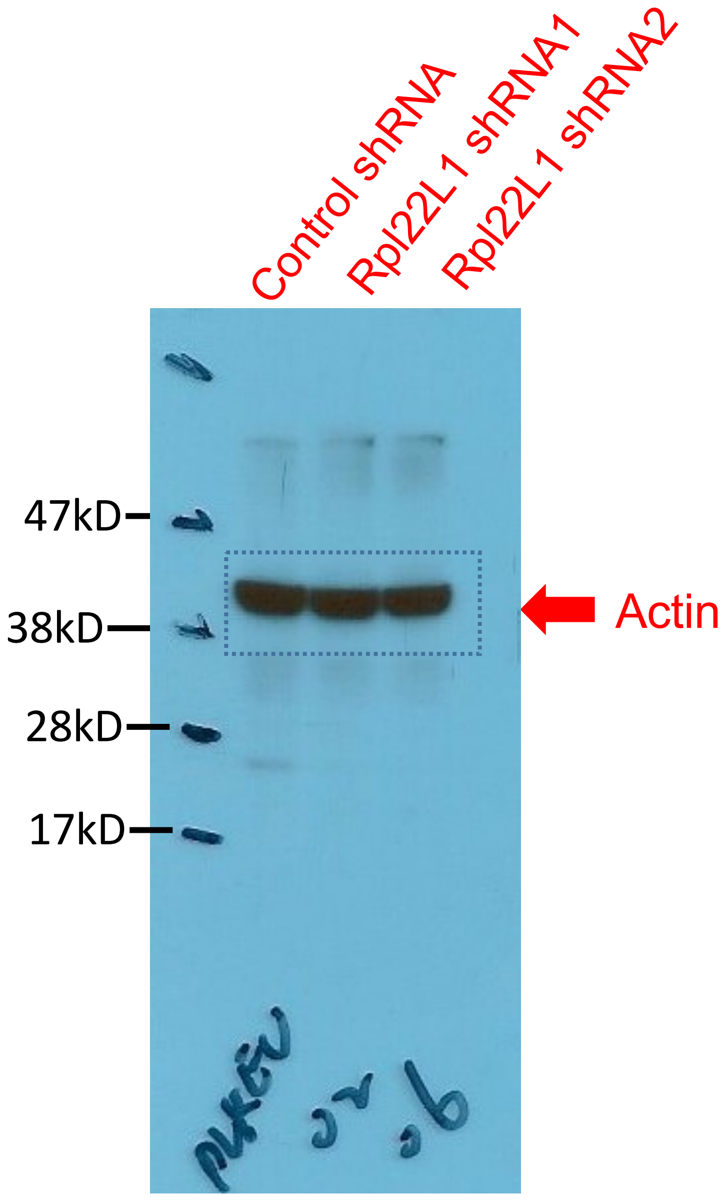

S6B Fig Rpl22L1 and Actin: Raw western blot of **anti-Rpl22L1** (green band with red arrow) and **anti-Actin** (red band with red arrow) in HCT116 and ModeK control cells (Ctrl) or cells developed 5-Fu resistance (Resis) for S6 Fig. Image was developed with LI-COR Odyssey infrared (IR) fluorescence image system. Square indicates bands used in S6B Fig. Membrane was cut before the imaging development with anti-Actin and acti-Rpl22L1 at the same time. Square indicates bands used in S6B Fig.

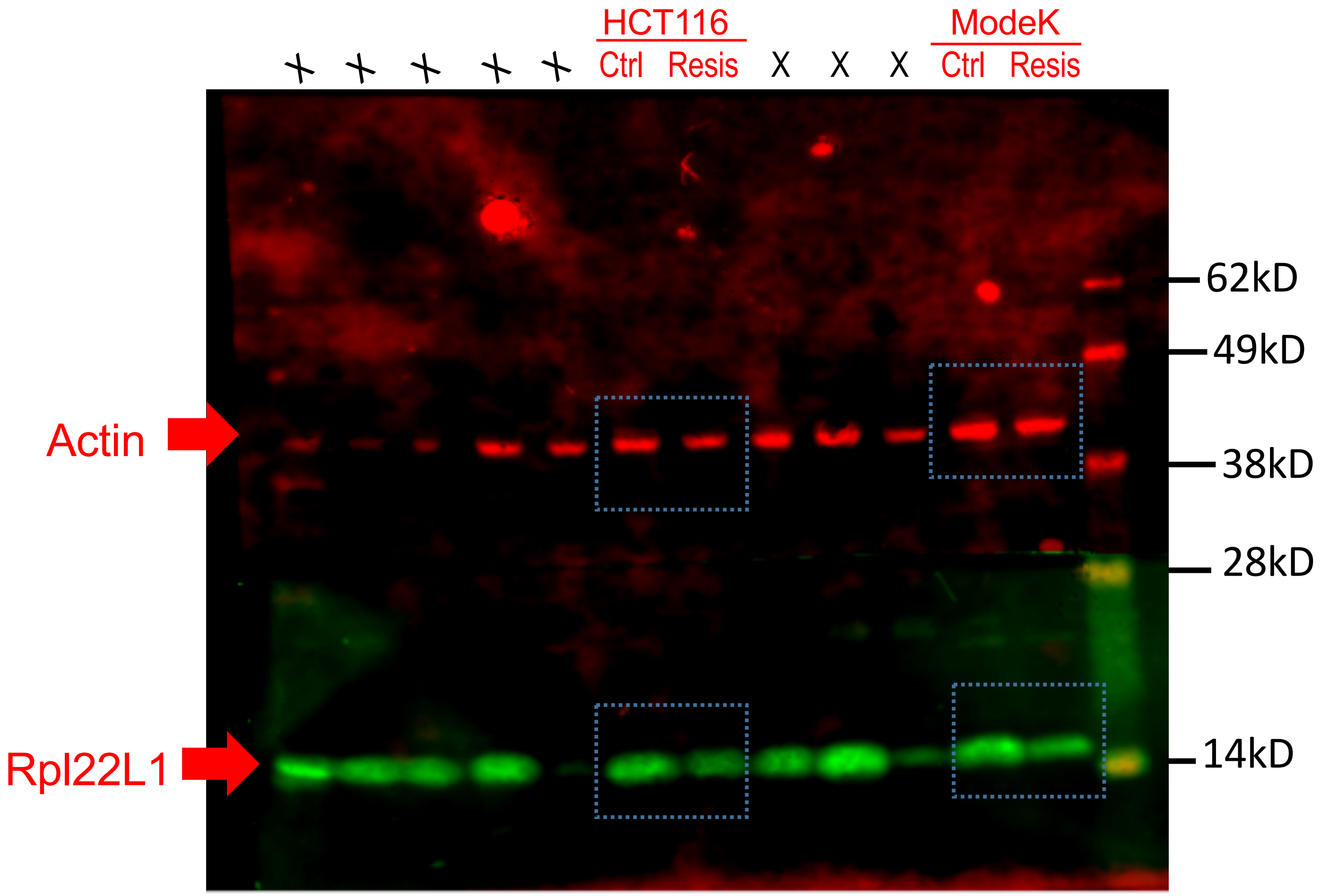

Supplement: S1 Raw Images — (PDF) [file pone.0222392.s011.pdf]
